# Exploring the antioxidant, antimicrobial, cytotoxic and biothermodynamic properties of novel morpholine derivative bioactive Mn(ii), Co(ii) and Ni(ii) complexes – combined experimental and theoretical measurements towards DNA/BSA/SARS-CoV-2 3CL^Pro^†

**DOI:** 10.1039/d2md00394e

**Published:** 2023-01-03

**Authors:** Karunganathan Sakthikumar, Bienfait Kabuyaya Isamura, Rui Werner Maçedo Krause

**Affiliations:** a Organic & Medicinal Chemistry, Department of Chemistry, Center for Chemico- and Biomedicinal Research (CCBR), Faculty of Science, Rhodes University Grahamstown 6140 Eastern Cape South Africa r.krause@ru.ac.za; b Center for Chemico- and Biomedicinal Research (CCBR), Faculty of Science, Rhodes University Grahamstown 6140 Eastern Cape South Africa +27 741622674 +27 46 603 7030; c Department of Chemistry, The University of Manchester Manchester M13 9PL UK

## Abstract

A novel class of bioactive complexes (1–3) [M^II^(L)_2_(bpy)], where, L = 2-(4-morpholinobenzylideneamino)phenol, bpy = 2,2′-bipyridine, M^II^ = Mn (1), Co (2) or Ni (3), were assigned to octahedral geometry based on analytical and spectral measurements. Gel electrophoresis showed that complex (2) demonstrated significant DNA cleavage activity compared to the other complexes under the action of oxidation agent (H_2_O_2_). The DNA binding constant properties measured by various techniques were in the following sequence: (2) > (3) > (1) > (**HL**), which suggests that the complexes might intercalate DNA, a possibility that is also supported by their biothermodynamic characteristics. The binding constant results for BSA from electronic absorption and fluorometric titrations demonstrate that complex (2) exhibits the highest binding effectiveness among them all, which means that all the compounds could interact with BSA through a static approach, additionally supported by FRET measurements. DFT and docking calculations were employed to realize the electronic structure, reactivity, and interaction capability of all substances with DNA, BSA, and the SARS-CoV-2 main protease. These binding energies fell within the ranges −7.7 to −8.5, −8.2 to −10.1 and −6.7 to −9.3 kcal mol^−1^, respectively. The higher reactivity of the complexes than the ligand is supported by FMO theory. The *in vitro* antibacterial, cytotoxicity, and radical scavenging characteristics revealed that complexes (2–3) have better biological efficacy than the others. The cytotoxicity and binding properties also show good correlation with the partition coefficient (log *P*), which is encouraging because all of the experimental findings are closely correlated with the theoretical measurements.

## Abstract 2

Mn(ii), Co(ii), and Ni(ii) morpholine and 2,2′-bipyridine complexes gave potent DNA cleavage, antimicrobial and antioxidant activity. Calculations support the results, with the Co and Ni complexes showing better anticancer efficacy than cisplatin.

## Graphical abstract

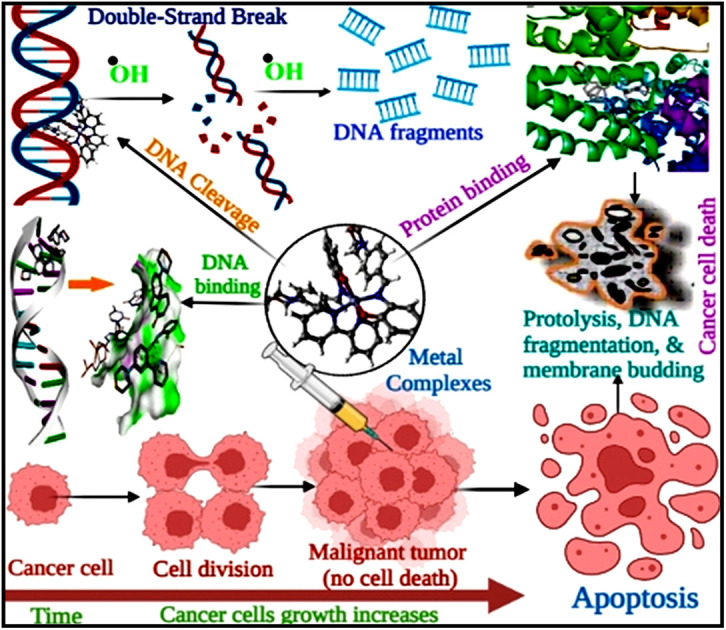

## 1. Introduction

Despite significant advances over the last five decades in conjunction with surgical resectioning, radiotherapy, chemotherapy, immunotherapy, hormone therapy, targeted drug therapy, and cryoablation, cancer is one of the leading global causes of death today. It denotes the uncontrollable expansion of aberrant cells that can invade and disturb tissues. This can also lead to a number of microbial diseases, which greatly increases this burden.^1,2^ Over the past two decades, there have been significant advances in almost every field of science and technology. However, these advanced treatments for microbial infections and cancer are still far from complete. Cancer and bacterial infections undoubtedly pose a serious threat to people's health and present a problem for our society. However, the use of currently available antimicrobial and anticancer medications is limited due to their toxicity and drug resistance.^3^ An excess of these medications is already on the market to treat various disorders, especially with the development of transition-metal-based anticancer and antimicrobial prodrugs, which currently show significant promise. However, due to the widespread incidence of multidrug resistance in cancer and microbial infections, it is essential to create new and promising compounds with desirable qualities that might address multidrug resistance and toxic profiles.^4^ Platinum-based drugs are currently available for the treatment of cancer chemotherapy and account for nearly 50% of cancer therapeutic medications globally, but the majority of these unfortunately have a lot of negative side effects, being extremely toxic and drug resistant, and they lose selectivity in chemotherapy due to the formation of covalent interactions.^5,6^ To overcome these drawbacks, transition metal complexes other than platinum have attracted particular attention due to their diverse oxidation states and lower toxicity.^7,8^

Moreover, free radicals play a role in several aspects of the body's normal oxygen metabolism, including vasodilation (blood vessel dilatation), the immune response, cell differentiation, and electron transfer in the mitochondrial respiratory chain. Oxidative stress is caused by an imbalance between the production and detoxification of free radical species. This condition has the potential to seriously harm proteins, lipids, and DNA, which may result in the emergence of serious diseases. Finding novel metal complexes with both antioxidant and antimicrobial properties is therefore to be encouraged.^9^ In addition, highly oxidizing compounds that produce ROS include O_2_˙^−^, H_2_O_2_, OH˙, ROOH, ROO˙, HOCl, and ^1^O_2_ and O_3_, which also play essential roles in living systems and induce the death of cancer cells oxidatively. Mitochondria play a major role in controlling the production of ROS for cellular signaling in numerous physiological processes. Compared to normal cells, anticancer drugs demonstrate selectivity for cell targets with abnormal ROS levels, and they kill tumor cells with abnormal redox functioning.^10^ However, transition metal complexes are crucial to nucleic acid chemistry due to their numerous uses as therapeutic agents, structural probes, footprinting agents, and sequence-specific binding.^11^ They can also be used as scaffolds for pharmacological agent because of their inertness, stability, distinctive geometries, and structural diversity.^12^ Moreover, chelation effectively alters both the biological characteristics of the metal moiety and the ligands. The excellent metal chelating abilities of morpholine derivative compounds, which are also regarded as multifaceted ligands owing to their synthetic flexibility and conformational stability, contribute to the enhanced biological activity with the coordination of metal centers. As a result, these compounds are being thoroughly studied in light of their outstanding pharmacological activities.^13,14^

Furthermore, the significant effects of transition metal complexes are expected to exhibit their potent effects by a variety of mechanisms, including enzyme inhibition, intracellular biomolecular interactions, increased lipophilicity, modifications to cell membrane functions, and cell cycle arrest. The selectivity of antimicrobial and anticancer drugs is improved by the following mechanisms: disruption of cell membranes and inhibition of nucleic acid/protein/cell wall synthesis. However, a significant fraction of antibiotics used therapeutically work against tuberculosis by specifically targeting the ribosomal RNA-rich surfaces of ribosomes, and mostly inhibit protein synthesis.^15–17^ Furthermore, DNA serves as the primary target site of action for the majority of anticancer medications. The anticancer property of mononuclear metallodrugs is ascribed to their interacting with DNA either covalently or noncovalently. Covalent interactions take place *via* labile ligands of complexes that are transferred by the N7 donor atoms of DNA's guanine/adenine bases. The complexes are involved in noncovalent interactions, such as electrostatic, H-bonding, and π–π stacking interactions, which provide further stability to these adducts.^18^ Subsequently, the bindings of transition metal complexes with DNA/RNA have been widely investigated. The highly positively charged transition metal complexes bind electrostatically with negatively charged DNA and RNA, different phospholipids, and some regions of proteins. Furthermore, targeting and activation tactics can also aid the formation of new antibacterial and anticancer medications with the capability of overcoming the limitations of currently available drugs. Moreover, advanced DFT and molecular docking-based virtual screenings are quite helpful and will undoubtedly enhance our comprehension of the chemical and biological reactivity of medications.^19,20^ Considering the above-mentioned approaches, a research scheme has been carried out using a pharmacologically active morpholine-linked primary ligand incorporating 2,2′ bipyridine for the synthesis of metal complexes (1–3) and the research was extended to comprehend the binding between the metal complexes (1–3) and DNA, BSA, and the SARS-CoV-2 (3CL^Pro^) protein *via* molecular docking approaches. They might also support the creation of new, powerful anticancer medications as well as playing a part in the battle against current or prospective viral pandemics.

## 2. Experimental section

### 2.1. Materials and techniques

All chemicals, reagents, and solvents of analytical grade were procured from Sigma-Aldrich, BD Biosciences, and Alfa Aesar. All compounds were examined through a variety of analytical and spectroscopic studies. The details of the experimental section were summarized in previous reports^13,21^ and further deposited as an ESI† file (2a).

### 2.2. Assessment of DNA/BSA binding features

#### 2.2.1. Assessment of DNA nuclease efficacy

All substances were evaluated for DNA cleavage ability and the characteristics were examined for all substances along with DNA by a gel electrophoresis approach under H_2_O_2_ in Tris-HCl buffer solution with a pH of 7.4.^13,22^

#### 2.2.2. Analysis of DNA-interaction characteristics

The DNA-binding experiment was conducted with an electronic absorption spectrophotometer by raising the DNA concentration from zero to 50 μM to the given concentration of all samples (50 μM) in Tris-HCl buffer (5 mM Tris-HCl/50 mM NaCl) with a pH of 7.4 at 25 °C.^13,23–26^

#### 2.2.3. Assessment of biothermodynamic characteristics

The DNA thermal denaturation properties were measured with an electronic absorption spectrophotometer in the presence and absence of the substances. In a 5 mM Tris-HCl/50 mM NaCl buffer solution with a pH of 7.4, CT-DNA was treated with all test substances in a 1 : 1 ratio (50 μM).^13,27–29^

#### 2.2.4. Assessment of DNA affinity by a hydrodynamic technique

The hydrodynamic properties were measured using an Ostwald viscometer with the help of a thermostat (25 ± 0.1 °C). The specific viscosity of DNA was also measured in the presence and absence of the test samples.^13,30^

#### 2.2.5. Assessment of DNA/BSA binding characteristics by a fluorometric technique

Titrations were performed for all tested compounds (1–200 μM) with pre-incubated EB-bound DNA and the intensity variations between 510 nm and 610 nm were carefully monitored in the presence and absence of DNA (200 μM) during the initial emission and excitation by EB.^31^ Also, emission spectral titration was also carried out for all compounds at a fixed concentration (25 μM) of BSA with an incremental concentration of the substances (0–25 μM) in a Tris-HCl buffer solution with a pH of 7.4, and the binding ability of all samples with BSA was examined at a fixed excitation wavelength of 278 nm and the emission observed at 350 nm.^13,32^

#### 2.2.6. Förster's theory-based FRET computation

The critical distance between donor and acceptor molecules can be estimated using the FRET approach to assess the binding affinity between BSA and a test substance.^13,33–35^

#### 2.2.7. Analysis of DNA binding characteristics using cyclic voltammetry (CV)

CV analysis for free substances was performed at 10 μM at 25 °C in a 5 mM Tris-HCl buffer solution with a pH of 7.4. The changes in peak current as well as peak potential were monitored while concentrations of CT-DNA were increased (0–10 μM) in each sample solution.^13,36^

#### 2.2.8. Assessment of BSA binding characteristics by electronic absorption titration

The absorption titrations were done with a 25 μM concentration of BSA at 25 °C in a Tris-HCl buffer solution with a pH of 7.4. While the sample concentrations (0–25 μM) increased in solution with the same BSA concentration, the change in the absorption band at 278 nm was continuously measured.^13,37^

### 2.3. DFT and molecular docking simulations

To validate the results found from the experimental studies, the synthesized compounds were further investigated for their interaction with DNA/BSA/SARS-CoV-2 3CL^Pro^. All test compounds were fully optimized with the help of the hybrid B3LYP functional as accomplished in the Gaussian 09 package.^38^ To demonstrate the global and local reactivity of all substances, frontier molecular orbital (FMO) theory^39^ and molecular electrostatic potentials^40^ were studied. Using the B3LYP-optimized structures of each substance, docking computations were also carried out. Autodock Vina software was used for the preparation of input structures and calculations^41^ and the visualization was performed on Discovery Studio.^42^

### 2.4. UV-vis absorption titrations for *in vitro* antioxidant assay

All samples were evaluated for their scavenging abilities using the UV-vis absorption titrations at different concentrations of 40, 80, 120, 160, 200, and 240 μM. While studying the antioxidant properties for DPPH˙, OH˙, SO_2_˙^−^, and NO˙ radical scavenging, the absorbance at 517, 230, 590, and 546 nm, respectively, was closely observed. The observed IC_50_ values of all samples were further compared with those of standard ascorbic acid.^13,43,44^

### 2.5. Assessment of *in vitro* antimicrobial properties

*In vitro* antimicrobial properties were evaluated for all samples by the agar disc diffusion method against some selected fungal and bacterial strains.^13,45–47^

### 2.6. MTT cell viability assay for anticancer characteristics

All substances directed towards the A549, HepG2, MCF-7, and NHDF cell lines were evaluated by the MTT approach. The collected data (mean O.D. ± S.D) was utilized to compute the IC_50_ value compared with standard cisplatin anticancer medication.^13,48^

### 2.7. Determination of lipophilicity (hydrophobicity)

The lipophilicity of all complexes and free ligands was evaluated by the flask-shaking method through *n*-octanol/deionized water phase partition.^49–51^ The partition coefficients (log *P*_o/w_) and distribution coefficients (log *D*_o/w_) of all substances were also acquired from molar absorption coefficients, conductivity, and pH measurements.^52–54^

## 3. Results and discussion

At 25 °C, it is observed that all of the compounds are highly pigmented, faintly hygroscopic, and have high solubility in CH_3_OH, C_2_H_5_OH, CHCl_3_, and DMSO. The evaluated analytical results and structural characteristics are presented in the ESI† data file (3a) (Fig. S1–S47 and Tables S1–S15).

### 3.1. Synthetic process and properties

**Scheme 1:**
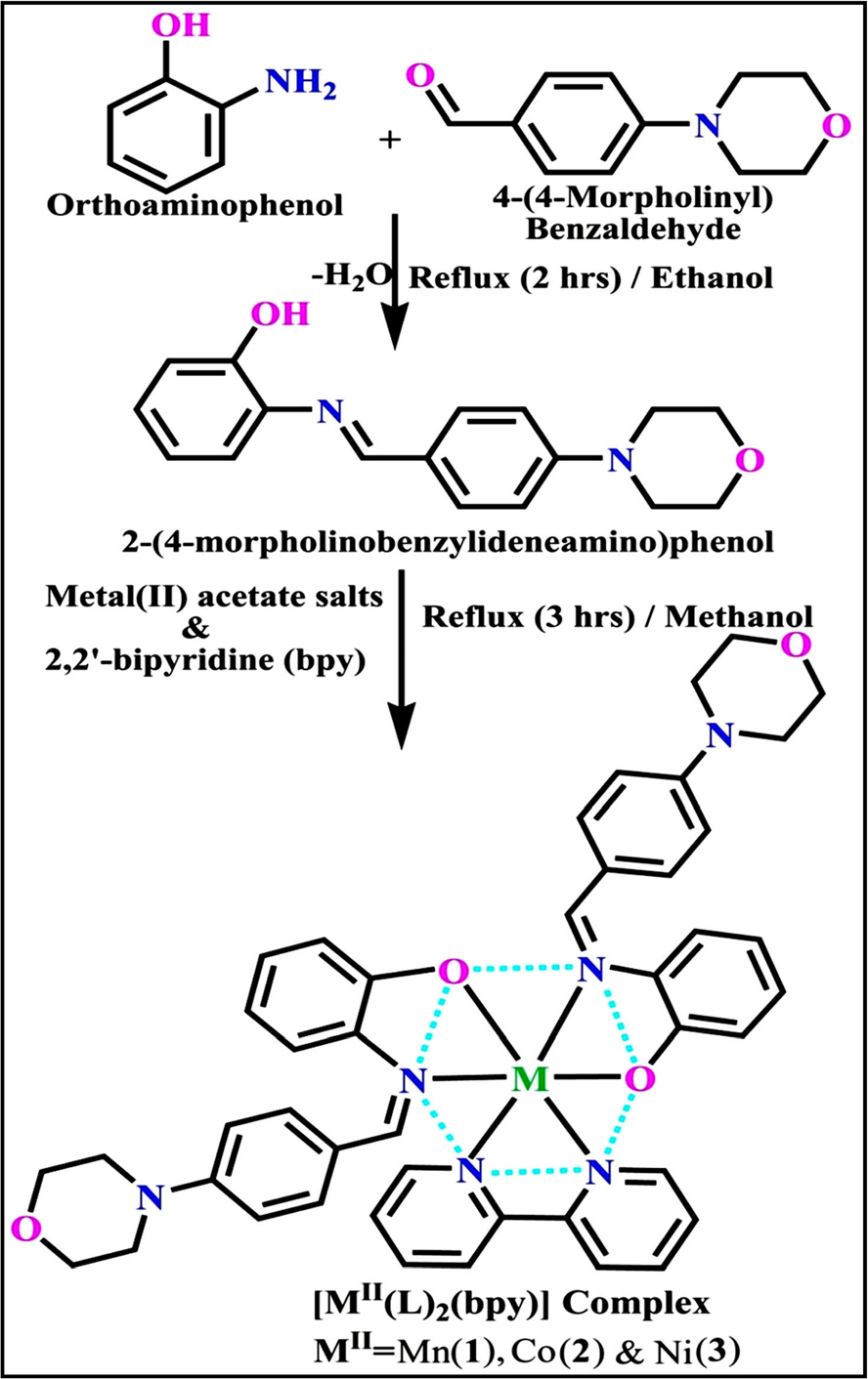
The proposed structure of complexes (1–3) [M^II^(L)_2_ (bpy)].

The evaluated analytical results, structural characteristics, and crystallographic data for ligand (**HL**) and its mixed ligand complexes (1–3) (Scheme 1) are presented in the ESI† data file (3a) (Fig. S1–S47 and Tables S1–S15).

### 3.2. DNA/BSA-binding properties

In general, it is recommended to restrict the development of tumor cells by preventing the reproduction of DNA that has been damaged or broken due to binding or cleavage mechanisms. This deals with the static mode of binding between test compounds and BSA.

#### 3.2.1. Analysis of DNA cleavage characteristics

**Fig. 1:**
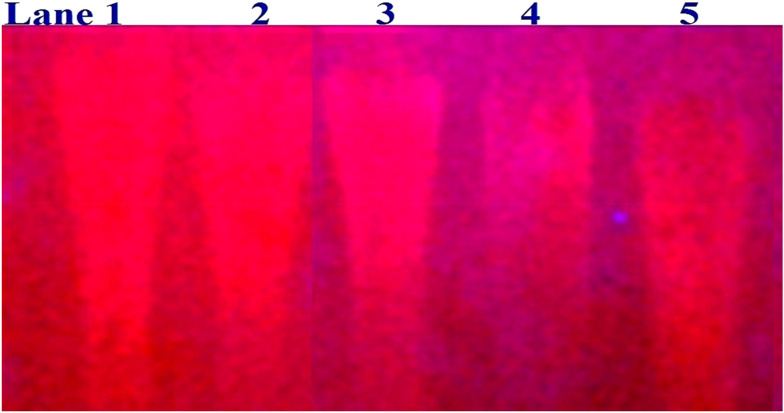
Ethidium bromide displacement assay: gel electrophoresis demonstrates the DNA cleavage property in the H_2_O_2_ environment for the following substances. Lane 1: DNA alone + H_2_O_2_; lane 2: ligand (**HL**) + DNA + H_2_O_2_; lane 3: complex (1) + DNA + H_2_O_2_; lane 4: complex (2) + DNA + H_2_O_2_; lane 5: complex (3) + DNA + H_2_O_2_.

**Fig. 2:**
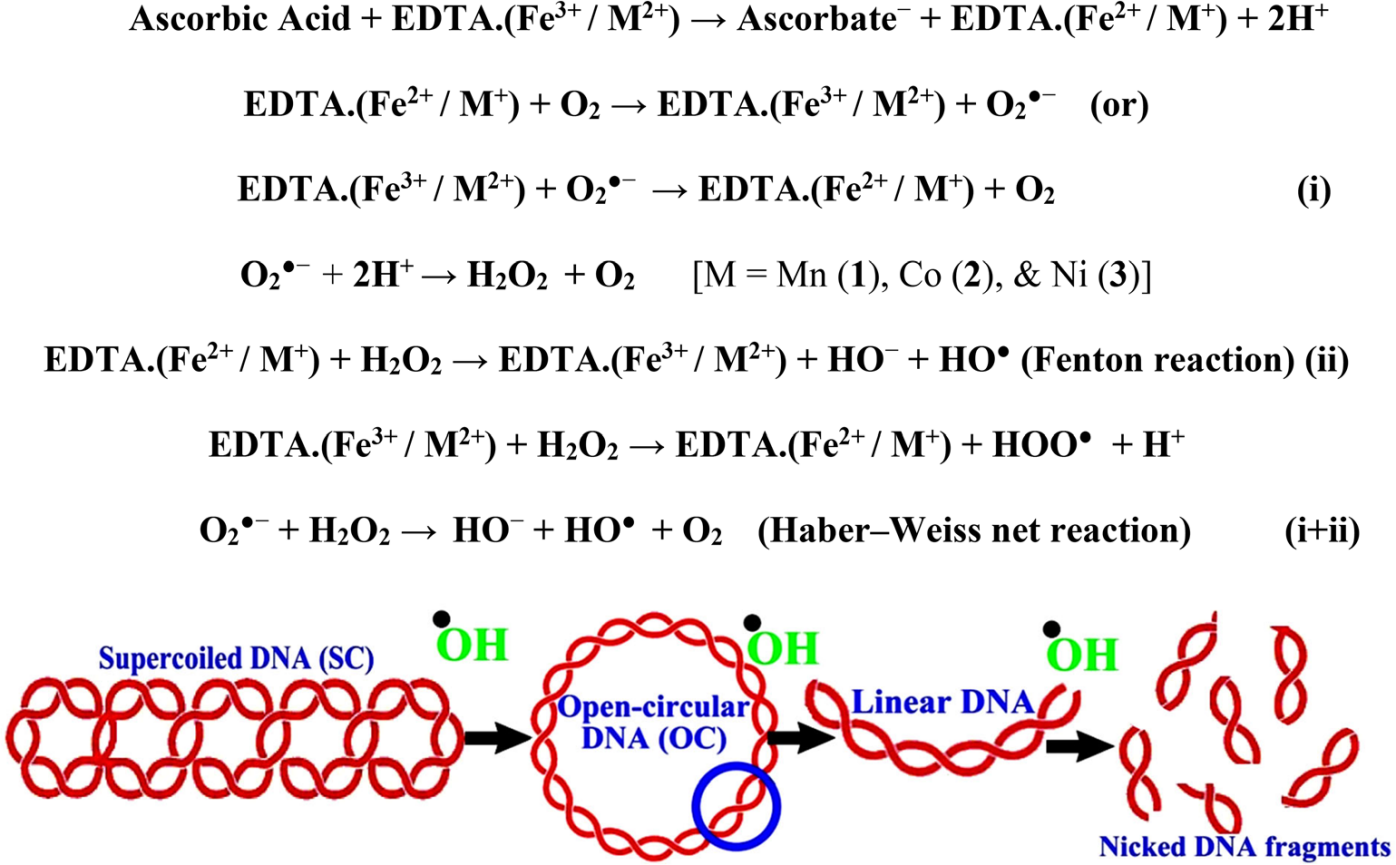
Fenton and Haber–Weiss mechanisms for DNA cleavage in the H_2_O_2_ environment.

In a cellular system, DNA base pairs can be broken by a variety of mechanisms, including errors in DNA replication, generation of a heteroduplex during homologous recombination, spontaneous deamination of cytosine, and base pairs broken by mutagens or ionizing radiation, which also lead to improper base pairing and mutations. Also, metal complexes have the capability of recognizing DNA mismatches and may become vital entities for investigation and possibly for clinical applications.^55^ Moreover, the cleavage of one or both DNA strands is a typical and essential process for maintaining cell viability: during DNA replication and transcription, topoisomerase enzymes correct topological issues, and various nucleases take part in repair mechanisms and DNA degradation, which is one of the distinguishing features of apoptotic programmed cell death. Similarly, several antitumor drugs have the potential to cleave DNA by inducing apoptosis, which eventually leads to cancer cell death. Conversely, molecules that can interact with DNA's major or minor grooves are known as groove-binding molecules, which disrupt and impair the function of the DNA double helix *via* various non-covalent interactions. The DNA nuclease properties of all samples were assessed in an H_2_O_2_ environment by the gel electrophoresis method. DNA cleavage was monitored during the conversion of supercoiled plasmids into linear and nicked DNA fragments. Also, the observed DNA nuclease efficacy for all complexes (1–3) was compared with the free ligand (**HL**) and CT-DNA alone. No substantial nuclease activity can be seen in the control (Fig. 1 and S13†) (lane 1; DNA + H_2_O_2_) even after a lot of time has passed, and free ligand (**HL**) (lane 2) was monitored as immobile in an H_2_O_2_ environment. Lane 4 shows that complex (2) demonstrates complete DNA cleavage. Similarly, lane 5 reveals that complex (3) undergoes partial DNA cleavage. Also, the performance of band reduction in the lanes was revealed in agarose gel (Fig. 1 and S13†), but lane 3 indicates that complex (1) shows no considerable cleavage efficiency among the series of complexes. Consequently, it is commonly acknowledged that ROS plays a dual physiological role in controlling a variety of illnesses as well as cellular homeostasis (self-regulating processes like thermoregulation, blood glucose regulation, calcium/potassium homeostasis, and osmoregulation).^56^ Numerous oxidases, peroxidases, lipoxygenases, dehydrogenases, cytochromes P450, and other enzymes have been demonstrated to be able to produce ROS. Additionally, it is widely known that the NADPH oxidase enzyme produces reactive oxygen species as part of its antibacterial effect on phagocytic cells. Nevertheless, these types of enzyme seem to be present in a variety of other cells and may have significant signalling pathway functions. When non-carcinogenic toxicity events occur, ROS has the ability to alter cell function as well as to affect the genesis of cancer at several levels. OH˙ can attack DNA, proteins, and lipids due to its high reactivity among ROS. Also, the hydroxyl radical is a key participant in free-radical-mediated hazardous reactions because of its great reactivity. Free radicals are essential in the redox regulation of many cell signalling pathways and proper cellular functions, and they are only generated in living systems; superoxide (O_2_˙^−^) was believed to be a typical cellular metabolite. It was then realized that more dangerous radicals could potentially be produced *via* the Haber–Weiss process. The combination of O_2_˙^−^ and H_2_O_2_ may produce a powerfully reactive OH˙ radical.^57^ As per the Fenton/Haber–Weiss mechanism, it is suggested that it is capable of vigorous nucleolytic cleavage by chemical substances in an oxidizing agent (H_2_O_2_) environment^58^ According to this mechanism, the complexes acted as excellent catalysts for the creation of diffusible ˙OH free radicals from hydrogen peroxide. Additionally, ˙OH free radicals abstract the H-atom from the sugar fragment of the DNA base pair to generate sugar radicals. Concerning the location of the hydrogen atom, it rapidly induces hydrolytic nuclease activity at the sugar–phosphate backbone.^59^ The rapid migration of DNA can lead to transformation of the open circular form into a linear form. Moreover, EDTA facilitates the generation of highly reactive diffusible OH˙ and anions *via* the Fenton or Haber–Weiss processes and prevents metal ions from interacting with DNA due to the generation of an EDTA–metal system. The diffusible hydroxyl free radicals also stimulate the abstraction of the H-atom from the sugar part of the DNA base pair to generate sugar radicals along with the formation of an adduct with nucleobases. Therefore, DNA cleavage occurs owing to the assault of a diffusible OH˙ on DNA base pairs in the presence of a metal complex environment. The complex serves as an effective catalyst for the production of OH˙ from hydrogen peroxide according to the Fenton mechanism.^60^ Also, Fe^3+^ (or M^2+^) is regenerated by an ascorbate anion (dehydroascorbic acid) into active Fe^2+^ (or M^+^), which functions as a reducing agent. Ascorbic acid and ascorbate are both already present in the human body and interconvert with each other. Also, ascorbate is the predominant form at physiological pH. Therefore, Fe^2+^/ascorbate^−^ (or M^+^/ascorbate^−^) generated hydroxyl radicals are efficiently involved in the DNA damaging process. The following descriptions of the general mechanisms of the metal–EDTA/H_2_O_2_ system are shown in Fig. 2 and S14.† Generally, if the metal complexes have a high efficiency of H-abstraction from the sugar fragment, it facilitates the DNA damaging process. On the other hand, if metal complexes have weak hydrogen abstraction, they have no substantial nuclease activity. It is finally concluded that complex (2) revealed complete DNA cleavage in this case, which may occur due to strong H-abstraction ability.

#### 3.2.2. Assessment of DNA binding properties using UV-vis absorption titration

**Fig. 3:**
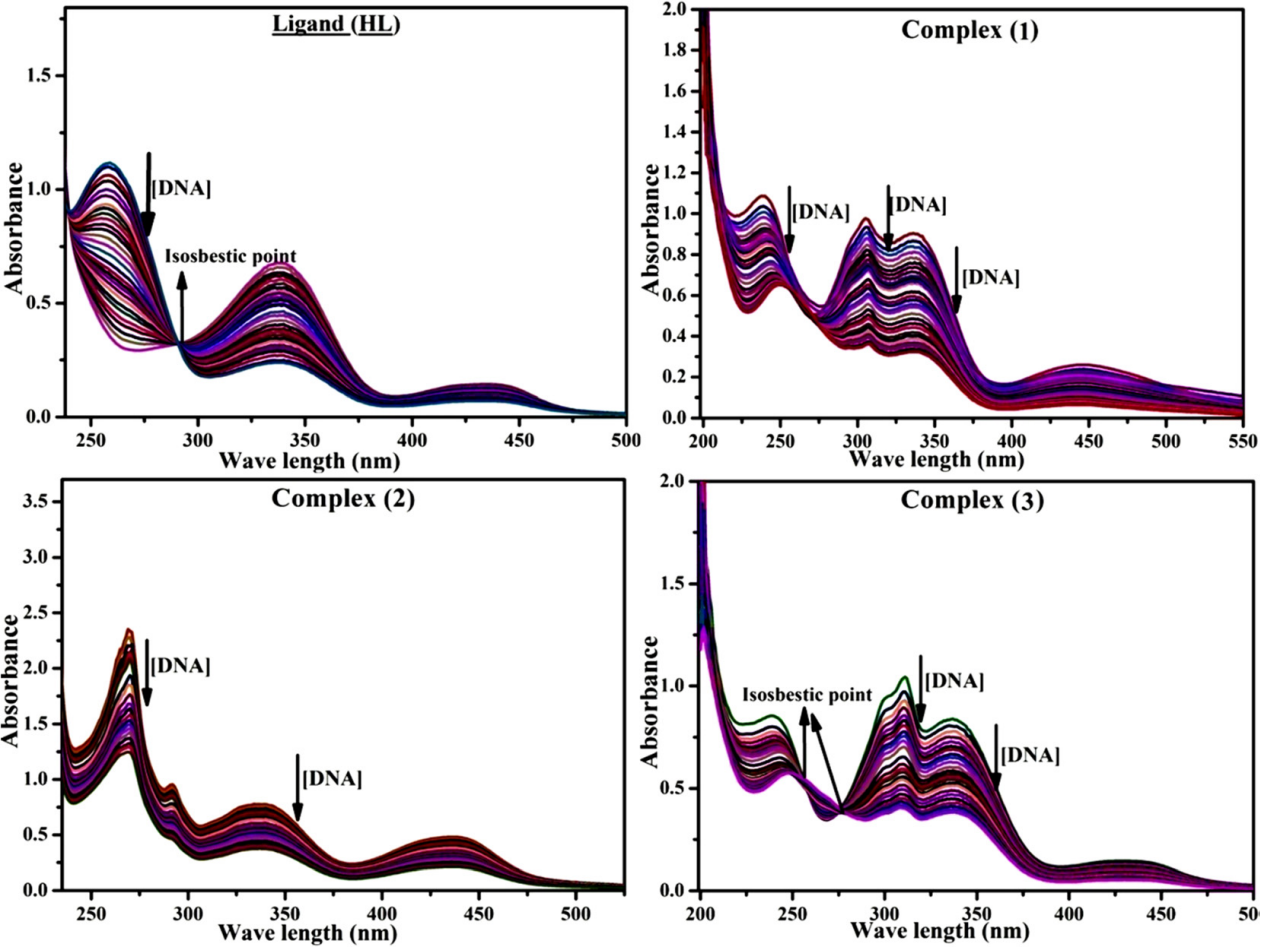
Increasing concentrations of CT-DNA were present while the ligand (**HL**) and mixed ligand complexes (1–3) were measured for their absorption spectra in a Tris–HCl buffer solution at room temperature. Arrows depict the changes in absorbance that occur as CT-DNA concentration is increased, and another arrow with isosbestic points denotes that equilibrium between DNA and complexes has been achieved.

Most medications involve intercalation and groove binding *via* GC (guanine/cytosine)-rich and AT (adenine/thymine)-rich domains, respectively. The interactions were determined experimentally by electronic absorption spectrum titration. Moreover, intercalating binding is indicated by bathochromic and hypochromic shifts in the absorption spectra, whereas groove binding of the complexes with DNA is indicated by hyperchromic shifts in the titration curve.^61^ Generally, four kinds of non-covalent engagements that can be absolutely critical in the interaction of substances with DNA are most frequently illustrated in the literature: (i) involves a negatively charged phosphate fragment as a result of electrostatic interaction; (ii) influences weak van der Waals force attraction/H-bonding; (iii) interaction of a functional moiety with the grooves (major/minor) of the double-stranded DNA as a result of a molecule sticking due to general attraction, or as a result of water or H-bonding expulsion, *etc.*; (iv) the stacked base pairs of natural DNA are intercalated by hydrophobic forces. Nevertheless, substances are engaged in the reaction because of quinine's preferred N-7 position and adenine's N-3 location in DNA. The DNA base pairing may also be prevented due to miscoding. All complexes (1–3), including free ligand (**HL**), were measured both when DNA was present and when it was absent using ultraviolet–visible spectrophotometric absorption titrations in buffer solution with a pH of 7.4 at 25 °C (Fig. 3). The results are also included in Table 1. In this case, all substances were exposed to two prominent electronic absorption bands of about 260 nm and 335–343 nm, consequent to the π–π* transitions of the phenyl chromophore and MLCT, respectively. While the amount of DNA in each compound rises, the interaction of the chemical substance with DNA base pairs generates noticeable alterations in the strength and wavelength of the intra-ligand charge transfer bands. The hypochromic shift of all compounds was observed in the range of 36.16–43.46% with 4–5 nm red shifts, which occurred due to a diminishing in the π–π* transition energy and the half-packed electrons of bonding orbitals. In contrast, electrostatic interaction would be possible if the complex–DNA adduct exhibited hyperchromism with a hypsochromic shift.^61^ Using Wolfe–Shimmer eqn (1) and (2), Benesi–Hildebrand eqn (3) and (4) and Sakthi–Krause eqn (5) and (6), the observed overall *K*_b_ values for all samples were in the following sequence: (2) > (3) > (1) > (**HL**). Moreover, the observed 
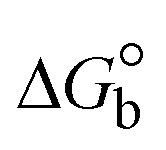
 values in all cases were in the range of −19.91 to −24.83 kJ mol^−1^ (Table 1), which also indicates that the compounds spontaneously intercalate to DNA. However, complex (2) exhibited excellent binding potency compared to others. It is concluded that the co-planarity of the morpholine-linked ligand and complexation of the 2,2′-bipyridine aromatic system with the metal centre promote the ability of the complex to infiltrate DNA base pairs smoothly. Large aromatic systems may also assist the complex to deeply penetrate the core of the phosphate backbone, and those substances may permit the complex to freely penetrate deep into the DNA base pairs. In addition, the observed isosbestic points are found at 285 nm for free ligand and 256, 276 nm for complex (3), respectively. This also suggests that DNA and complexes establish a dynamic equilibrium and it can be further concluded that complexes (1–3) spontaneously intercalate into DNA. The Wolfe–Shimmer eqn (1) and (2),^62^ Benesi–Hildebrand eqn (3) and (4),^63^ and Sakthi–Krause eqn (5) and (6) were applied to evaluate the *K*_b_ results for all samples, which were obtained by modification of the Lineweaver–Burk and Stern–Volmer equations (Table 1).

**Table 1:** UV-vis spectral DNA binding parameters for all of the compounds

Compounds	*λ*_max_ (nm)	Δ*λ* nm (% H)	*K*_b_ × 10^4^ M^−1^			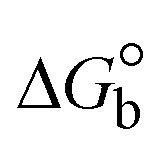 (kJ mol^−1^)		
	Free (bound)		WS-I (WS-II)	BH-I (BH-II)	SK-I (SK-II)	WS-I (WS-II)	BH-I (BH-II)	SK-I (SK-II)
(**HL**)	336 (340)	04 (37.13)	1.5169 (1.5480)	0.6000 (1.9515)	0.8775 (1.7216)	−23.85 (−23.90)	−21.55 (−24.48)	−22.50 (−24.18)
(1)	335 (339)	04 (40.61)	1.8195 (2.0826)	0.9942 (2.1067)	1.1049 (2.0389)	−24.30 (−24.64)	−22.80 (−24.67)	−23.07 (−24.58)
(2)	336 (340)	04 (36.16)	1.8806 (2.1379)	1.0468 (2.1524)	1.4965 (2.2526)	−24.38 (−24.70)	−22.93 (−24.72)	−23.82 (−24.83)
(3)	335 (340)	05 (43.46)	1.8207 (2.1275)	1.8212 (2.0938)	1.3001 (2.0417)	−24.30 (−24.69)	−24.30 (−24.65)	−23.47 (−24.59)

Hypochromism 
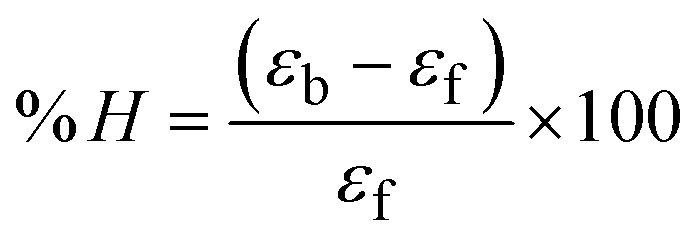
; *ε*_f_ and *ε*_b_ denote the extinction coefficient of the substance alone and the extinction coefficient of the substance fully interacted with deoxyribonucleic acid; WS represents Wolfe–Shimmer; BH denotes Benesi–Hildebrand methods (BH-I & II); SK represents Sakthi–Krause methods (SK-I & II); 
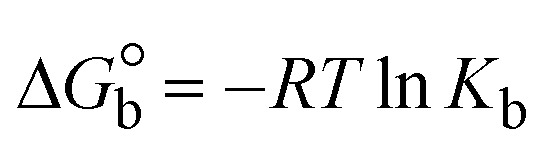
, *K*_b_ = intrinsic DNA binding constant evaluated from the electronic absorption spectral titration, *R* is the universal gas constant = 1.987 cal K^−1^ mol^−1^ or 8.314 J K^−1^ mol^−1^, *T* = 298 K; error limit ± 2.5% (*P* < 0.025).1
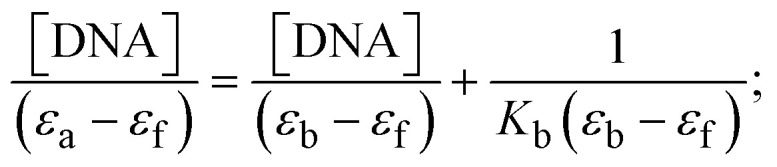
2
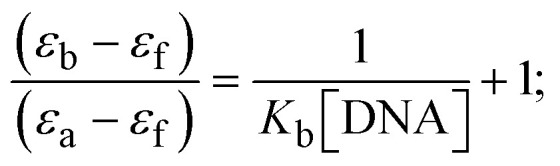
3
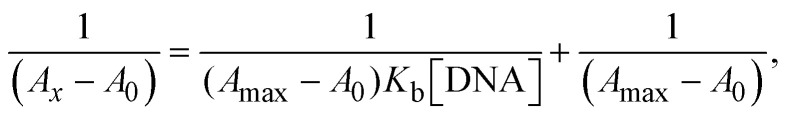
where 
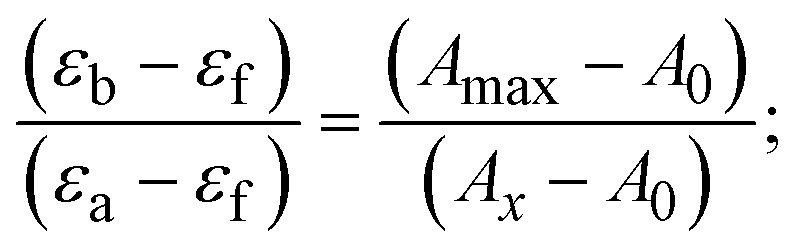
4
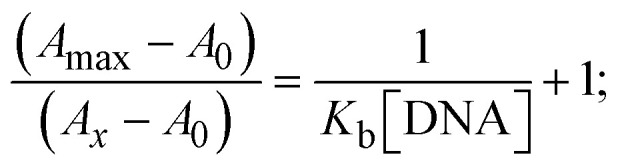
5
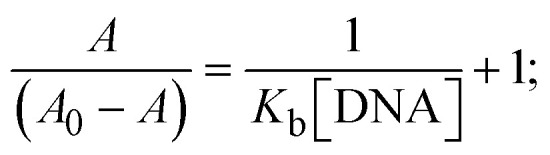
6
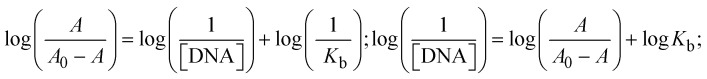
7
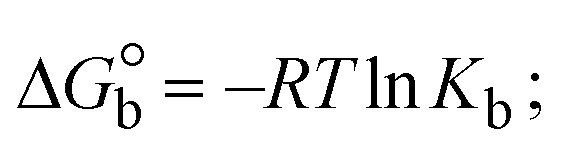
8
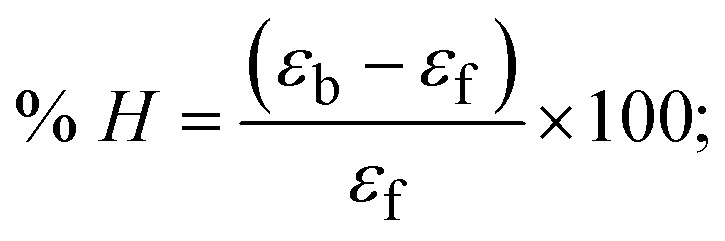
where *ε*_a_ represents the apparent absorption coefficient value for the MLCT band at a specific concentration of deoxyribonucleic acid and is evaluated from Abs/[complex]. *ε*_f_ and *ε*_b_ are absorption coefficient values for the chemical substance alone and fully interacted with deoxyribonucleic acid, respectively. Δ*A*_max_ = (*A*_max_ − *A*_0_); Δ*A* = (*A*_*x*_ − *A*_0_), where *A*_0_, *A*_*x*_ and *A*_max_ denote the absorbance of the chemical substance alone, the intermediate form, and the completely interacted form with deoxyribonucleic acid, respectively.

The *K*_b_ values were measured using the Wolfe–Shimmer eqn (1) and (2) from the linear regression plots of [DNA]/(*ε*_a_ − *ε*_f_) *vs.* [DNA] M^−1^ for method I and (*ε*_b_ − *ε*_f_)/(*ε*_a_ − *ε*_f_) *vs.* 1/[DNA] M^−1^ for method II, respectively (Fig. S15†). The Benesi–Hildebrand binding constant (*K*_b_) values were measured using eqn (3) and (4) from the linear regression plots of [1/(*A*_*x*_ − A_0_)] *vs.* {1/[DNA]} M^−1^ for method I and [(*A*_max_ − *A*_0_)/(*A*_*x*_ − *A*_0_)] *vs.* {1/[DNA]} M^−1^ for method II, respectively (Fig. S16†). The *K*_b_ values were estimated using Sakthi–Krause eqn (5) and (6) from the linear regression plots of [*A*/(*A*_0_ − *A*)] *vs.* {1/[DNA]} M^−1^ for method I (Fig. S17†) and {1/[DNA]} *vs.* log[*A*/(*A*_0_ − *A*)] M^−1^ for method II (Fig. S18†). In addition, *A*_0_ and *A* represent the absorbance intensity values in the absence and presence of [DNA], respectively. The van't Hoff eqn (7) was utilized to obtain the 
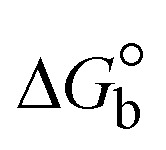
 values for DNA interaction, and eqn (8) was utilized to measure the percentage of chromaticity for all substances. Nevertheless, the findings observed with the Sakthi–Krause methods were in good correlation with the Wolfe–Shimmer and Benesi–Hildebrand approaches. Complex (2) had the highest DNA binding efficacy among all the binding results. The DNA cleavage, emission, hydrodynamic, and CV measurements all support the preceding observations.

#### 3.2.3. Assessment of thermal denaturation characteristics

The DNA double helix is primarily sustained by base-pair stacking interactions and hydrogen bonds between purines and pyrimidines on opposing strands. The stabilizing bonds that keep the DNA double helix together are broken when the temperature rises, causing both strands to separate. This process is known as the thermal denaturation of DNA. The melting temperature (*T*_m_) of DNA is the temperature at which one half of the double helix denatures into a single strand. The cooperative unwinding is also denoted helix-coil or melting transition (temperature of midtransition), which is further measured from the derivative melting curve between temperature (°C) and *dA*_260_/*dT* (Fig. S19 and S19a†). Generally, *T*_m_ is measured from the absorption values at a wavelength of 260 nm between 50 °C and 100 °C. Moreover, DNA denaturation is caused not only by heat but also by organic solvents like formamide and DMSO, raising the pH of the solution, lowering the concentration of salt, *etc.*^64^ DNA denaturation plays a role as a root cause of a number of chronic diseases, hereditary disorders, and a reduction in the ability of DNA repair to work properly. Thermal denaturation experiments are frequently used to determine the stability of a compound. When a sample is heated, the absorbance characteristics frequently change, reflecting a conformational change in the molecules in solution. The stability of the DNA secondary structure may be determined by such an experiment. Proteins typically undergo irreversible denaturation. But nucleic acids frequently undergo renaturation when the sample is cooled. Most often, thermal denaturation tests can be performed with an absorption spectrophotometer by measuring the absorbance at 260 nm as a function of temperature.^65,66^ Also, the biothermodynamic properties were further studied to determine the ability to stabilize the double-stranded DNA and this study offers details on the structural alterations, the degree of the DNA–compound system, the external binding-mediated neutralization of the phosphate charges on DNA, and the stacking interactions, all of which work together to raise the melting point of DNA.^67^ Moreover, small molecules are involved in the reaction due to DNA's preferred N-7 site for guanine and N-3 for adenine. Therefore, it is possible to block the DNA double helix, which causes miscoding of DNA. In this case, it is observed that complex–DNA adducts have higher melting temperature than free DNA. Complex-bound DNA is more challenging to melt than DNA alone because it is involved in powerful intercalation binding with DNA. The van't Hoff eqn (9), Gibbs–Helmholtz eqn (10) and (10a), van't Hoff plot eqn (10b) and theoretical melting temperature eqn (10c) for various nucleotides were supportive in evaluating the biothermodynamic parameters, which are listed in Table 2. Also, this technique offers crucial data on binding constants and associated Δ*G*°, Δ*S*° and Δ*H*° findings for compound–DNA systems. Generally, the thermal denaturation temperature of DNA is typically only slightly affected by groove binding or electrostatic binding along the phosphate backbone, but intercalation results in a considerable increase owing to the stabilization of the Watson–Crick base-paired duplex. Thus, the technique is also supportive of both detecting binding constants and relative binding strengths. The transition midpoint of this curve yields the value of *T*_m_ for Ct-DNA alone, which was measured at 68 ± 2 °C and the observed *T*_m_ values of the DNA–substance adduct were in the following sequence: (2) 78 °C > (3) 77 °C > (1) 76 °C > (**HL**) 74 °C and the value of Δ*T*_m_: (**EB**) (13 °C) > (2) 10 °C > (3) 9 °C > (1) 8 °C > (**HL**) 6 °C. In general, Δ*T*_m_ > 8 °C denotes an intercalative mode of binding, while Δ*T*_m_ < 8 °C represents the groove and/or electrostatic binding mode(s) in the DNA–compound adduct.^66,67^ In this case, all observed values were greater than 8 °C except for the ligand (**HL**) (Fig. S19, S19a† and Table 2). Also, the binding process is mostly enthalpy-driven and involves hydrogen bonding, as indicated by the negative value of Δ*H*°. Van der Waals interaction may have played a role in the creation of the complex, as shown by the negative value of Δ*S*°.^68^ As per Ross and colleagues, the findings for Δ*H*° and Δ*S*° can alternatively be derived in the following favourable sequence. If Δ*H*° > 0 and Δ*S*° > 0, intercalation is attributed to hydrophobic forces of attraction. If Δ*H*° < 0 and Δ*S*° < 0, weak van der Waals forces of attraction and H-bonding interactions are involved. On the other hand, Δ*H*° < 0 (or Δ*H*° ≈ 0) and Δ*S*° > 0 indicates that electrostatic modes of binding are possible between DNA and compounds.^69^ The measured values for all the samples were exposed to the favourable sequence Δ*H*° < 0 and Δ*S*° < 0, which is assumed to be due to weak van der Waals forces of attraction and H-bonding between DNA and chemical substances. However, they lose the ability to rotate and translate, interfere with counter ions and hydrophobic forces in compound–DNA adducts, which may result in exothermically active negative signals of Δ*S*° and Δ*H*°. Furthermore, it is widely acknowledged that hydration and the generation of the compound–DNA adduct system *via* the counter-ion liberating mechanism are highly dependent on hydrophobic forces of attraction. As a result, higher negative results of Δ*H*° and Δ*S*° for all substances that interacted with DNA were observed in the experiments.^70^

**Table 2:** UV-vis absorption spectra with biothermodynamic properties for the binding of the ligand (**HL**) and its complexes (1–3) to CT-DNA

Compounds	*T* (°C) (K) 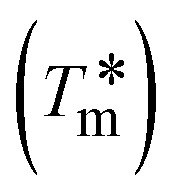	Binding constants *K*_r_ @ 298 K (M^−1^) & *K*_m_ @ *T*_m_ K (M^−1^)	Δ*H*° (kcal mol^−1^)	Δ*S*° (cal mol^−1^)	Δ*G*° (kcal mol^−1^)
(**HL**)	10 (283)	5.2575 × 10^4^	−3.2141	−5.9041	−6.1124
	25 (298)	1.5169 × 10^4^			−5.7004
	40 (313)	6.0674 × 10^3^			−5.4174
	74* (347)	1.4625 × 10^3^	−9.8084	−13.7852	−5.0249
(1)	10 (283)	7.2350 × 10^4^	−3.3331	−6.1793	−6.2919
	25(298)	1.8195 × 10^4^			−5.8081
	40 (313)	7.7321 × 10^3^			−5.5682
	76* (349)	2.2010 × 10^3^	−8.5588	−9.2304	−5.3376
(2)	10 (283)	9.8820 × 10^4^	−3.7445	−7.4978	−6.4673
	25 (298)	1.8806 × 10^4^			−5.8277
	40 (313)	8.0433 × 10^3^			−5.59278
	78* (351)	2.3442 × 10^3^	−8.1656	−7.8457	−5.4118
(3)	10 (283)	9.1140 × 10^4^	−3.6540	−7.2160	−6.4218
	25 (298)	1.8207 × 10^4^			−5.8085
	40 (313)	7.8797 × 10^3^			−5.5798
	77* (350)	2.2351 × 10^3^	−8.3598	−8.5613	−5.3633

*T*_m_ is the melting temperature of free CT-DNA = 68 °C (341 K); (**HL**) = 74 °C (347 K); (1) = 76 °C (349 K); (2) = 78 °C (351 K); (3) = 77 °C (350 K). (0 °C = 273.15 K); Δ*T*_m_ denotes the melting temperature changes between DNA–compound adducts and CT-DNA alone: (**HL**) = 6 °C (279 K); (1) = 8 °C (281 K); (2) = 10 °C (283 K); (3) = 9 °C (282 K). 
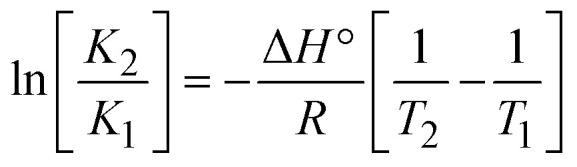
; enthalpy change 
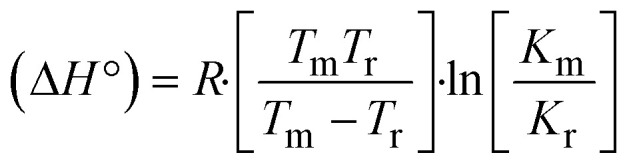
, *T*_1_ = *T*_r_ → 298 K, *T*_2_ = *T*_m_ → DNA melting temperature of compounds, universal gas constant (*R*) = 1.987 cal K^−1^ mol^−1^ or 8.314 J K^−1^ mol^−1^; entropy change (Δ*S*°) = 
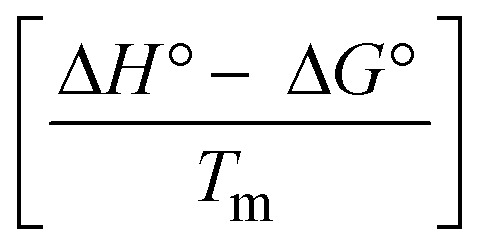
.9
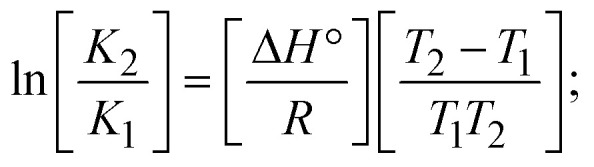

10 Gibb's free energy, Δ*G*° = −*R*·*T*_m_·ln *K*_m_;

10a Δ*G*° = Δ*H*° – *T*_m_Δ*S*°;

where *K*_1_ represents the binding constant value at 298 K (*T*_r_), the binding constant *K*_2_ (*K*_m_), which indicates the temperature at which substances melt their DNA (*T*_m_ K).The van't Hoff plot eqn (10b) is obtained by comparing eqn (10) and (10a),10b
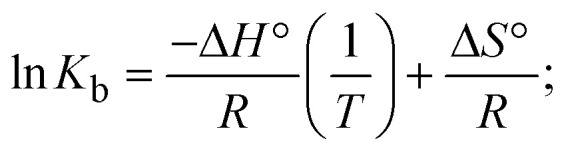
where Δ*H*°/*R* > 0, slope (*m*) = −Δ*H*°/*R* < 0 → endothermically favorable, while Δ*H*°/*R* < 0, slope (*m*) = −Δ*H*°/*R* > 0 → exothermically favorable. Theoretical melting temperature for various nucleotides,

10c *T*_m_(°C) = [7.35 × *E*] + [17.34 × ln(Len)] + [4.96 × ln(Na^+^)] + [0.89 × ln(DNA)] − 25.42;

where, *E* → DNA strength parameter per base = (cumulative DNA strength/length of the DNA sequence), ln(Len) → logarithm of the length of the DNA sequence, ln(Na^+^) → logarithm of [Na^+^] concentration of solution (M), ln(DNA) → logarithm of total nucleotide strand concentration. All measurements of *T*_m_ were repeated three times and the data presented are the average values with lower than 5% (*P* < 0.05).

According to the Ross and Subramanian mechanism for protein/DNA–complex interactions, it is obviously revealed that the complexation of the metal center with the morpholine-fused primary aromatic and 2,2′-bipyridine secondary aromatic planar systems stimulates the silky penetration of the complex which is sandwiched within DNA base pairs. The stability of the complex is optimized by π–π stacking interactions, including a number of non-covalent molecular interactions like dipole–dipole interaction, weak van der Waals forces of attraction, formation of hydrogen bonding, electrostatic forces of attraction, ionic interactions between positively charged groups of the complex and DNA phosphate groups; reduction of Coulombic repulsion between the DNA phosphate groups is associated with the increasing distance between the helix unwinding bases, *etc.* In general, cationic species are more effective DNA intercalators due to their improved initial interactions with the negatively charged DNA sugar–phosphate backbone as well as the fact that intercalation releases counter ions (Na^+^) associated with phosphate groups, which is known as the polyelectrolyte effect. This is a crucial driving force for intercalation due to the reduction in repulsive interactions between the closely spaced charged counter ions. In actuality, the majority of intercalating molecules are either positively charged or have basic groups that can undergo protonation under physiological conditions. According to Chaires' research, the thermodynamic properties of drug–DNA bindings have a substantial influence on bimolecular complex formation. Altering the DNA configurations resulted in a significant decrease in the binding enthalpies of all intercalators, with the exception of actinomycin. While the cationic molecule binds with the DNA base pair, it exchanges the reduced counter ions from the dense interior surface layer and defuses the exterior surface layer of neighbouring DNA. It also reduces the regional charge density. Another type of molecular interaction is the counter-ion liberating mechanism in DNA-binding complexes. As a result of the cumulative complex–DNA interaction, the entropy and enthalpy change significantly decrease.^71^ Moreover, from the van't Hoff plot of ln *K*_b_*vs.* 1/*T* (K^−1^), it has been revealed that the negative Δ*H*°, Δ*S*° and Δ*G*° values of the complex–DNA adduct can be attributed to complexes (1–3) spontaneously intercalating DNA with exothermic and spontaneous processes. Thus, these large negative enthalpy and entropy changes are properties of the interaction through intercalation, which is further stabilized by other non-covalent interactions in the double helix of DNA (Fig. S20†). Additionally, the intercalation of the compound between DNA bases causes a large negative entropy change, which is attributed to the loss of translational and rotational degrees of freedom. As a result, it is concluded that H-bonds and van der Waals interactions, which can occur both electrostatically and through intercalation, significantly aided the binding of the complex to DNA and overall stability.

#### 3.2.4. Assessment of DNA binding affinity using viscometric techniques

The viscometric technique is one of the most effective and dependable methods for determining binding strength and the mechanism of interaction between a chemical substance and DNA. Generally, when the major/minor groove binding, electrostatic, partial, and non-classical interaction modes are involved between chemical substances and DNA base pairs, the final DNA relative viscosity remains unchanged or undergoes a very negligible change due to reducing the contour length of DNA. When the intercalation binding modes are involved between a chemical substance and DNA base pairs *via* π–π stacking interactions and hydrophobic forces, an increase in relative viscosity is noted due to a rise in the contour length of DNA. Moreover, for the purpose of observing alterations in the CT-DNA helical structure, the viscosity findings for CT-DNA, various concentrations of chemical substances present, and EtBr were recorded. It was also noted that the absolute viscosity rose consistently along with the incremental concentration of each substance at the fixed DNA concentration. As a result of the strong binding mode of intercalation, the contour length of the double-helix DNA rises.^72^ The obtained results are also compared with those of the classical intercalator (**EB**). The affinity interaction and their slope values were observed from the relative specific viscosity (*η*/*η*_0_)^1/3^ plotted as a straight line contrasting [compound]/[DNA] and absolute specific viscosity of DNA in the presence or absence of chemical substances was evaluated using eqn (11) (Table 3). In the experiment, it was clearly noted that the slope values for all samples increased due to the rising binding affinity. The evaluated slopes were in the following sequence: (**EB**) 1.215 > (2) 0.860 > (3) 0.801 > (1) 0.662 > (**HL**) 0.490 (Fig. S21† and Table 3). However, complex (2) exhibited superior binding affinity to the others and was substantially smaller than **EB**. Due to the existence of 2,2′-bipyridine and the morpholine-fused aromatic planar systems, compounds can interact with DNA robustly *via* intercalation. The outcomes agreed with the observed outcomes of electronic absorption spectral characteristics.

**Table 3:** Relative specific viscosity *versus* [complex]/[DNA]

Compounds	Binding ratio (*R*) = [complex]/[DNA]						
	0.2	0.4	0.6	0.8	1.0		
	Relative specific viscosity (*η*/*η*_0_)^1/3^					Slope	*R* ^2^
**EB** (control)	1.01	1.35	1.63	1.82	1.99	1.215	0.9738
(**HL**)	0.61	0.67	0.75	0.85	1.01	0.490	0.9600
(1)	0.66	0.78	0.88	1.02	1.12	0.662	0.9813
(2)	0.77	0.88	1.05	1.18	1.48	0.860	0.9620
(3)	0.71	0.86	0.92	1.11	1.31	0.801	0.9809



11

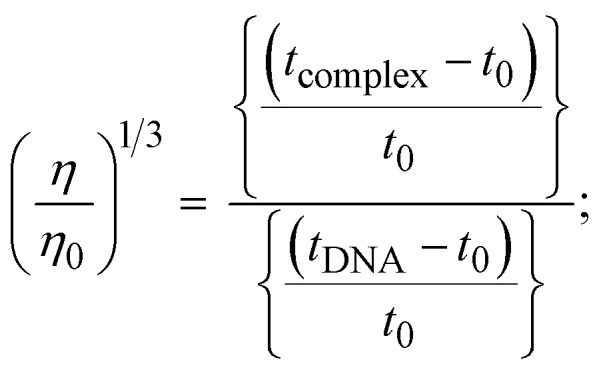

where *η* and *η*_0_ represent the specific viscosity of DNA in the presence of the complex and the specific viscosity of DNA alone, *t*_0_, *t*_DNA_ and *t*_complex_ represent the average flow time of the Tris–HCl buffer solution, the average flow time of the DNA alone solution, and the average flow time of DNA interacted with the samples, respectively. Error limit ± 2.5% (*P* < 0.025).

#### 3.2.5. Assessment of DNA/BSA binding characteristics using emission titration

Fluorescence emission spectral titration is an efficient approach to evaluating the binding properties of DNA/BSA biomolecules. In general, the fluorescence of **EB** is quite weak in aqueous solution, but when it is bound to DNA, the fluorescence intensity rises. However, it is a well-known imperative intercalator and is more supportive in distinguishing the binding strength of non-fluorescence test substances. Also, the fluorescence emission spectra of the **EB**–DNA adduct were examined at 610 nm in the absence and presence of rising quantities of each test compound. When the complex concentration (0–240 μM) rises, the fluorescence intensity of the **EB**-bound DNA complex diminishes owing to the displacement of **EB** from CT-DNA. A notable reduction in the fluorescence emission intensity at 610 nm is observed (Fig. S26† and Table 4). The photoelectron shift from DNA's guanine base to the excited states may be the cause of the frequency quenching in the emission of the test substance by DNA. Additionally, after each compound was added to **EB**, no additional peaks were noted, which shows that **EB** did not cause any quenching of its free fluorescence emission and proves that the compounds did not interact with **EB**. The intensity of the band significantly decreased as increasing amounts of each test substance were added to the fixed concentration of the **EB**–DNA adduct, demonstrating the ability of the investigated compounds to displace bound **EB** from DNA. Therefore, the competitive binding experiment can make use of **EB** as a fluorescent probe. Also, the addition of each molecule results in a diminution in the relative emission intensity of **EB**–DNA, which reveals that complex (2) demonstrates maximum efficiency, which is consistent with their binding capacities. A reasonable quenching in fluorescence intensity showed that complexes could connect with CT-DNA through intercalation and compete with **EB** for binding.^73^ This is further evidence that complexes (1–3) strongly bind intercalatively with DNA. The observed results agree well with the data from electronic absorption spectral measurements. Additionally, the experiment was extended to examine the binding properties between the test substance and BSA. BSA mostly contains three amino acid residues (tyrosine, tryptophan, and phenylalanine), which give it its inherent fluorescence. There are two tryptophan residues in BSA: Trp-134 is located in the IB subdomain, which is revealed to have a hydrophilic environment, and Trp-214 is located in the IIA subdomain. These two residues also have minimal quenching effects. Tryptophan in BSA fluoresces mostly because of a residue that is trapped inside a hydrophobic cavity. Therefore, it is crucial to model potential binding interactions with the metal complexes. Additionally, they exhibit tryptophan fluorescence at 278 nm for excitation and 350 nm for maximal emission. The fluorescence intensity also reduces while the test substances (1–3) are steadily blended with the BSA solution, proving that complexes (1–3) interact with BSA *via* altering the protein's secondary structure, which also leads to an alteration in the tryptophan environment of BSA (Fig. S27†). Furthermore, BSA structural similarities share 76% sequence identity with human serum albumin (HSA), the most prevalent protein in blood plasma that transports ions and proteins to cells and tissues. HSA is able to readily crystallize under the trivalent cations, but BSA has no ready crystallizing property. However, they consist of parallel physicochemical properties to each other.^74,75^ Additionally, the Stern–Volmer eqn (12) and (13) were employed to analyze the data (Fig. S28† and Table 4). Additionally, the *k*_q_ values for DNA and BSA binding were acquired in the range of 1.1636–2.8863 × 10^12^ and 2.6390–7.0774 × 10^12^ mol^−1^ s^−1^, respectively. They are also much greater than the collision quenching constant value (2.0 × 10^10^ mol^−1^ s^−1^). Therefore, it is assumed that the static quenching process was brought on by adduct construction between the test compounds and BSA rather than a dynamic collision. However, fluorescence spectroscopy is generally plagued by the inner filter effect (IFE), disturbing spectral analysis. The energizing ray is attenuated due to the highly concentrated solution sample. As a result, strong fluorescence is only seen on surfaces facing the excitation beam. The fluorescence intensity is reduced as a result of an inner filter effect generated by absorption of the excitation/emission wavelengths by some chemicals in the UV region. The results of the absorption wavelengths of all compounds in the range 335–336 nm, and the BSA excitation and emission wavelengths of 278 nm and 350 nm, respectively, were monitored to assess the effect of IFE in this approach, and neither of them responded to the IFE, as evident from their extremely low values of absorbance. However, in order to measure the biomolecule quenching constants using the Stern–Volmer equations, the inner filter effect must be taken into account. Eqn (12) was employed to resolve IFE during this experiment.^76^ The fluorescence emission intensities of ethidium bromide interacted with DNA at 610 nm and those of BSA at 350 nm and exhibited a distinctly reducing movement with increasing concentrations of the test compounds when the IFE was resolved, indicating that after being replaced with the substances, a few ethidium bromide molecules were released into solution, which caused the fluorescence of ethidium bromide to be quenched. Additionally, no emission spectrum shifting was seen following the BSA–complex adduct, indicating that ground-state BSA–compound systems formed as a result of a static quenching mechanism (Fig. S27†). Hence, IFE was resolved by observing the emission spectral changes of **EB**–DNA with the incremental concentrations of all test compounds (30, 60, 90, 120, 150, 180, 210, 240 μM) in Tris–HCl buffer solution (pH = 7.2). Also, the emission spectral alterations of BSA were evaluated with incremental concentrations of all test compounds from 2.5 μM to 25 μM in Tris–HCl buffer solution (pH = 7.2). In all cases, the observed *R*^2^ values for the linear plots of *F*_0_/*F vs.* [*Q*] and log(*F*_0_ − *F*)/*F vs.* log[*Q*] by Stern–Volmer (SV) methods I and II were almost 1, which is also a significant factor for measuring the impact of the inner filter effect. Moreover, it was observed that BSA might interact with complexes and that the polarity of BSA's fluorescence did not vary noticeably with complex titration. These findings, which were in agreement with the UV-vis spectral data, can be interpreted as the intercalation mode of the complexes between DNA base pairs and bovine serum albumin. The following Stern–Volmer eqn (13)–(15) were employed to determine the *K*_SV_, *K*_q_, and *n* values. The *K*_SV_ values were measured from the linear regression plot of *F*_0_/*F vs.* [*Q*] by SV method I (Fig. S22 and S28†). Eqn (14) was employed to evaluate the *n* and *K*_ass_ values.^77^ Similarly, *K*_app_ (apparent binding constant) values for all samples were estimated with eqn (15) (Table 4).

**Table 4:** Determination of *K*_b_ and *n* values for all substances with **EB**–DNA at pH of 7.4 by fluorescence spectral titration

Compounds	Binding constants for DNA/BSA with test compounds									
	SV methods for determining DNA binding characteristics (SV methods for determining BSA binding characteristics)*						LWB method *K*_LB_ × 10^4^ M^−1^	Scatchard analysis		*K*_app_ × 10^7^ M^−1^
	Method-I		Method-II							
	*K*_q_ × 10^12^ M^−1^ s^−1^	*K*_SV_ × 10^4^ M^−1^	*K*_ass_ × 10^4^ M^−1^	*n*	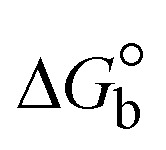 (kJ M^−1^)	*P*		*K*_SA_ × 10^4^ M^−1^	*n*	
(**HL**)	1.1636 (2.639)	1.1636 (2.639)	0.9606 (1.062)	0.973 (0.926)	−2.720 (−23.0)	0.0899 (0.464)	0.6985	1.9093	1.158	0.5829
(1)	1.6755 (4.651)	1.6755 (4.651)	1.1954 (2.005)	1.037 (0.954)	−3.261 (−24.5)	0.0902 (0.287)	0.7149	3.0094	0.983	0.5926
(2)	2.8863 (7.074)	2.8863 (7.074)	3.2903 (5.455)	1.104 (1.018)	−5.770 (−27.0)	0.1516 (0.385)	1.3497	3.3185	1.056	0.8349
(3)	1.6879 (4.8055)	1.6879 (4.806)	1.0350 (5.292)	1.024 (1.051)	−2.905 (−26.9)	0.0950 (0.348)	0.7786	3.2363	0.971	0.7340

*K*_SV_ denotes the Stern–Volmer binding constant; *K*_ass_ represents the association binding constant; *K*_app_ represents the apparent binding constant, 
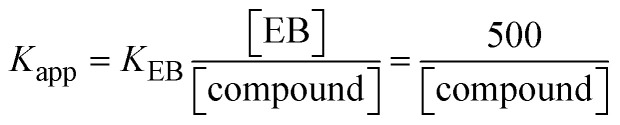
; *K*_EB_ = 10^7^ M^−1^ at a concentration of 50 μM EB; Gibb's free energy change 
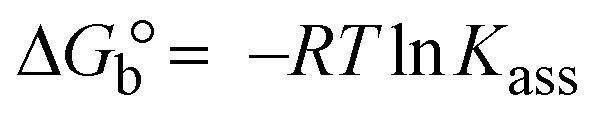
; *K*_q_ represents the bimolecular quenching rate constant/Stern–Volmer dynamic quenching rate constant 
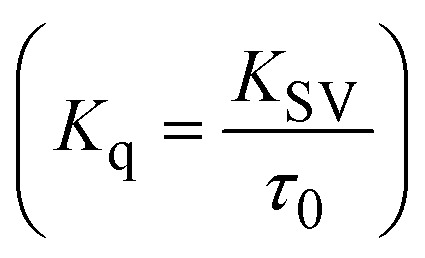
, average lifetime of biomolecular quenching in the absence of a quencher (*τ*_0_) = 10^−8^ S; Gibb's free energy change 
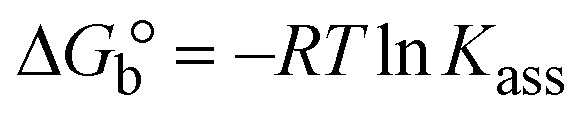
 (where *R* = 8.3144 kJ mol^−1^, *T* = 298 K); *K*_LB_ represents the Lineweaver–Burk (LWB) binding constant; *K*_SA_ represents the Scatchard association binding constant; *K*_app_ denotes the apparent binding constant; *n* is the number of binding sites; *P* is the ratio of fluorescence quantum efficiency of DNA bound and free complexes 
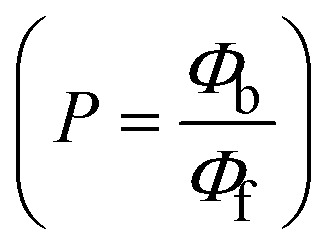
, which is obtained as the intercept from the plot *F*/*F*_0_*vs.* 1/[DNA]; error limit ± 2.5% (*P* < 0.025).

12 *F*_corr_ = *F*_obs_ × e^[(*A*_ex_×*d*__ex__) + (*A*__em__×*d*__em__)]/2^ = *F*_obs_ × e^(*A*_ex_+*A*__em__)/2^;

where *F*_corr_ and *F*_obs_ represents the IFE-corrected fluorescence and observed (uncorrected) emission intensities, respectively; *d*_ex_ and *d*_em_ denote the cuvette path lengths in the excitation and emission directions, respectively; *A*_ex_ and *A*_em_ represent the change in absorbance at the excitation and fluorescence wavelengths, respectively.13
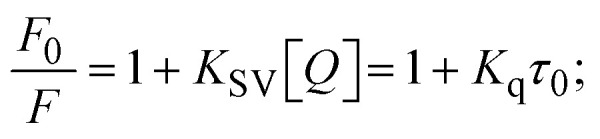
where [*Q*] is represented as the sample concentration, the emission intensities *F*_0_ and *F* of DNA/BSA in the absence and presence of the quencher (sample), respectively.14
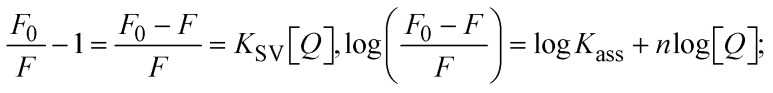

15 *K*_EB_[EB] = *K*_app_[compound];

16
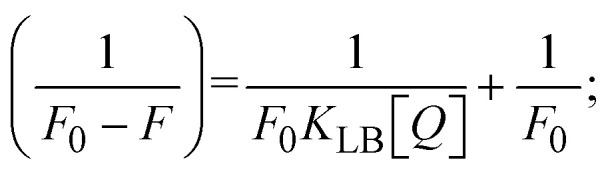
17
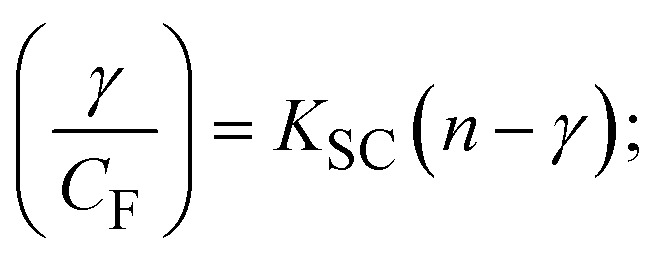
where *γ* = [(*F*_0_ − *F*)/*F*_0_], and *C*_F_ denotes the concentration of the sample alone.

The findings for *K*_ass_ and *n* were evaluated from the linear regression plot of log(*F*_0_ − *F*)/*F vs.* log[*Q*] by SV method II with the help of eqn (14) (Fig. S22, S28† and Table 4). *ε* findings of all substances were observed from the linear regression plot of emission intensity *vs.* [compound] with the help of the Beer–Lambert law equation (*A* = *εcl*) (Fig. S25†) and eqn (15) is applied to evaluate *K*_app_ values using *K*_EB_ = 10^7^ M^−1^ at 50 μM concentration and to measure the sample concentrations for all cases using the Beer–Lambert law equation. The complex concentration's IC_50_ findings were estimated at a 50 percentage diminution in the emission intensity of ethidium bromide. The Lineweaver–Burk eqn (16) and Scatchard analysis eqn (17) are utilized to expand the observations and validate the binding affinities^78,79^ and the observations are also compared with the Stern–Volmer method. Eqn (16) was used to determine the value of *K*_LB_ from the linear regression plot of 1/(*F*_0_ − *F*) *vs.* 1/[*Q*] (Fig. S22†). *K*_SA_ and *n* values were also measured from the linear regression plot of (*γ*/*C*_F_) *vs. γ* by eqn (17) (Fig. S23†) and the overall measured DNA/BSA binding constants (*K*_SV_, *K*_ass_, *K*_app_, *K*_LB_ and *K*_SC_) for all samples were in the following order: (2) > (3) > (1) > (**HL**). The *n* values acquired from the Stern–Volmer eqn (14) and the Scatchard eqn (17) were in the range of 0.9733–1.1040 and 0.9711–1.1580, respectively, for all compounds (Table 4). In addition, the neighbor-exclusion principle is one of the most imperative and well-known rules governing the intercalative binding of small planar molecules to DNA. It implies that such binding is only possible at base-pair sites where there is an opposite base pairing, which indicates extremely negative cooperativity in the binding process. This rule states that the two neighboring sites of an occupied intercalation site in DNA must stay unoccupied, or, in less absolute terms, intercalation is negative-cooperative (anti-cooperative) at adjacent sites. In other words, the next-neighbor (second) intercalation site along the length of the DNA double helix remains unoccupied. Moreover, the neighbor-exclusion principle is a vibrational entropy effect, which is associated with polyelectrolyte (counterion release) effects and further demonstrated with negative cooperativity effects in ethidium and actinomycin binding to DNA.^80^ However, the highly stable complexes observed with CT-DNA, poly[*d*(*A*–*T*)], *d*(CCGGAATTCCGG), and *d*(CGCGAATTCGCG) all have dissociation constants in the range of 1 to 3 × 10^−9^ M^−1^. On CT-DNA, these complexes develop at a rate of around 1 binding site every 100 base pairs. The neighbor-exclusion principle for intercalation binding modes states clearly that the number of binding sites (*n*) depends on the character of the intercalators and their neighboring environments. When the first small molecules interact with the binding site of DNA, intercalation occurs with or without allowing the second small molecule. If the first binding intercalator increases the affinity of the second site, there is positive cooperativity (*n* > 1) due to the support of other non-covalent interactions. This case violates the neighbor-exclusion rule. If the first binding intercalator decreases the affinity of the second site, there is negative cooperativity (*n* < 1). On the other hand, if there is no impact on the second site, there is non-cooperativity (*n* ≈ 1).^81^ Later, two cases obey the neighbor-exclusion rule. However, our present complexes show better intercalation binding affinity than a free ligand owing to the value of *n* being nearly equal to one (Table 4). Consequently, it is proposed that the complexes contain both a 2,2′-bipyridine ring planar system and an aromatic ring system linked with morpholine. They can effectively interact with DNA *via* intercalation. Additionally, the values of the fluorescence quantum efficiency (*P*) ratio for the DNA and BSA–complex adducts were 0.0899–0.1516 and 0.2870–0.4640, respectively, which were measured from the linear regression plot of *F*/*F*_0_*vs.* 1/[DNA] and 1/[BSA], respectively (Fig. S24, S29† and Table 4). These results and those from the viscosity, electrochemical titration, and UV-vis spectral properties were in good agreement.

#### 3.2.6. Förster's theory-based FRET computation

FRET is a non-destructive spectroscopic method consisting of a process between several electronic excited states of molecules that is dependent on distance (*r*). FRET can also be employed to distinguish the relative angular orientation and closeness of fluorophores.^82^ The process happens when there is a large overlap between the absorption spectrum acceptor (compound/chromophore) and the donor's emission spectrum (BSA/fluorophore) (Fig. S30†). Also, the average distance (*r*) between the donor and acceptor can be measured in accordance with this theory. Fluorescence is quenched due to energy being transmitted from the excited state of BSA to the substances (**HL**)/(1–3). As a result of the FRET analysis, the observed *r* findings were in the range of 2.4127–2.7129 nm (Table 5 and Fig. S30†). This also shows that there is a high probability that energy will be transported from BSA to the compounds. The following conditions have a major impact on the effectiveness of FRET: (i) the distance (*r*) should be in the prescribed range of 2–8 nm for energy transfer; (ii) there is a large overlap between the emission spectrum of biomolecules (donors) and the electronic absorption spectrum of acceptors (substances); and (iii) the BSA and compound transition dipoles are oriented correctly. BSA transmits excitation energy to a compound during FRET without emitting a photon from the previous molecule system. Energy transfer (*E*) results were acquired from eqn (18) (Table 5). *K*^2^ is associated with the geometry of the BSA and complex of the dipoles, the value for random orientation (*K*_2_ = 2/3) as in a fluid solution. Basically, the *K*^2^ values were found in the range from 0 to 4, and energy can be transferred from BSA to the compound when electrons are transferred between the two molecules. For parallel transition dipoles that are aligned, *K*^2^ is equal to 4, which denotes maximal energy transfer; and when the orientation of the dipoles is perpendicular to one another, *K*^2^ is equal to 0, which denotes very weak energy transfer. When the relative orientation of the dipoles is random, *K*^2^ is equal to 2/3. Eqn (20) is helpful for measuring the *J* values for overlap of the emission spectrum of BSA with the electronic absorption spectrum of the compound. The molar absorption coefficient (*ε*_A_) and fluorescence emission intensity were both measured on the unit area scale of wavenumbers. It is imperative that *J*, after being normalized, is independent of the real size of *ε*_A_. The following variables for the complex–BSA interaction are determined using eqn (18)–(23), *n* = 1.36, *Φ* = 0.15, *E* = 0.3462–0.5692, *J* = 0.8215–1.0886 × 10^−14^ cm^3^ L mol^−1^, *R*_0_ = 2.4400–2.5573 nm, *r* = 2.4127–2.7129 nm, *k*_ET_ = 5.2941–13.2142 J s^−1^ and *B* = 5339.79–6007.23 mol^−1^ cm^−1^ (Table 5). The observed values of *R*_0_ and *r* between BSA Trp213 and the interacting compound were substantially smaller than 8 nm and their relationships are found in the following sequence: 0.5*R*_0_ (1.2200–1.2786) < *r* (2.4127–2.7128) < 1.5*R*_0_ (3.6601–3.8358). The test substance and BSA had a high probability of exchanging non-radiative dipole–dipole energy, which was consistent with a static quenching process. This result proved that the binding adhered to the conditions of Förster's energy transfer theory. *Φ* is defined as the dimensionally invariant ratio of photons emitted to photons completely absorbed by a fluorophore, and it serves as a tool for estimating the effectiveness of fluorescence emission in correlation with all other channels of relaxation. Also, *τ* is denoted as the lifetime of fluorescence emission of the biomolecule and is described as the inverse of the entire degradation rate *τ* = 1/(*k*_r_ + *k*_nr_). The radiative lifetime of the fluorophore is represented as *τ*_0_ = 1/*k*_r_. The values of *τ* and *Φ* are associated with eqn (21) (Table 5). Quenching occurs when the ground or excited states of BSA come into contact with a compound in the solution. The intensity of fluorescence emission is also decreasing. Quenching is divided into the two main categories of dynamic and static quenching. While BSA is in an excited state, it binds with the substance during a dynamic or collisional quenching mechanism, and is then deactivated to the ground state without the emission of radiation. Therefore, the concentration of the quenching compounds affects dynamic quenching. The *τ* and *Φ* values for BSA diminish with an increase in the compound concentration. Conversely, static quenching reduces fluorescence emission without changing the excited state *τ* or *Φ*, and quenching can be divided into two main categories based on the excited-state lifetime of the fluorophore. Additionally, the term *k*_q_[*Q*] is included in the denominator in eqn (21) and the *Φ* value for the BSA–compound adduct system is measured with eqn (21). FRET requires an interaction between the emission and absorption transition dipole moments of BSA and the test compound, respectively, due to the non-radiative transfer of excitation energy from a fluorophore to a chromophore.^83^*k*_ET_ is dependent on not only the overlapped spectrum of emission of BSA and the absorbance of the compound but also on the *Φ* values of BSA, *K*^2^ and *r*, *etc.* The *k*_ET_ values for all substances were estimated with eqn (22) (Table 5). *K* is denoted as a relative factor of the specific orbital interactions between BSA and substances based on orbital overlap. The Förster radius is the distance at which resonance energy transfer is 50% proficient (*R*_0_).^84^ Also, the brightness of BSA depends on the ability of a test compound to absorb light and the *Φ* value, which is calculated with expression eqn (23). Chemical compounds with high absorbance have higher values for *ε* and *Φ*, which also promote effective emission.

**Table 5:** FRET parameters for donor (BSA)–acceptor (compound) systems

Compounds	*J* × 10^−14^ (LM^−1^ cm^3^)	*R*_0_ (nm)	*E*	*r* (nm)	*k*_ET_ (J s^−1^)	*B* (M^−1^ cm^−1^)
(**HL**)	0.8215	2.4400	0.3462	2.7129	5.2941	5339.79
(1)	1.0886	2.5573	0.4462	2.6511	8.0556	6007.23
(2)	1.0201	2.5297	0.5000	2.5297	10.000	5835.99
(3)	1.0145	2.5274	0.5692	2.4127	13.2142	5822.04



18

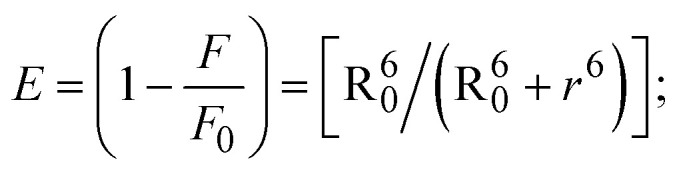

when the transmittance efficiency is 50%, the observed critical distance is *R*_0_, which denotes the Förster radius characterizing the donor/acceptor pair and is evaluated from eqn (19).

19 R^6^_0_ = 8.79 × 10^−25^*K*^2^*n*^−4^*ΦJ*;

20
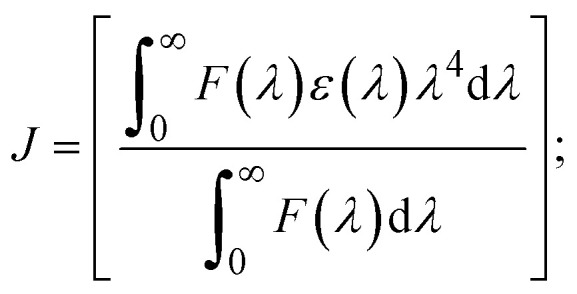
where *J* denotes the normalized spectral overlap integral between the emission spectrum of the donor (BSA) and the absorption spectrum of the acceptor (complex); *R*_0_ is the critical distance at which the efficiency of resonance energy transfer (50%) 
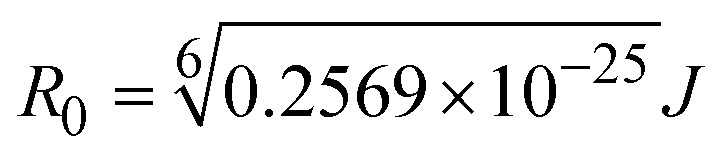
; average refracted index of medium (*n*) = 1.36; fluorescence quantum yield of the donor (*Φ*) = 0.15; orientation factor related to the geometry of the donor and acceptor of the dipoles (*K*^2^) = 2/3 for the complex–BSA interaction; *E* represents the efficiency of energy transfer, 
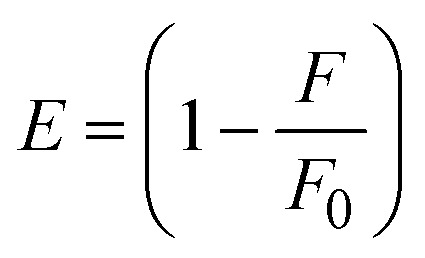
; *F* and *F*_0_ are the fluorescence intensity of BSA in the presence and absence of the complex; *r* is the donor–acceptor separation relative to their van der Waals radii *L* (nm), 
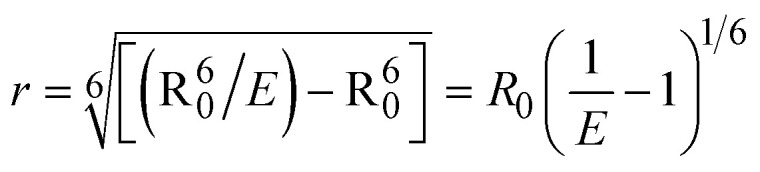
; *F*(*λ*) represents the corrected or normalized emission intensity of BSA in the wavelength range of *λ* − (*λ* + Δ*λ*); *ε*(*λ*) denotes the molar absorption coefficient of the compound at *λ*.21
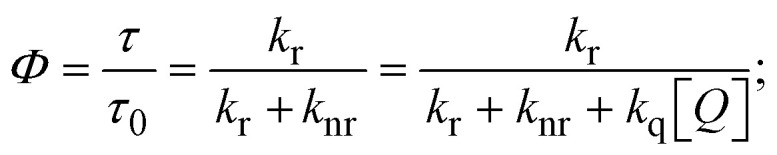
where the radiative, non-radiative decay and quenching rate constants are denoted as *k*_r_, *k*_nr_ and *k*_q_, respectively; *τ*_0_ → radiative lifetime of the fluorophore (biomolecules) (*τ*_0_ = 10^−8^ s); and the concentration of complex (quenching species) is described as [*Q*].22
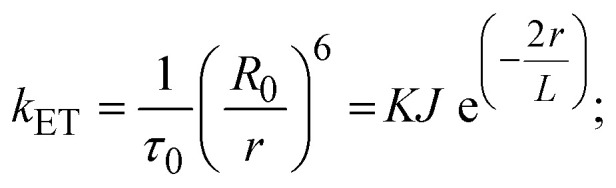

23 *B* = *Φε*;

*k*_ET_ denotes the rate of exchange resonance energy transfer; *B* → average brightness of the complex–BSA system, *B* = [(*Φ*_1_*ε*_1_ + *Φ*_2_*ε*_2_)/2]; *ε* is molar absorption or extinction coefficient of the acceptor at *λ*, *ε* = 43 824 LM^−1^ cm^−1^ for the donor (BSA) and *ε* values for the acceptors = 27 373.20 (**HL**), 36 272.40 (1), 33 988.20 (2), and 33 803.20 (3). *B* value of free BSA = 6573.60 M^−1^ cm^−1^.

#### 3.2.7. Analysis of DNA binding characteristics using the CV method

The CV approach is one of the most important methods for evaluating the binding mechanism of a DNA–complex adduct. The CV properties of all test samples in the presence and absence of DNA were executed at a scan rate (*v*) of 0.1 V s^−1^ with a potential range of +2 to −2 in a Tris–HCl (5 mM)/NaCl (50 mM) (pH = 7.2) solution. The M^1+^/M^2+^ redox couple is caused by complexes that reveal a single anodic and cathodic peak. The complex's reaction with the one-step, one-electron process demonstrated by the glassy carbon electrode surface, is a quasi-reversible redox process since the (*I*_pa_/*I*_pc_) ratio values for the redox couple are about one, which is also supported by the change in peak potential separation (Ep > 0.0591 V)^85–87^ (Fig. S31† and Table 6).

**Table 6:** Redox potential patterns for the interaction of DNA with ligand (**HL**) and its complexes (1–3)

Compounds	Δ*E*_P_ (V)	*E*° (or *E*_1/2_) (V)	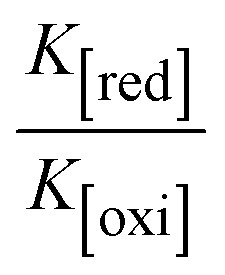	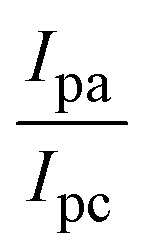	*D*_o_ × 10^−5^ cm^2^ s^−1^	*K*_b_ × 10^4^ M^−1^ (methods)			*S* (bp)
	Free (bound)	Free (bound)	Found (I) (Calcd)	Free (bound)	Free (bound)	I red (Oxi)	II	III	
(**HL**)	0.7420 (0.8890)	0.3490 (0.3680)	0.7214 (2.0964)	1.4295 (1.3424)	2.8570 (2.5809)	0.3809 (0.528)	0.2443	0.4837	0.452
(1)	0.3801 (0.1477)	0.7126 (0.7310)	0.7874 (2.01)	0.6194 (0.4800)	3.1445 (3.1295)	0.7890 (1.002)	1.4873	1.0713	0.287
(2)	0.2790 (0.1553)	0.8015 (0.8177)	0.7973 (1.86)	0.6307 (0.4073)	4.1374 (4.0684)	1.1330 (1.421)	4.1119	1.5999	0.125
(3)	0.2686 (0.1375)	0.7528 (0.7688)	0.8003 (1.86)	1.8405 (0.2189)	3.7063 (3.1983)	0.9156 (1.144)	0.6072	1.6031	0.382

Δ*E*_P_ is peak-to-peak separation = (*E*_Pa_ − *E*_Pc_); *E*° (or *E*_1/2_) denotes the formal electrode potential = 1/2(*E*_Pa_ + *E*_Pc_); 
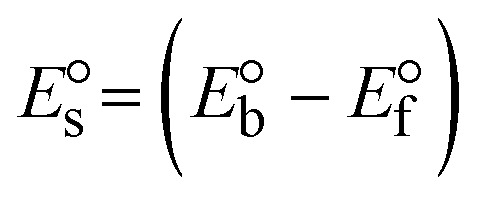

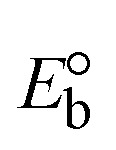
 and 
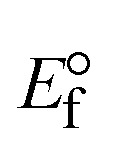
 represent the formal electrode potential of the M^1+^/M^2+^ couple in the bound and free forms, respectively. 
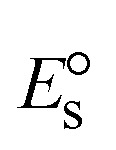
 = +19 mV (**HL**), +18 mV (1), +16 mV (2), +16 mV (3). *I*_pa_ is anodic peak current, *I*_pc_ is cathodic peak current. K^1+^ is the binding constant of the reduction process, K^2+^ is the binding constant of the oxidation process. *S* represents the binding site size of base pairs (bp) with a molecule of complex, scan rate is 100 mV s^−1^, binding constant (*K*_b_) values observed from the linear plots of log(1/[DNA]) *versus* log(*I*/*I*_0_ − *I*) for oxidation and reduction, (*I*_0_ − *I*_DNA_)/*I*_DNA_ = *C*_p_/*C*_f_*versus* [DNA] and *I*^2^_p_ *versus*(*I*^2^_po_ − *I*^2^_p_)/[DNA] by methods I, II and III, respectively. Diffusion coefficient 
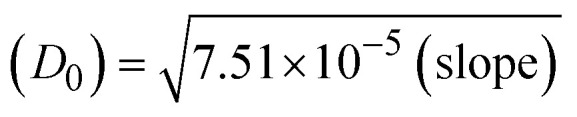
.24
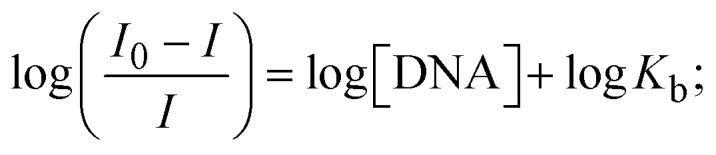
where *I*_0_ and *I* represent the peak currents of the compound in the absence and presence of DNA.25
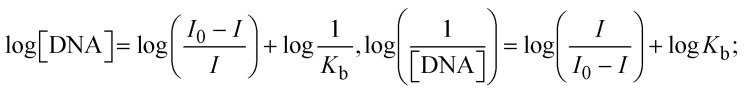
26
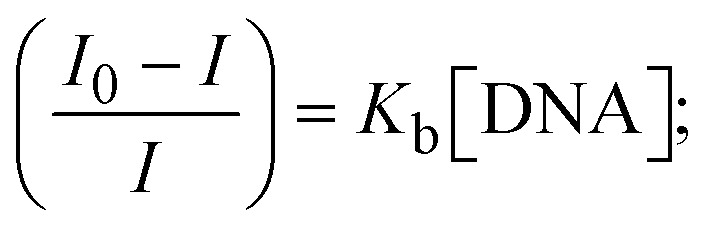
27
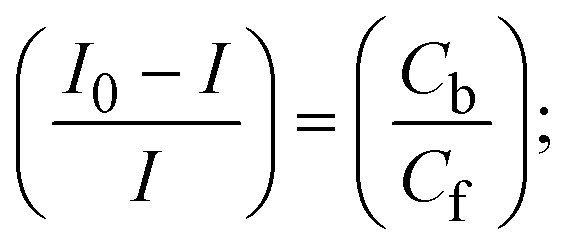
*C*_f_ and *C*_b_ denote as the free substance concentration and DNA-interacted compound, respectively.Eqn (28) was obtained by comparing eqn (26) and (27).28
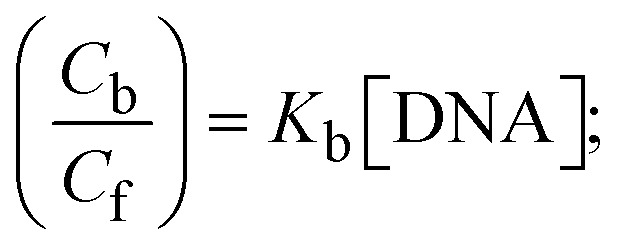
29
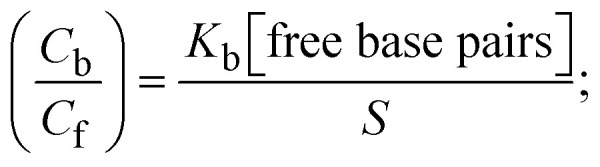
30
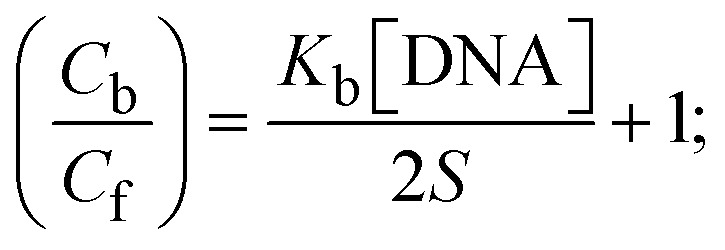
*S* denotes the binding site size (bp) and *K*_b_ are estimated from eqn (30) with the help of *S* = (intercept/4)^1/2^ and *K*_b_ = 2*S* (slope/intercept), respectively.31
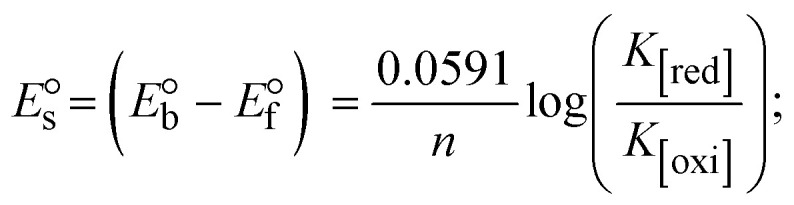
32
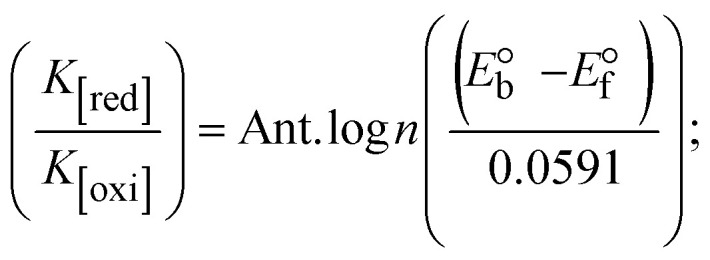
33
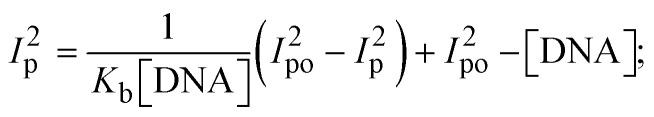
where, *I*_po_ and *I*_p_ denote the peak currents of complexes (1–3) in the absence and presence of DNA.34
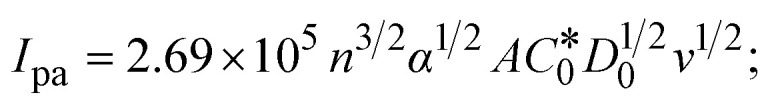

35 *I*_pa_ = 13314.7*D*^1/2^_0_*v*^1/2^;

where *I*_pa_ denotes the anodic peak current in amperes, *n* represents the number of electrons participating in the redox (M^1+^/M^2+^) process (*n* = 1), charge transfer coefficient or activation coefficient (*α*) ≈ 0.5 for quasi-reversible systems, which is also calculated from the Bard–Faulkner relation,

35a *α* = [47.7/(*E*_Pa_ – *E*_*P*/2_)];

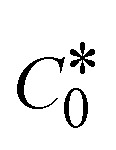
 → bulk concentration of the compound; *A* denotes the cross-sectional area of the working electrode (glassy carbon) in cm^2^ (*A* ≈ 0.07 cm^2^); *D*_0_ denotes the diffusion coefficient (cm^2^ s^−1^) of the M^1+^/M^2+^ couple in the free and bound forms, respectively; and *v* denotes the potential scan rate at 0.1 V s^−1^.

While the substances often bind to DNA through intercalation, the peak potential shifts in a positive direction. When the compounds bind to DNA through minor or major grooves or electrostatic attractions, the peak potential shifts occur in a negative direction. It was observed that the consistent movement of peak potential shifts in the positive direction during increments of DNA with test substances. The binding mode in compound–DNA adducts has been described as primarily intercalation (Fig. S31†), and it is also attributed to the presence of 2,2′-bipyridine and morpholine fused aromatic planar systems in mixed ligand complexes, which can create inclusion through intercalation due to hydrophobic and π–π stacking interactions in the DNA base pairs. It is also supported by the evaluated outcomes from UV-vis spectral, emission titration, viscometric, and biothermodynamic properties. Furthermore, the observed values of *K*_b_, *S*, and the ratio of binding constants (K^1+^/K^2+^) for M^1+^/M^2+^ couple systems further confirmed the binding affinity *via* intercalation. Also, the subsequent eqn (24)–(35) are applied to determine the above parameters.^88,89^Eqn (24) is acquired from modification of the Stern–Volmer eqn (14) (Table 6). The *K*_b_ values for all samples were estimated from the linear regression plot of log(1/[DNA]) *versus* log(*I*/*I*_0_ − *I*) by method I (Fig. S32† and Table 6). Eqn (26) was obtained from revision of eqn (25). Also, binding site size (*S*, bp) and *K*_b_ are estimated from eqn (30) from the linear regression plot of (*C*_p_/C_f_) *versus* [DNA] by method-II^90–92^ (Fig. S33† and Table 6). In addition, base-pair sites with a molecule of the compound are referred to as “binding site size” (*S*), and the evaluated *S* values were found in the range from 0.1718 to 0.4599 bp. In general, if the *S* value is less than 1, it denotes stronger binding through intercalation, and if the *S* value is greater than 1, this suggests the possibility of the modes of groove binding or electrostatic interactions.^93–97^ The *S* value also suggests that there should be one binding site for every two base pairs, denoting that complex (1a) has revealed superior binding efficiency to the others owing to its robust binding affinity with DNA through intercalation, and its measured results for *S* were also in the range of 0.1250–0.4520 bp (Table 6). It can therefore be stated that a compound or medication exhibits high binding affinity when it occupies a single binding site. Meanwhile, the drug–DNA adduct exhibits low binding affinity when many site sizes are increased at the same time.^98^ In the Nernst eqn (31) and (32) for the galvanic cell, 
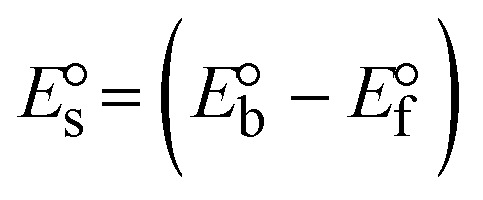
, the formal electrode potentials of the M^1+^/M^2+^ couple in their bound and free forms are *E*_1/2_ or 
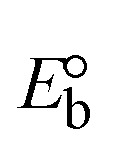
 and 
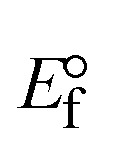
, respectively. As a result of the variable binding state [M^1+^/M^2+^] and the delayed mass transfer of test compounds that interacted with DNA fragments, the addition of DNA to the compound solution enabled a change in the redox potential to a higher positive direction and a drop in both anodic and cathodic peak currents. In particular, the equilibrium of M^1+^/M^2+^ is influenced by electrostatic or hydrophobic interactions. However, the 
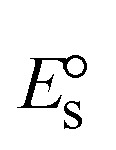
 values of M^1+^/M^2+^ for all substances were observed to be positive values (Table 6). This suggests that the compounds' strong hydrophobicity makes their interactions with DNA through intercalation more favorable. On the other hand, if the value of 
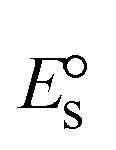
 is negative, this indicates that the substance interacts more favourably with DNA through electrostatic interactions, and K^1+^ and K^2+^ are represented as binding constants for the binding states of the +1 and +2 chemical substances to DNA respectively. The number of electron transfers is given by *n*, which is equal to one. With the aid of eqn (25), *K*_[red]_/*K*_[oxi]_ values for reduction and oxidation processes were determined from the linear regression plot of log(1/[DNA]) *versus* log(*I*/*I*_0_ − *I*) (method I), which was also estimated using the Nernst eqn (32) (Table 6). Generally, the DNA–compound adduct is assigned the groove binding or electrostatic binding interaction when the value of the ratio [K^1+^/K^2+^] is equal to one. When the value of the ratio is less than or greater than one, it demonstrates that the mode of intercalation binding could take place in the DNA–compound system owing to hydrophobic forces of attraction.^99,100^ The following mechanism led to the latter finding in the compound–DNA systems (Table 6).

The *K*_b_ value was evaluated from the reciprocal of the slope in the linear regression plot of *I*^2^_p_*versus* (*I*^2^_po_ − *I*^2^_p_)/[DNA] with the help of method III (Fig. S34† and Table 6). In these cases, complex (2) shows greater binding effectiveness than the others owing to its robust binding affinity with DNA through intercalation. As a result, it is proposed that complexes consist of an aromatic planar system linked with a morpholine moiety as well as 2,2′-bipyridine planar systems that may firmly interact with DNA through intercalation, which is also confirmed by the value of the diffusion coefficient (*D*_0_) of the compound alone and the DNA-bound compound with the aid of the subsequent quasi-reversible Randles–Sevcik eqn (34),^101^ which is a typical approximation for numerous quasi-reversible systems for the redox (M^1+^/M^2+^) process. The values of *I*_pa_ and *α* were also measured from eqn (35) and (35a) (Table 6).^102^ When all compounds were treated with DNA, the anodic and cathodic peak currents of M(i)/M(ii) reduced due to a decrease in the diffusion coefficient (*D*_0_). The findings obviously suggested that the evaluated values of *D*_0_ of DNA-bound compounds were less than those of the free test compounds. The values of *D*_0_ of all samples in the absence and presence of DNA at scan rates of 0.01–0.3 V s^−1^ were measured from the linear regression plots of _f_Ipa *vs. v*^1/2^ and _b_Ipa *vs. v*^1/2^ using eqn (35)^103–105^ (Fig. S35† and Table 6).

### 3.3. Evaluation of BSA binding by UV-vis spectral titration

**Fig. 4:**
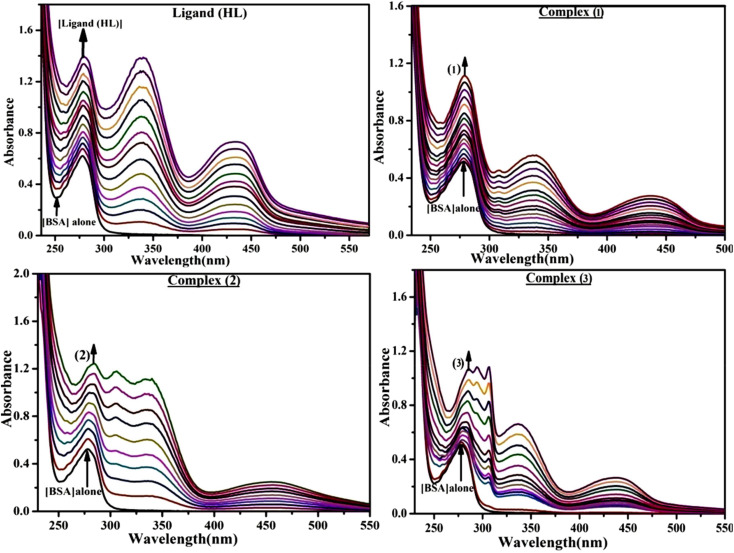
UV-visible titration spectra of bovine serum albumin at room temperature in Tris–HCl buffer at a pH of 7.2 in the absence and presence of increasing amounts of test substances. Arrow shows the changes in absorbance upon increasing the substance concentration.

Electronic absorption titration analysis can be used to determine the types of quenching mechanisms, which are classified into two main categories: static and dynamic. In this case, when the concentration of each test compound increases, the BSA absorption intensity also increases with a noticeable blue shift, suggesting that the interactions between BSA and the complexes are static rather than dynamic. BSA is a crucial plasma protein that transports different endogenous and exogenous proteins, hormones, ions, and medications, as well as helping to maintain blood pH and osmotic blood pressure. BSA contains 583 amino acids and has a spherically organized protein structure. Two tryptophan residues in BSA, tryptophan-134 and tryptophan-213, are primarily responsible for the protein's intrinsic fluorescence. Fig. 4 displays the electronic absorption spectra of BSA at various concentrations of test compounds in Tris–HCl buffer solution. An increase in absorption maxima along with the blue (hypsochromic) shift were observed when the concentration of test compounds was raised from 0 to 25 μM in the absorption titration of BSA. Owing to the presence of aromatic amino acids, such as phenylalanine, tyrosine, and tryptophan, on the surface of the protein chain, the absorption maxima at 278 nm are attributed to the π–π* transition. The chemical substance interacts with Tyr and Trp amino acids without changing the preferred conformation of BSA, which is indicated by a blue shift in the absorption spectra. This change in the BSA absorption spectra reveals the best interactions between BSA and the test chemicals. The titration was performed for BSA in the presence and absence of test substances in a Tris–HCl (pH = 7.2) solution (Fig. 4 and Table 7). Quenching typically occurs in either a static or a dynamic phase. The static quenching mechanism involves only the synthesis of BSA–compound in the ground state, but a dynamic quenching mechanism involves the temporary presence of the excited state, which brings BSA and the compound into close proximity. In addition, the dynamic quenching mechanism has no effect on the absorption spectrum; it affects only the excited state.^106^ The absorption intensity for BSA was found to be between 278 and 280 nm. When the test sample concentration increases, the absorbance values also increases accompanied by the blue shift (hypsochromic) (2–3 nm). It is suggested that BSA and the test compounds in the ground state interact statically. In this case, the evaluated hyperchromism was found in the range of 47.79 to 57.41%. The findings also clearly show that conformational changes may happen owing to non-covalent interactions like H-bonds and electrostatic interactions between substances and BSA. The Benesi–Hildebrand eqn (36) is used to evaluate the *K*_b_ values^107^ (Table 7). The *K*_app_ findings for all substances were estimated from the linear regression plot of [(*A*_*∞*_ − *A*_0_)/(*A*_*x*_ − *A*_0_)] *vs.* {1/[compound]} M^−1^ (Fig. S36†). The evaluated *K*_b_ findings for all test substances were in the following order: (2) > (3) > (1) > (**HL**) with 
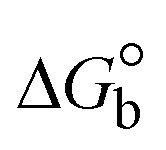
 values from −22.1246 to −25.6174 kJ mol^−1^. Complex (1a) is also clearly shown to have the greatest spontaneous binding efficacy with BSA.

**Table 7:** UV-vis titration parameters for all substances bound to BSA

Compounds	*λ*_max_ (nm)		Δ*λ* (nm)	Chromism (% *H*)	Binding constant *K*_app_ × 10^4^ M^−1^ by BH method	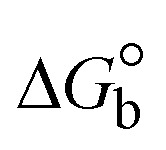 (kJ mol^−1^)
	Free	Bound				
(**HL**)	278	276	02	47.79	0.7556	−22.1246
(1)	278	275	03	57.41	1.2429	−23.3580
(2)	280	278	02	50.43	3.0937	−25.6174
(3)	280	278	02	54.61	1.8374	−24.3265

Hyperchromism 
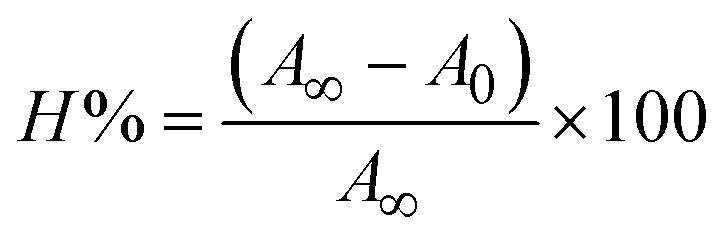
; *A*_0_ denotes the absorbance of BSA alone at 278 nm; *A*_*∞*_ represents the absorbance of the fully bound form of BSA with a complex or ligand; and *A*_*x*_ is the absorbance of BSA with the addition of different concentrations of complex or ligand. Gibb's free energy change 
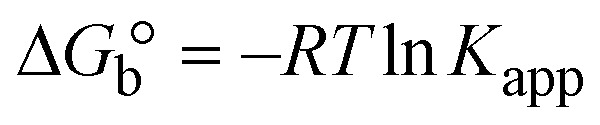
 (where *R* = 8.3144 kJ mol^−1^, *T* = 298 K); *K*_app_ denotes the apparent binding constant evaluated from the UV-vis absorption spectral titration.36
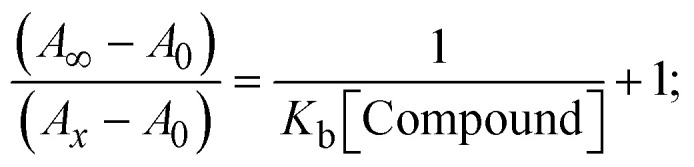
where Δ*A*_max_ = (*A*_*∞*_ − *A*_0_), Δ*A* = (*A*_*x*_ − *A*_0_); *A*_0_, *A*_*x*_ and *A*_*∞*_ denote the absorbance of free BSA, the absorbance of BSA with increments in the concentrations of the compound, and the absorbance value of the fully bound form of bovine serum albumin with the substance, respectively. Error limit ± 2.5%.

### 3.4. Computational molecular spectroscopy

#### 3.4.1. Molecular modelling: DFT calculations

**Fig. 5:**
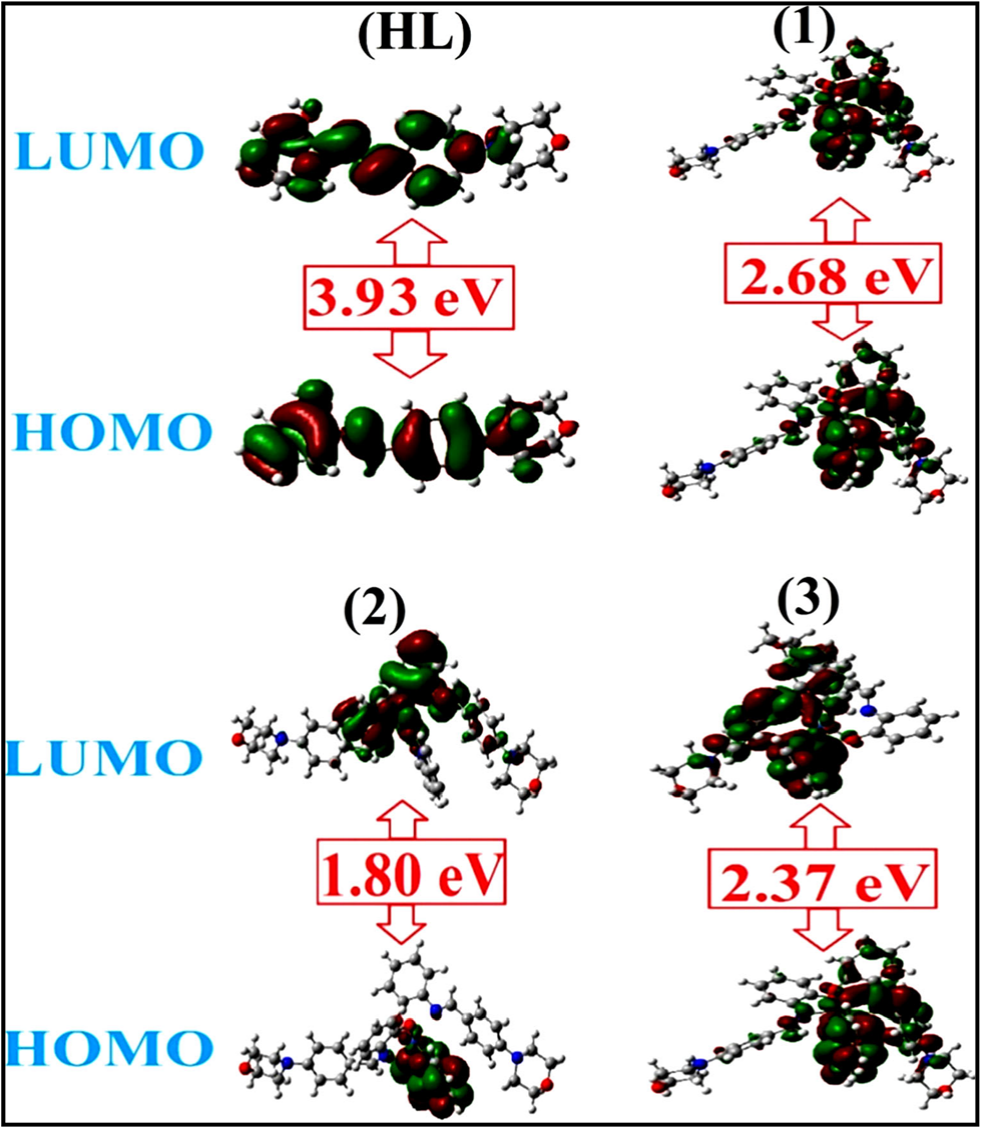
FMOs of the free ligand (**HL**) and its complexes (1–3).

Density functional theory (DFT) is the most popular quantum chemistry methodology for the simulation of energy surfaces in molecules and other periodic systems. Herein, DFT computations were undertaken to characterize the electronic structure and elucidate the reactivity of the free ligand and the metal complexes (1–3). The B3LYP functional, in conjunction with the 6-31G(d) and Lanl2dz basis sets, was used to fully optimize all of the compounds. The latter basis set was applied to the transition metals and allowed us to effectively mitigate the computing cost associated with the usage of all-electron basis sets on heavy atoms. Further normal mode calculations established the supposed static points to be actual minima on the potential energy surface.^108^ Optimized geometries for ligand (**HL**) and complexes (1–3) are shown in Fig. S37,† which also indicate the calculated dipole moment (DM) for each compound. Complexes (1–3) are found in either the singlet or doublet ground states, depending on the electronic structure of the central metal. Note that the electronic configurations of metal(ii) complexes (1–3) are 3d^5^ (1), 3d^7^ (2), and 3d^8^ (3), respectively, which should exhibit paramagnetic characters due to a weak field and a high-spin ligand. In particular, complex (3) has two unpaired electrons in the e_g_ high energy level orbital and six electrons in the t_2g_ low energy orbital, in accordance with crystal field theory. In line with the experimental results, all-metal complexes present an octahedral geometry, where the central metal is bound to six coordination sites, including the two phenolic O and iminic N atoms of the ligands, along with the two N atoms of the bipyridine molecule. The dipole moment is a measure of the polarity or charge separation in molecular systems or just along a chemical bond path, and it characterizes intermolecular interactions between non-bonded subunits. The observed dipole moment values of complexes (1–3) ranged from 7.60 to 9.94 Debye, which is more polar than the free ligand (**HL**) (0.86 Debye) (Table 8). Several research reports have shown that the interaction between the highest occupied molecular orbital (HOMO) and lowest unoccupied molecular orbital (LUMO) is primarily responsible for determining chemical reactivity. It is generally acknowledged that a compound's kinetic stability can be attributed to the energy gap (Δ*E*) between its FMOs. A rule of thumb is that a larger energy gap indicates that the system is more kinetically stable. In the present case, HOMO–LUMO energy gaps of 3.925 (**HL**), 2.682 (1), 1.804 (2), and 2.368 eV (3) were predicted for all compounds (Fig. 5 and Table 8). These findings also proposed the following order of kinetic stability: (**HL**) > Mn (1) > Ni (3) > Co (2), which further indicates that all synthesized complexes (1–3) are more reactive than the free ligand, which also points out that complexes (1–3) may have better binding profiles with biomolecules.

**Table 8:** Quantum chemical parameters (eV) or global reactivity descriptors as well as FMO energy gap and dipole moment values of free ligand (**HL**) and metal complexes (1–3)

Compounds	*E*_LUMO_ (eV)	*E*_HOMO_ (eV)	Δ*E* (eV)	*χ*	*η*	*σ*	*μ*_i_	*ω*	Δ*N*_max_	*μ* (Debye)
(**HL**)	−1.192	−5.177	3.925	3.184	1.992	0.502	−3.184	2.544	1.598	0.86
(1)	−1.717	−4.399	2.682	3.058	1.341	0.746	−3.058	3.487	2.280	7.60
(2)	−2.271	−4.075	1.804	3.173	0.902	1.109	−3.173	5.581	3.518	9.94
(3)	−1.749	−4.117	2.368	2.933	1.184	0.845	−2.933	3.633	2.477	9.48

Electron volt (eV). Δ*E* (eV) → energy gap between HOMO and LUMO. HOMO → highest occupied molecular orbital which is directly related to ionization potential (*I*_P_ = −*E*_HOMO_) without a negative sign. LUMO → lowest unoccupied molecular orbital, which is directly related to electron affinity (EA = −*E*_LUMO_). Δ*E* → the energy gap (*E*_LUMO_ − *E*_HOMO_) or Δ*E* = (*I*_P_ − EA).37
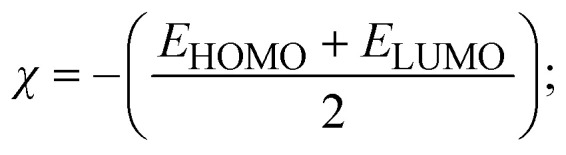
38
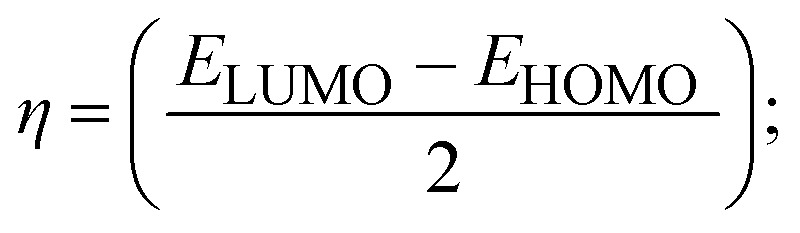
39
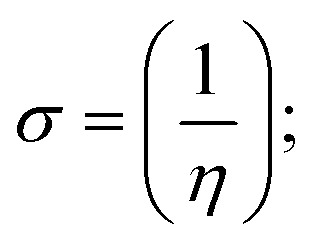

40 *μ*_i_ = –(*χ*);

41
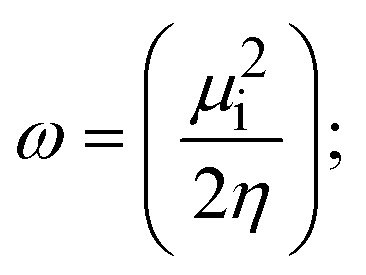
42
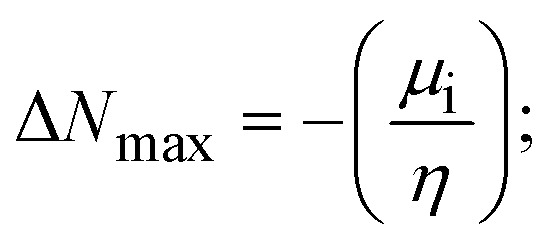
where *χ* → absolute electronegativity; *η* → absolute (global) hardness; *σ* → absolute (global) softness; *μ*_i_ → chemical potential; *ω* → global electrophilicity index; Δ*N*_max_ → additional electronic charge. *μ* → dipole moment (*μ* = *Q* × *r*) is the measure of net molecular polarity, which describes the charge separation in a molecule. It is the product of the charge *Q*, at the end of the molecular dipole, and the distance *r* between the charges, these parameters are effective in predicting global reactivity trends based on Koopmans' theorem.

Furthermore, a reduction in energy gaps is attributed to increased conductivity and solubility, which correlates with the shifting of the electronic absorption bands of metal complexes towards longer wavelengths (red shift) compared to the free ligand. In general, the band gap is determined by the strength of a donor's ability to donate electrons and the strength of an acceptor's ability to accept electrons.

Fig. 5 displays the FMOs of the free ligand (**HL**) and its complexes (1–3). The HOMO of **HL** covers the entire molecule, whereas the LUMO does not cover the morpholine ring (except the nitrogen atom). The HOMO of complex (2) is concentrated on the transition metal and the phenolic rings of the ligand (**HL**), while the LUMO covers the metal and the 2,2′-bipyridine moiety. However, in complexes (1) and (3), both the HOMO and LUMO demonstrate electron density concentration on the central metal, the phenolic rings, and the 2,2′-bipyridine unit. In particular, the central metal does not contribute to stabilizing these FMOs (Fig. S38† and Table 8). Besides frontier molecular orbital theory, conceptual DFT is another reactivity framework that provides the means to discuss the reactivity of molecular systems. CDFT relies on the assumption that the response of a system to an external perturbation gives access to measurable reactivity parameters.^109^ It is particularly useful when it comes to sorting related molecules in terms of reactivity trends. Table 8 collects the most common CDFT descriptors, namely the electronegativity (*χ*), the global hardness (*η*) and softness (*σ*), the chemical potential (*μ*_i_), the global electrophilicity index (*ω*), and the additional electronic charge (Δ*N*_max_), which were evaluated as per eqn (37)–(42) assuming the validity of Koopmans' theorem.^109^ Absolute electronegativity (*χ*) also indicates whether a substance is a Lewis acid or a Lewis base. High *χ* is ascribed to a Lewis acid, while low *χ* is ascribed to a Lewis base.^110^ As shown in Table 8, the observed *χ* for all substances were in the range 2.933–3.184. The same table also presents the dipole moments of all compounds, denoted by *D* to distinguish them from chemical potentials. Dipole moment values indicate that metal complexes (1–3) are an order of magnitude more polar than the ligand (**HL**) and should therefore be more soluble in water. Furthermore, global hardness denotes the propensity of a system to let its electron density be distorted by neighbouring molecules. It is admitted that the lower the hardness of a molecule (the higher its softness), the more pronounced its polarizability.^111^ Hence, it is clear from Table 8 that ligand (**HL**) is harder than each of the three complexes, which implies that the metal complexes are more polarizable than the free ligand (**HL**). In addition, electrophilicity indices suggest that the three metal complexes are more electrophilic than the free ligand. Put together, the CDFT data confirm the higher reactivity of the metal complexes compared to the free ligand and are thus expected to establish more stable complexes with various biomolecules through diverse binding modes.^112^Table 8 summarizes quantum chemical parameters (eV) or global reactivity descriptors, as well as the FMO energy gap and dipole moment values of the free ligand (**HL**) and metal complexes (1–3).

**Fig. 6:**
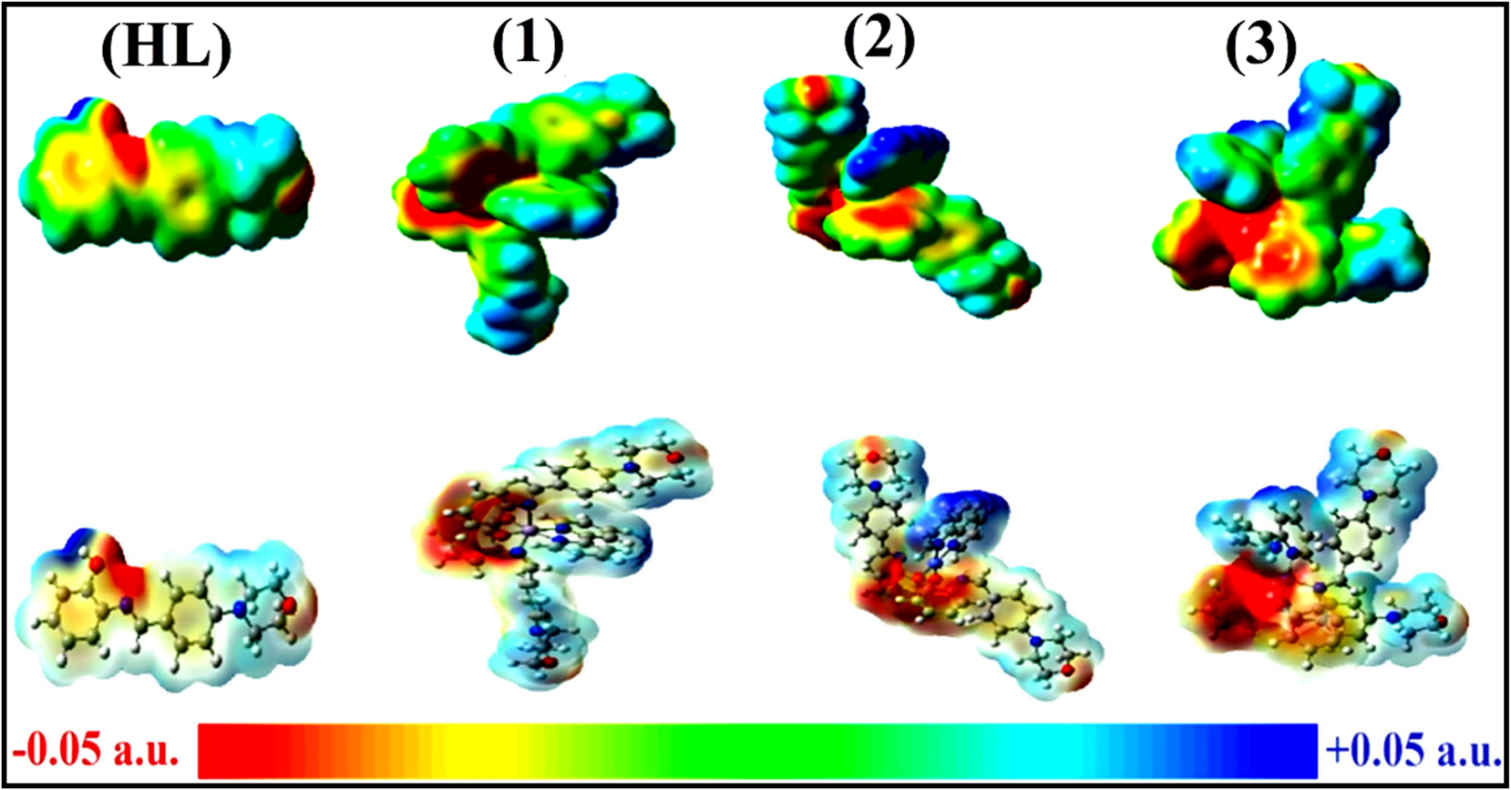
Molecular electrostatic potential (MEP) maps of the free ligand (**HL**) and associated complexes (1–3). Plots generated at the 0.002 isosurface value.

Although handy and practical for sorting molecules in terms of reactivity trends, global reactivity descriptors soon become useless when it comes to identifying the most reactive sites within a given molecule. This is where local reactivity descriptors come to the rescue. For instance, the electron density distribution of the HOMO and LUMO reveals that the most reactive non-metallic fragments of complexes (1–3) are the phenolic rings of (**HL**) and the bipyridine unit, which are then expected to engage in a variety of intermolecular interactions. Another well-established local reactivity descriptor is the molecular electrostatic potential (MEP). The MEP of a molecule is regarded as a measure of the force acting on a hypothetical positive charge due to the combined electrical effect of electrons and nuclei at a given position in the molecular spaces.^113^ Several studies have demonstrated the ability of the MEP analysis to accurately identify the most reactive sites involved in “hard–hard” interactions, in line with Pearson's hard–soft acid–base theory.^114^Fig. 6 displays MEP maps of (**HL**) and complexes (1–3) calculated at the 0.002 isosurfaces. The regions highlighted in red and blue in Fig. 6 are those where nucleophilic and electrophilic attacks are most likely to occur. Two nucleophilic sites are observed on the MEP of the free ligand. The first region surrounds both the phenolic O and the imine N atoms and mirrors the presence of the ESP global minimum of −46.6 kcal mol^−1^. The second spot is found in the nearest region of the morpholinic O atom and appears next to a local minimum of roughly −29.5 kcal mol^−1^. These findings show that the first site is more reactive than the second, which is also supported by the fact that the phenolic site is the one that binds to the central metal during the formation of the metal complexes. On the other hand, the MEP maps of complexes (1–3) present a negative electrostatic potential all over the phenolate units, whereas the bipyridine fragment carries a positive region enclosing all the H atoms fixed opposite to the N atoms. This observation corroborates, at least partly, the previous analysis of FMOs, highlighting that the phenolate and bipyridine fragments are the preferred binding sites for nucleophilic and electrophilic attacks, respectively. The free ligand (**HL**) and the complexes (1–3) both demonstrated amphoteric species that can function as Lewis acids and Lewis bases. Similar systems can be found throughout the literature.^115^

#### 3.4.2. Molecular docking properties with DNA/BSA/SARS-CoV-2 (3CL^Pro^)

The molecular docking method is a useful theoretical framework to realize metal complex–DNA interactions in rational drug discovery and development as well as in mechanistic analysis. It is also a valuable dynamic tool to estimate the relative binding affinities of the test compounds against validated therapeutic targets, which contributes to finding a biomolecule's preferred binding locations and improving comprehension of a drug's mechanism of action. The ligand (**HL**) and its complexes (1–3) were docked onto DNA/BSA/SARS-CoV-2 (3CL^Pro^) to figure out the best binding modes and the nature of interactions responsible for the stability of the complexes formed.

**Fig. 7:**
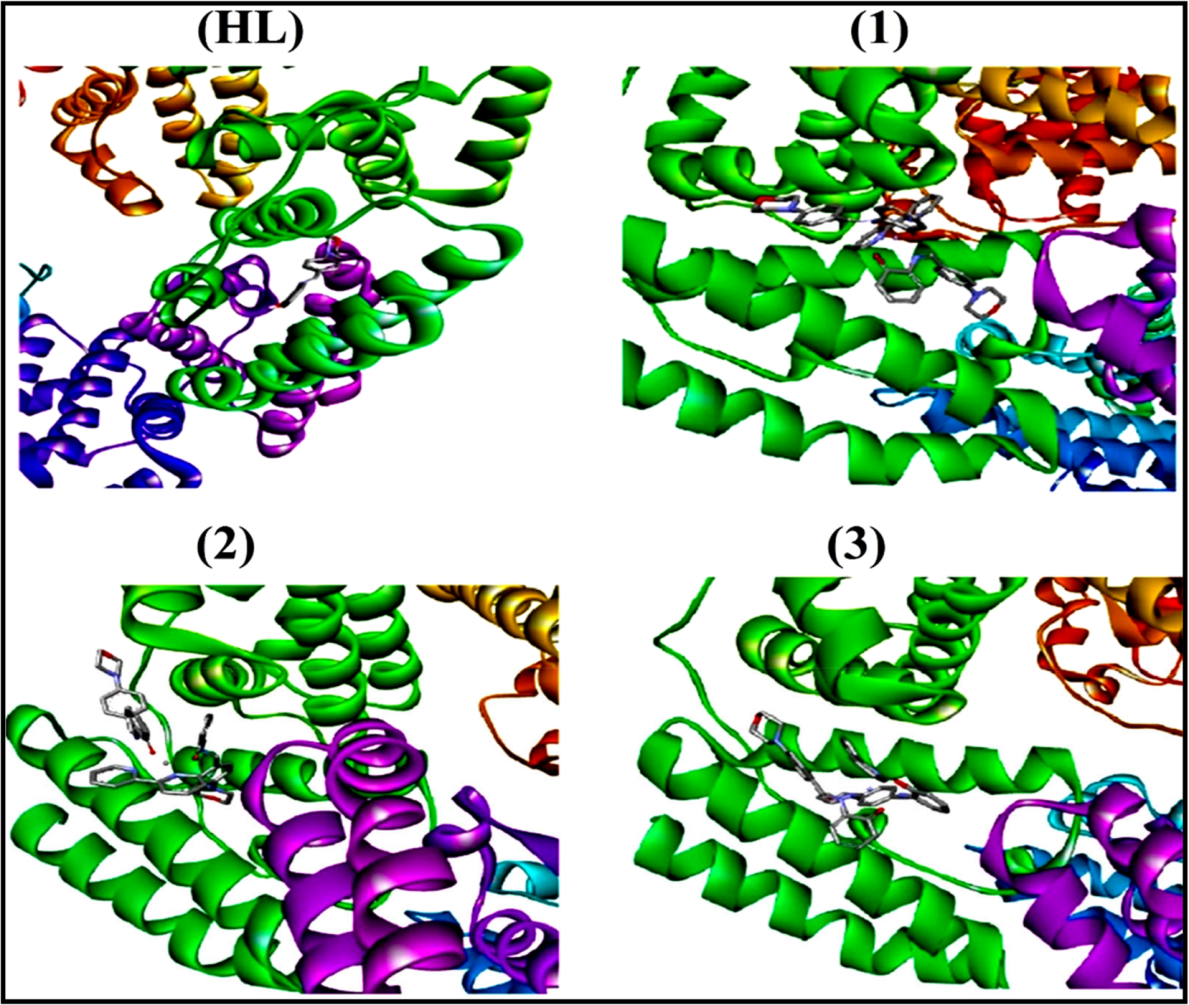
Best docking poses of guest molecules inside the active site of the BSA protein.

Fig. S39† illustrates the 3D models of the host biomolecules for BSA, CT-DNA, and 3CL^Pro^. The guest molecules of all test compounds were first docked inside the active site of BSA to measure their binding affinity and decipher the main interactions that ensure the stability of the resulting guest–host complex. Fig. 7 demonstrates the maximum docking positions. The observed binding energies were in the range of −8.2 to −10.1 kcal mol^−1^ and indicate the spontaneous formation of the guest–host complex. The observed binding affinities were found in the following sequence: −10.1 (2) > −9.3 (3) > −9.1 (1) > −8.2 (**HL**). It is imperative to take into account that metal complexes (1–3) have a persistent tendency to bind to BSA *via* the static mode. The stability of guest–host complexes does not only depend on their inherent reactivity but also on their size, conformation, permeability, polarity, and dipole moments, which are supported inside the active site. Also, note that guest molecules are maintained inside through several noncovalent interactions (Fig. S40†). The most notable ones are H-bonding interactions, π–π stacking, hydrophobicity, and van der Waals contacts, which are essential in complex binding. For instance, the free ligand (**HL**) forms two regular H-bonds with BSA, in which it acts as the proton acceptor. The phenolic O atom binds to Phe506 in the first interaction, which has a length of 2.73 Å, while the morpholinic O atom binds to the Asn504 amino acid residue in the second interaction. This finding supports the MEP's prediction of the local reactivity of the free ligand. Additionally, metal complex (1) is also engaged in conventional H-bonds with Lys563 and Val551, respectively, while complexes (2), and (3) do not form such interactions. The docking of our guest molecules inside 3CL^Pro^ was also favourable and demonstrated binding energies in the range of −6.7 to −9.3 kcal mol^−1^. The most stable guest–host complex was attributed to complex (1a), while ligand (**HL**) formed the least stable one. The observed binding affinities for all compounds were in the following sequence: −9.3 (2) > −8.2 (3) > −6.9 (1) > (**HL**) −6.7, indicating that metal complexes (1–3) have higher reactivity than the free ligand (**HL**). Furthermore, assessment of the relatively high negative binding energy of complex (2) indicates that the interactions with neighboring residues stabilize the metal complex through stronger interactions than complexes (1) and (3), and the sequence of binding interactions is consistent with their extended π-conjugation on the bridging ring.

**Fig. 8:**
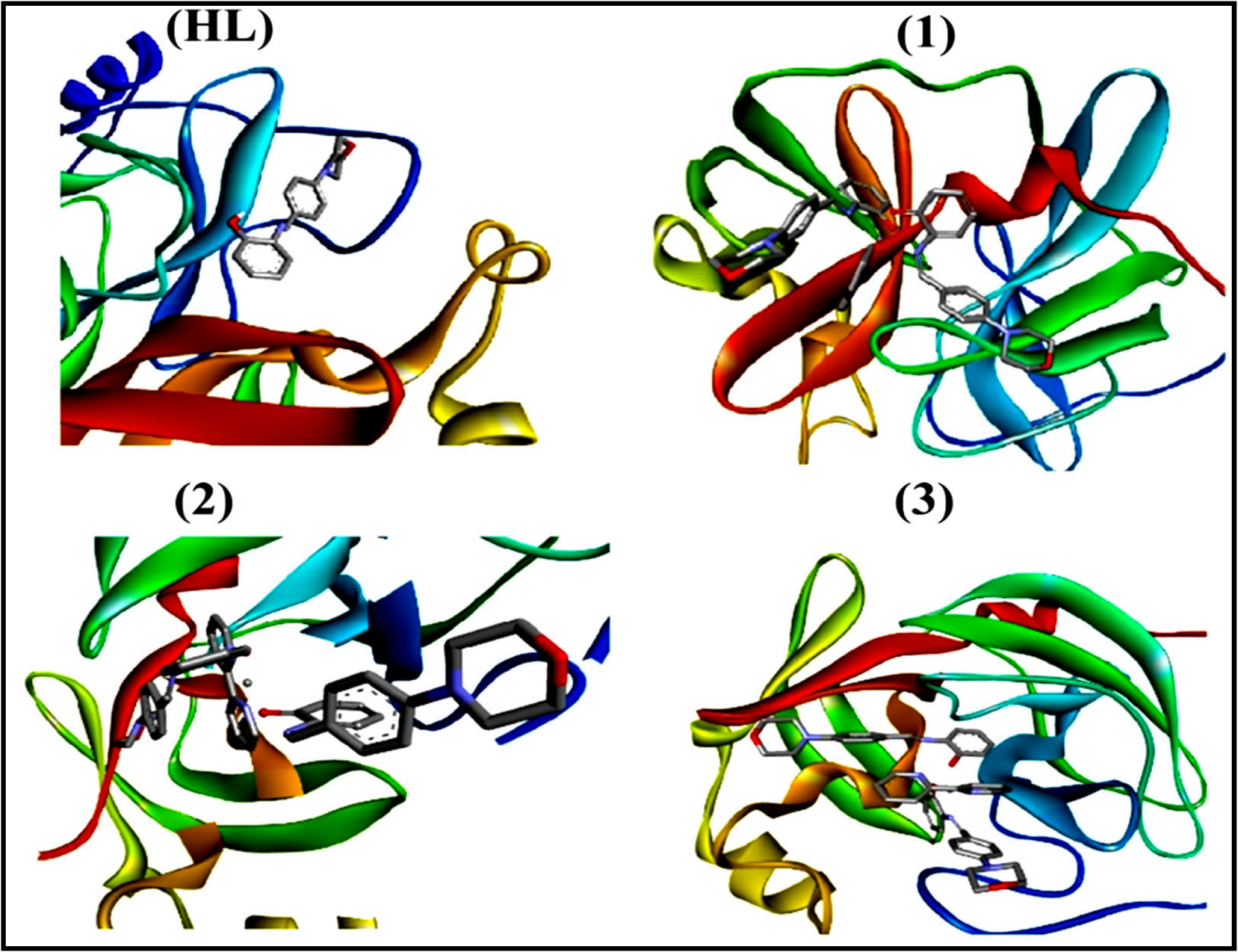
Best docking poses of guest molecules inside the active site of 3CL^Pro^.

**Fig. 9:**
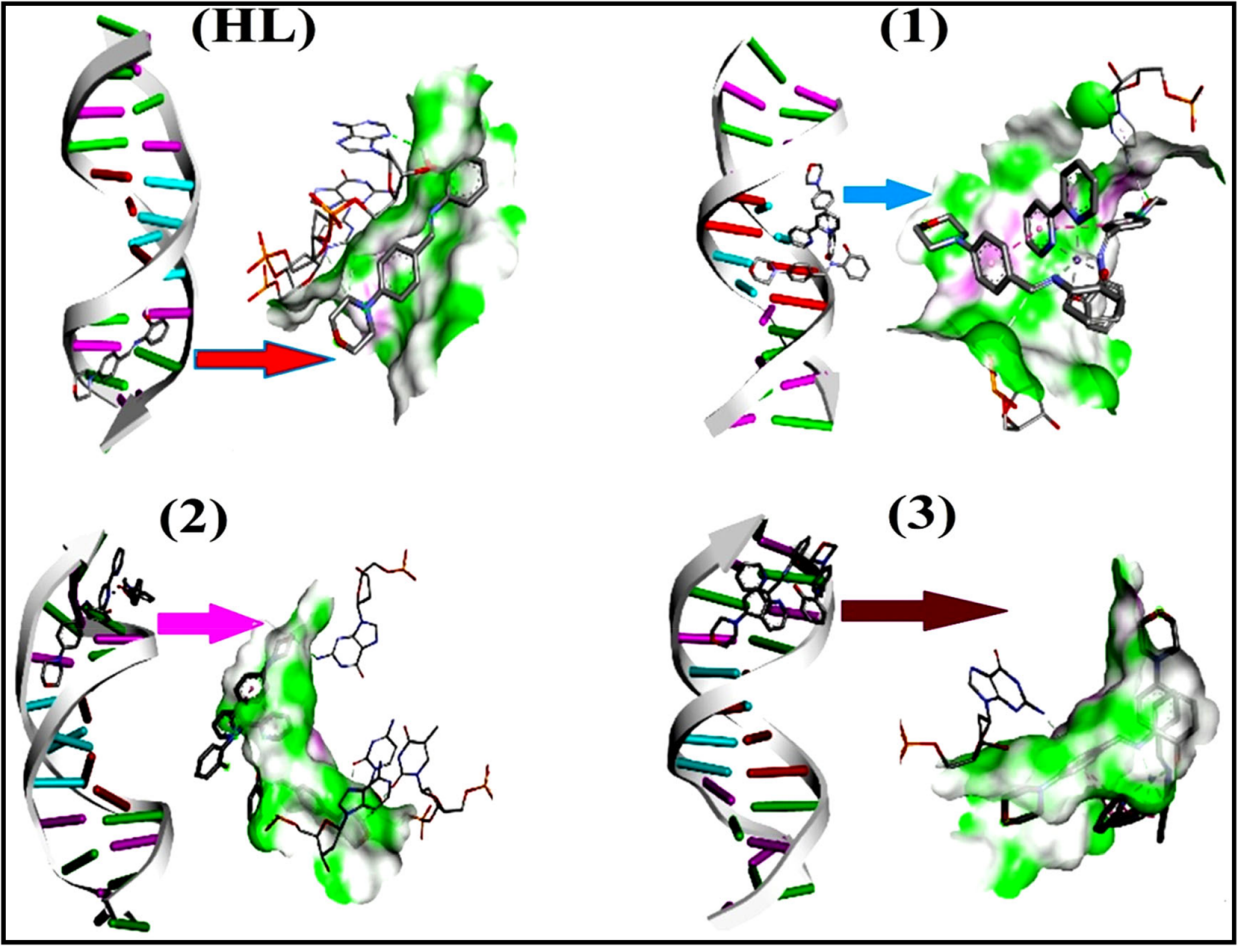
Best binding poses of our guest molecules in the CT-DNA double helix.

The best binding poses and the physical interactions that guest molecules establish inside 3CL^Pro^'s active site are shown in Fig. 8 and S41.† H-Bonds and π–π stacking interactions clearly contribute significantly to the stability of guest–host systems in ligands (**HL**) and complexes (2–3). Moreover, metal complexes (2–3) apart from complex (1) have potential binding ability compared to the co-crystallized ligand, which often functions as an optimistic control.^116,117^ The measured results encourage further *in vitro* studies to validate the inhibitory effect of the test compounds on the SARS-CoV-2 main protease. Furthermore, the binding energies on the CT-DNA double helix for all test compounds were in the range of −7.7 to −8.5 kcal mol^−1^ and followed this trend: −8.5 (2) > −8.2 (3) > −8.0 (1) > −7.7 (**HL**) kcal mol^−1^. As demonstrated in Fig. 9, all substances are effectively sandwiched between DNA double helixes *via* π–π stacking interactions, and hydrogen bonds including electrostatic forces enhance the overall binding ability. However, the results reveal that complexes (1–3) engage with the oxygen atom of the phosphate backbone of the deoxyribonucleic acid helix by an intercalation mode that involves exterior edge stacking. The docked molecules demonstrate that the enhanced planarity of the morpholine-linked ligand (**HL**) core permits sturdy π–π stacking interactions and that the complexes fit well into the intercalative in the DNA structure's guanine–cytosine-rich domain. Also, molecular planarity is one of the main factors for smooth penetration, which is enhanced by expanding the rigid π-surface of 2,2′-bipyridine on complexes. Thus, the complexes have revealed better binding abilities than the free ligand. Since metal complexes may be slightly affected by intercalative interaction due to their size and voluminousness (bulk) compared to the free ligand, they can also be stabilized along with electrostatic interactions and the formation of H-bonds. However, non-covalent π–π stacking interactions predominate when complexes (1–3) bind with DNA, and each metal complex within the DNA duplex is greatly stabilized as a result of these interactions as a whole. Finally, the modelling data reported above reasonably support the experimental results and provide additional details on the nature and extent of interactions between the considered biomolecules and our test compounds.

### 3.5. Assessment of antioxidant properties using UV-vis spectral titration

Any substance that is able to put off or reduce the oxidation of substrate (proteins/lipids/DNA/carbohydrates of living cells) or free radical formation is known as an antioxidant. Biological systems are shielded from the potential adverse effects of excessive oxidation by an oxidizable substrate. As a result, the free radical's energy may be reduced, radical generation suppressed, or the chain propagation of lipid oxidation may be stopped in the initial stages. They also donate hydrogen or electrons to the free radicals, turning them into nontoxic or H_2_O molecules.^118^ Of late, it has been found that antioxidant studies have attracted special attention among various biological studies due to their vital role in the execution of disorders associated with cancer. DPPH˙, OH˙, O_2_˙^−^, and NO˙ approaches have been utilized to measure the antioxidant activities of all test substances in terms of their proton-donating capability with electronic absorbance. These methods are frequently employed to evaluate a compound's ability to scavenge free radicals and the antioxidant activity of the targeted free ligand and metal complexes.^119–122^ The observed percentage of inhibition efficiency for all substances in terms of the IC_50_ findings for these assays is shown in Fig. S42a–d and Tables S12–S15.†

#### 3.5.1. Assessment of DPPH radical scavenging properties

An aqueous or methanol solution turns from dark purple to light yellow when DPPH, a stable chromogen free-radical, combines with an antioxidant molecule. Because DPPH quickly absorbs hydrogen or electrons from donor groups, a blank DPPH solution was used for baseline correction in the absence of a compound, and 517 nm (*ε* = 8320 M^−1^ cm^−1^) was observed to give a significant absorption maximum. It was found that when test compound concentrations (40–240 μM) increase, DPPH radical inhibition increases as well. The DPPH˙ radicals are reduced by an antioxidant compound (AH), in which the reduction of electronic absorbance for each compound was carefully noted at 517 nm.^123^ The capacity to obstruct radicals improves as the sample concentration increases. The assessed percentage of maximum inhibition for all substances was found at 240 μM in the following sequence: (**ascorbic acid**) (85.65) > (1) 62.23 > (3) 60.02 > (1) 58.05 > (**HL**) 52.45. The evaluated findings of IC_50_ for standard ascorbic acid and complex (2) were found at 80 μM and 200 μM, respectively (Fig. S42a and Table S12†). In this case, complex (2) demonstrated the best antioxidant potency compared to the others. Furthermore, the percentage of scavenging or maximum inhibition of all substances is estimated using the following eqn (43): scavenging (%) = [(*A*_0_ − *A*_S_)/*A*_0_] × 100, where *A*_0_ represents the absorbance of the control (DPPH alone in ethanol) and *A*_S_ denotes the absorbance of the sample (a mixture of DPPH and compounds in ethanol).

#### 3.5.2. Evaluation of hydroxyl radical inhibition

Hydrogen peroxide receives electrons *via* antioxidant molecules, and then they are neutralized into a water molecule. OH˙ inhibition capability was determined from the percentage of inhibition for all test substances at 230 nm. The maximum percentage of inhibition for all samples at 240 μM was observed in the following order: (2) 56.85 ≥ (1) 56.03 ≥ (3) 55.75 > (**HL**) 50.68. The standard ascorbic acid and complex (2) were found to have IC_50_ values of 160 μM and 200 μM, respectively. However, complexes (1–3) revealed similar antioxidant potency compared to the free ligand (Fig. S42b and Table S13†).

#### 3.5.3. Superoxide scavenging assay

Superoxide dismutase (SOD) is a vital catalytic enzyme in the human body's defense against free radicals, quickly and efficiently reducing toxicity and cellular damage by exchanging superoxide into water (or) harmless molecules. The percentage of inhibition for all substances was analyzed at 590 nm. The values were found in the following order: (**ascorbic acid**) 84.85 > (2) 62.24 > (3) 58.83 > (1) 54.12 > (**HL**) 50.42. However, complex (2) revealed the best antioxidant potency among them, and IC_50_ values for complexes (2–3) were found to be 200 μM (Fig. S42c and Table S14†).

#### 3.5.4. Assessment of nitric oxide inhibition

The diffusible nitric oxide free radical is a crucial chemical mediator that assists in overcoming diverse chronic human diseases. The NO˙ free radical scavenging potential for all test samples was also studied at 546 nm. The changes in electronic absorption intensity of the nitric oxide radical inhibition were monitored with respect to the sample concentration. When the test sample concentration rises, the nitric oxide free inhibition effectiveness also increases. The measured percentage of nitric oxide radical scavenging capability for all samples at 240 μM was obtained in the following order: (**ascorbic acid**) 72.73 > (2) 61.34 > (3) 59.65 > (1) 56.69 > (**HL**) 51.62. However, complex (2) showed superior antioxidant efficacy among the complexes (Fig. S42d and Table S15†).

### 3.6. Evaluation of antimicrobial properties

Current research has a curious focus on the *in vitro* antimicrobial properties of biological systems because these studies play a vital role in developing effective antibacterial and antifungal medications. The clear inhibition zone (mm) values obtained for various bacterial and fungal species in the samples are revealed in Fig. S43,† and the evaluated findings are summarized in Table 9. The results of the microbial activities revealed that the metal chelates demonstrated greater efficacy than ligand (**HL**) against the chosen bacterial and fungal pathogens, owing to the increased lipophilicity of the metal complexes under the same experimental conditions, and they accelerate the breakdown of the cell wall during biosynthesis in the microorganism enzymes as well as damaging the normal cell processes due to increasing the permeability of cells into lipid membranes.^124^ The obtained results suggest that all metal complexes demonstrate significantly greater antimicrobial properties than free ligand (**HL**) against a certain microorganism. They are contrasted with common medications like streptomycin and amikacin for treating bacteria, and ketoconazole and amphotericin B for treating fungi. Based on the chelation theory proposed by Overtone and Tweedy, it can also be elucidated that the partial exchanging of the positive charge of the metal center with donor groups and overlap of the ligand orbitals will reduce the greater degree of the metal ion's polarity, which ultimately leads to the delocalization of π and d electrons under the whole chelated ring system.^125^ By increasing the size of the metal ion due to retarding the polarization, chelation may also enhance the complexes' lipophilic characteristics, which further stimulates the lipid membrane permeability and breaks down the bacteria's enzymes responsible for cell wall formation, therefore slowing down the regular cell processes. Antimicrobial drugs frequently either fully eliminate microbes or prevent their cell growth by preventing the production of cell walls/proteins/DNA, including by obstructing folate metabolism and the cytoplasmic membrane. Additionally, the samples' mode of action may be employed in disrupting the cell's respiration process by the formation of H-bonds in the course of the morpholine-fused iminic group coordinated with the active metal center of its parts, inhibiting proliferation of the cell. The enhanced antibacterial activity could be attributed to changes in pharmacological kinetics, conductivity, steric and electronic effects, solubility, and metal–ligand bond length. The difference in the antimicrobial efficacy of some of the compounds towards various microorganisms depends on the impermeability of the cells of the germs or the diversity of ribosomes in the microbes.^126^eqn (43) is used to calculate the percentage of inhibition of all substances (Table 9). The cell walls of Gram-negative bacterial strains are generally composed of thick, multiple layers (20–80 nm) of peptidoglycan, which is more easily treated by antibiotics due to its 20–30% lipid content, whereas the cell walls of Gram-positive bacterial strains are composed of single, thin layers (8–10 nm) of peptidoglycan, which is more resistant to antibiotics due to its impermeable cell wall and very low lipid content. It is concluded that the present complexes exhibit significant activity against Gram-negative antibacterial strains compared to Gram-positive antibacterial strains. Antibacterial activity does not only depend on permeability and lipophilicity. It is also dependent on the structural properties of metal complexes, such as coordination behavior, ligand polarity and dipole moment, and the condensed central metal ion *via* charge equilibration, among other things.

**Table 9:** Investigation of the antimicrobial properties of all substances (measured as the diameter of the clear zone inhibition in mm) (inhibition%)

Compounds	Antibacterial activity							Antifungal activity		
	*A*	*B*	*C*	*D*	*E*	*F*	*G*	*H*	*I*	*J*
Ligand (**HL**)	09 (33)	09 (33)	11 (45)	08 (25)	14 (57)	09 (33)	09 (33)	10 (40)	10 (40)	11 (45)
Complex (1)	10 (40)	12 (50)	10 (40)	16 (63)	10 (40)	10 (40)	14 (57)	12 (50)	14 (57)	11 (45)
Complex (2)	10 (40)	10 (40)	10 (40)	13 (54)	09 (33)	11 (45)	13 (54)	12 (50)	12 (50)	11 (45)
Complex (3)	12 (50)	10 (40)	11 (45)	14 (57)	13 (54)	10 (40)	13 (54)	12 (50)	16 (63)	13 (54)
*Amikacin*	22 (73)	22 (73)	24 (75)	20 (70)	20 (70)	20 (70)	20 (70)	—	—	—
*Streptomycin*	24 (75)	26 (77)	24 (75)	21 (71)	25 (76)	21 (71)	21 (71)	—	—	—
*Ketoconazole*	—	—	—	—	—	—	—	16 (63)	18 (67)	18 (67)
*Amphotericin B*	—	—	—	—	—	—	—	15 (60)	17 (65)	17 (65)

*A*, *B*, *C*, *D* & *E* represent Gram-negative bacteria species *Escherichia coli*, *Salmonella enterica serovar typhi*, *Salmonella enterica serovar typhi*, *Pseudomonas aeruginosa*, and *Shigella flexneri*, respectively. *F* & *G* denote Gram-positive bacteria species *Staphylococcus Aureus* and *Bacillus cereu*. *H*, *I* & *J* represent fungal strains *Aspergillus niger*, *Candida albicans* and *Mucor indicus*. Standard drugs for bacterial strains: *amikacin* and *streptomycin.* Standard drugs for fungal strains: *ketoconazole* and *amphotericin B*. [Control (DMSO) = 6 mm].43
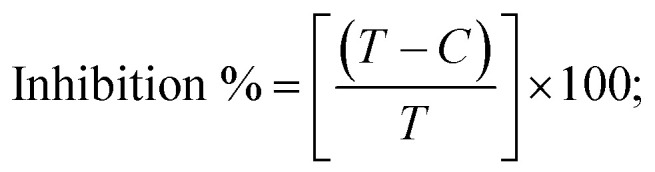
where *T* and *C* represent the diameter of microbial growth of the sample plates and the control plate (6 mm), respectively. Error limits ± 2.5–5.0% (*P* ≤ 0.05).

### 3.7. Evaluation of cytotoxic properties

Cellular viability or metabolic properties can be measured using the MTT assay, which is a powerful and consistent method for cytotoxic properties. The cytotoxic efficacy of all test compounds was assessed by the MTT assay against the A549, HepG2, MCF-7, and NHDF cell lines.^127^ As per the colorimetric approach, the IC_50_ values of all compounds were evaluated *via* the percentage of cell viability or inhibition of growth.^128^ Even though the complexes show higher activity compared to the ligand (**HL**) towards various cancer cell lines, the NHDF cell line is only mildly perturbed compared to cisplatin. However, complexes (2) and (3) were exposed to similar cytotoxic potential as the others,^129^ which induces apoptosis due to their ability to produce ROS more efficiently than the free ligand. The acquired findings were in the following sequence: (**cisplatin**) > (2) ≈ (3) > (1) > (**HL**) (Fig. S44† and Table 10). The cytotoxic effectiveness is dependent upon the DNA binding modalities, the structure–activity relationship, the drug concentrations, and the incubation period exposure.^130^ The results also suggest that complexes (2–3) may be potential candidates for future chemotherapies. In addition, it is suggested that these complexes consist of morpholine-fused primary aromatic and secondary 2,2′-bipyridine planar systems connected with a metal center, which facilitates their simple insertion within the base pairs of DNA. Coordination between the ligands and metal ions results in charge equilibration, which diminishes the polarity of the metal ions and also causes the ability of the test complexes to pass *via* the cell membrane lipid layer in accordance with Tweedy's chelation theory. Thus, it inhibits the synthesis of cell-wall/protein/nucleic acid. The measured percentage of growth inhibition for these compounds is summarized in Table 10. Additionally, the results of DNA binding tests using these complexes, including gel electrophoresis, UV-vis spectral titration, hydrodynamics, emission, and CV findings, were in good agreement with the findings of cytotoxicity.^131^ Expressions eqn (44) and (45) were applied to measure the percentage of growth inhibition and cell viability (Table 10). In this case, complexes (2) and (3) have been proven to have greater biological efficiency than the others. Cytotoxicity depends on not only the hydrophilicity or lipophilicity and permeability, which only assist the crossing of the cell membrane but are not responsible for cell death, but also on several other factors, including the Lewis acid character, overall stability, solubility, conductivity, compatibility, length of the metal–ligand bond, overall charge, electron density, reduction potential, dipole moment, intermolecular hydrogen bonds, proton transfer equilibrium, and coordination environment. These significant elements might also contribute to the increased biological activity. Moreover, cobalt is also generally not considered to be a very toxic element, and its coordination complexes have fascinating redox and magnetic characteristics that fit them for a remarkable range of biological and medical uses. Hence, cobalt complexes have diverse potential to be used as medications due to their low-energy d–d transitions and MLCT bands, which also enable the regulation of therapeutic activity as well as antibacterial, anticancer, and antiviral activities, which are well documented.^132^ Moreover, it is possible to intelligently adjust these factors to successfully target a desired pathway or biomolecule. Designing such well-controlled and selective cobalt-based therapeutics may benefit from a mechanistic understanding of the varied bioactivities of cobalt complexes. Thus, knowing the mechanism of action can direct the advancement of prospective cobalt treatments to clinical trial.^133^

**Table 10:** The evaluation of the cytotoxic properties of ligand (**HL**) and its complexes (1–3) against A549, HepG2, MCF-7 and NHDF cell lines

Compounds	IC_50_^a^ (μM) (SI)^b^			
	A549	HepG2	MCF-7	NHDF
**Cisplatin**	31.9 ± 1.6 (0.84)	22.9 ± 1.1 (1.17)	20.2 ± 1.0 (1.33)	26.9 ± 1.3
(**HL**)	126.4 ± 6.3 (1.65)	108.4 ± 5.4 (1.92)	105.2 ± 5.3 (1.98)	208.6 ± 10.4
(1)	37.6 ± 1.9 (2.03)	36.0 ± 1.8 (2.13)	36.6 ± 1.8 (2.09)	76.6 ± 3.8
(2)	34.2 ± 1.7 (2.16)	35.9 ± 1.8 (2.06)	35.9 ± 1.8 (2.06)	74.0 ± 3.7
(3)	33.5 ± 1.7 (2.20)	33.6 ± 1.7 (2.19)	34.5 ± 1.7 (2.13)	73.8 ± 3.7

a Average IC_50_ values from at least three independent experiments for drug concentration (μM) of 50% cell death following 72 h of exposure. A549, HepG2, MCF-7 and NHDF are human lung cancer cell line, liver cancer cell line, breast cancer cell line and normal human dermal fibroblasts cell line.

b Selectivity Index (SI) = (IC_50_ value of normal cells/IC_50_ value of cancer cells).



44

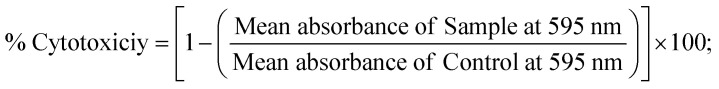

45 Cell viability (%) = [100 − Cytotoxiciy(%)];

46

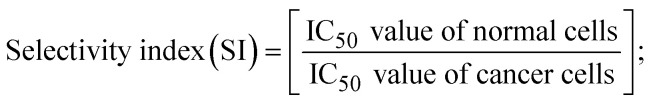

Error limits ± 2.5–5.0% (*P* ≤ 0.05).

Moreover, the selectivity index (SI) value is a very important factor for observing the toxicity of drugs, which is also measured from the ratio of a sample's toxic concentration to its effective bioactive concentration using eqn (46) (Table 10). The SI values are mostly observed to be between 1 and 10. Also, several authors have suggested that a criterion for the approval of good drugs or a selective bioactive sample is that it should have SI ≥ 10, which means that the ideal drug should have a relatively high toxic concentration with a very low bioactive concentration.^134–137^ Generally, SI < 1 indicates that the sample could be toxic and cannot be treated as a drug, furthermore, according to the concepts of Nogueira and Estólio do Rosário, the SI value should not be lower than 2, while SI < 2 is thought to show discriminating toxicity and could even damage healthy cells. On the other hand, SI > 2 pronounced higher selective toxicity against cancer cells.^138^ In this case, the observed SI values of the present complexes (1–3) against A549, HepG2 and MCF-7 cancer cell lines were greater than 2, except for the free ligand (Table 10) and cisplatin, which also had a smaller SI value (SI < 2), which indicates greater toxicity than the others. Hence, we concluded that further research into these complexes might be conducted to produce more potent and targeted chemotherapeutic treatments against cancer cells.

### 3.8. The shake flask method for calculating partition coefficient (log *P*_o/w_)

Lipophilicity is an imperative factor in anticancer and antimicrobial properties, particularly the necessity for a relevant hydrophobicity to assist the crossing of the cell membrane. This is also supported for the characterization of a drug because it has a fundamental impact on the pharmacokinetic profile and is associated with its capacity to enter tumor cells through passive diffusion.^139^ Also, they have been demonstrated to associate with biological activity measurements in a highly diverse range of experimental settings, from straightforward protein binding to *in vivo* impacts on animals and humans. This is probably due to the fact that hydrophobic effects play a crucial role not only in the intramolecular interactions that take place between a medication and its target site, but also in the distribution of a drug within a biosystem, its interaction with rival binding sites, passage across and into membranes, and its interaction with metabolizing enzymes. However, the “accurate” model system for hydrophobic effects is octanol/water. Hydrophobicity (lipophilicity) is associated with the partition coefficient (log *P*), which is a measure of the ability of the solute to go into two immiscible phases (polar and non-polar environments). Also, it refers to the concentration of unionized species of compounds. Generally, the ability of a drug to penetrate with high permeability into lipid membranes depends on the solubility and partition coefficient of the drug. Furthermore, the partition coefficient is extensively employed in the discovery of new medications, chromatography, physical chemistry, environmental science and other fields. Since lipophilicity is one of the major parameters affecting important biological processes, it can influence drug intake *via* the absorption, distribution, metabolism, excretion, and toxicity (ADMET) characteristics of the substances.^140^ Additionally, the ability of a molecule or drug to passively diffuse across cell membranes has been linked to its lipophilicity, which is a measure of the solubility of the substance in aqueous and lipid-like environments.^141^ In general, chemotherapeutic medications often need to have certain solubility in the lipid phase in order to be absorbed by the biofilm. Meanwhile, the intracellular milieu is a water-soluble environment once the drug has entered the cell. The medicine must be water-soluble for it to have any anticancer effects. More remarkably, numerous investigations have demonstrated that log *P* is crucial for the intracellular absorption and sub-cellular localization of chemotherapeutic medication, which influences the anticancer activity.^142^ Conversely, the cytotoxic properties of a synthesized complex are not only associated with its DNA/protein binding affinity but may be also influenced by its lipophilicity. Additionally, molecules with higher lipophilicity have increased permeability into cell membranes, which determines a drug's cytotoxicity. A low-lipophilicity molecule typically exhibits poorer permeability. However, more positive partition coefficient values (log *P* > 0) correspond to higher lipophilicity, and more negative values (log *P* < 0) correspond to higher hydrophilicity. Generally, log *P* is measured between −3 and +10; if log *P* > −3, the substance has an extremely hydrophilic character, and if log *P* > +10, the substance has an extremely hydrophobic character. In addition, the aromatic ligands get larger in size and become more hydrophobic. When moving from ligands to complexes, the log *P* value increases significantly in comparison to ligands. The present complexes (1–3) have higher values of log *P* and log *D* compared to the free ligand (Table 11). However, when log *P* > ±2, the drug will be harder to excrete from the body due to its high lipophilic character, which also creates toxicity due to accumulation. This is determined by the logarithm of the partition coefficient (log *P*_o/w_) in the *n*-octanol/water system for all compounds with the support of eqn (47) and the findings were also compared with cisplatin (log *P* = −2.28 ± 0.07)^143^ (Table 11). When the partition coefficient is less than ±2 (log *P* ≲ ±2), the drug exhibits good penetration without accumulation in the body. The overall observed log *P* values for the following compounds were: −2.280 (**cisplatin**), +1.087 (**HL**), +1.166 (1), −1.477 (2), and +1.170 (3) with error limits of (±0.05) (Fig. S45–S47† and Table 11). In this case, complex (2) exhibited both high hydrophilic and low hydrophobic characteristics in relation to cisplatin and other compounds that exhibit lipophilicity. The observed results are also well correlated with cytotoxic properties. Moreover, in an equilibrium two-component system, the partition coefficient (log *P*) reflects the ratio of neutral solute concentrations in the organic and aqueous phases. As a result, log *P* expresses the inherent lipophilicity of a compound in the absence of dissociation or ionization. However, the distribution coefficient is the overall ratio of a chemical substance between the two phases: the ionized and unionized fractions (log *D*).^144^ This expression refers to the effective or net lipophilicity of a compound at a specific pH, taking into consideration both the intrinsic lipophilicity of the compound and the degree of ionization. Log *P*eqn (47) and log *D*eqn (48) are interconnected for monoprotic organic acids through eqn (49) and for monoprotic organic bases through eqn (50). Also, pH and p*K*_a_ findings were measured using the Henderson–Hasselbalch eqn (51) (Table 11). In addition, the partition coefficient is independent of pH. But the distribution coefficient (*D*) is a pH-dependent factor, because it depends on every species in the organic and aqueous phases (neutral and ionized). Thus, the drug molecule must be unionized in an aqueous solution.^145,146^

**Table 11:** Correlation between partition coefficient (log *P*) and distribution coefficient (log *D*) *vs.* BSA binding constants (*K*_b_) & Gibb's free energy change 
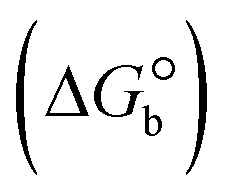
 for complexes (1–3) and free ligand (**HL**)

Compounds	Concentrations (M)			Log *P*	Log *D*	Net pH (p*K*_a_)	*K*_b_ × 10^4^ M^−1^	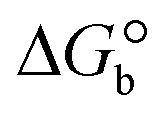 (kJ mol^−1^)
	Organic layer (M)	Aqueous layer (M)	Aqueous ions (M)					
(**HL**)	11.0 × 10^−3^	0.90 × 10^−3^	7.89 × 10^−4^	1.087	0.814	3.35 (3.08)	0.7556	−22.1246
(1)	23.5 × 10^−3^	1.60 × 10^−3^	2.38 × 10^−4^	1.166	1.107	3.48 (3.42)	1.2429	−23.3580
(2)	0.40 × 10^−3^	12.0 × 10^−3^	5.66 × 10^−5^	−1.477	−1.497	4.38 (4.36)	3.0937	−25.6174
(3)	18.5 × 10^−3^	1.25 × 10^−3^	3.57 × 10^−5^	1.170	1.158	4.76 (4.75)	1.8374	−24.3265

Partition coefficient (log *P*_o/w_) and distribution coefficient (log *D*_o/w_) values for all compounds determined using organic (*n*-octanol) and aqueous (deionised water) phase. Binding constants (*K*_b_) and Gibb's free energy change 
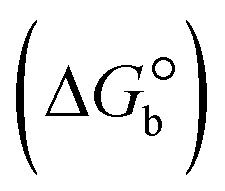
water phase values were collected from UV-visible titration for all substances bound to BSA. Log *P*_o/w_ values of a good drug should be in the range of (1–2). Log *P*_o/w_ > −3 indicates an extremely hydrophilic character, while log *P*_o/w_ > +3 indicates a highly lipophilic character. The observed results were compared with the standard anticancer drug cisplatin (CP) (log *P* = −2.28 ± 0.07).47
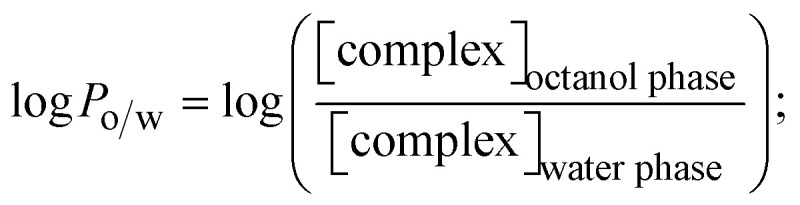
48
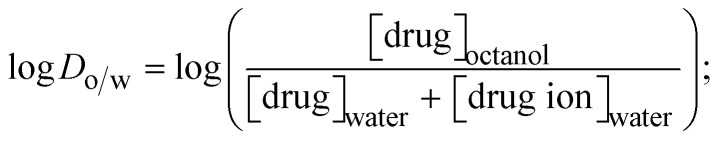

49 log *D* = log *P* – log(1 + 10^pH−p*K*_a_^);

50 log *D* = log *P* – log(1 + 10^p*K*_a_−pH^);

51
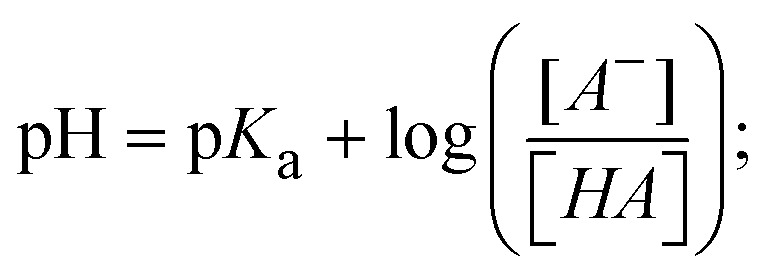
where [*A*^−^] and [*HA*] represent the concentrations of conjugate base and acid. *K*_a_ is the acid dissociation constant. When p*K*_a_ = pH, there are equal amounts of protonated and deprotonated forms of the compound under equilibrium conditions due to the ratio of concentration of conjugate base and acid being unity and it also follows the sequence: −1 < log([*A*^−^]/[*HA*]) > 1; when p*K*_a_ > pH, there is the possibility of protonation. When p*K*_a_ < pH, there is the possibility of deprotonation. pH = 7.11 and 6.00 are used for deionised water and *n*-octanol (net pH = 5.97); overall error limits ± 5.0% (*P* ≤ 0.05).

However, every part of a drug molecule imparts hydrophobic or hydrophilic characteristics to the entire molecule. Furthermore, higher cytotoxic activity of the complexes correlates with higher cellular absorption. Despite this, the findings generally imply that log *P*_o/w_ values might serve as helpful predictors of cytotoxicity for all test substances (Fig. S47†). Additionally, metal complex cytotoxicity is connected with certain chemical characteristics, such as binding constants for their interaction of small molecules with biomolecules (DNA/BSA) or their hydrophobicity and ability to traverse lipid bilayers (Fig. S46†).^147,148^ Many research reports suggest that there is a correlation between the hydrophobicity of bipyridine/phenanthroline based metal complexes and their level of cytotoxicity.^149,150^ In this case, the high cytotoxicity of complex (2) is mainly correlated with a moderate hydrophobicity, which has negative values (−1.477) of the partition coefficient like the standard anticancer drug cisplatin (−2.280) (Tables 10 and 11). On the other hand, it was also observed that the higher hydrophobicity of complex (3) exhibited a significantly diminished cytotoxic efficacy. Furthermore, because the cytotoxicity activity (IC_50_) is directly related to the partition coefficients of a substance, some factors such as π–π stacking and electrostatic forces, including the formation of hydrogen bonds, were suggested to cause cytotoxicity, as well as this trend, which presumably results from the cooperative effect of hydrophobic and electrostatic interactions. Furthermore, it is clear that increasing cytotoxicity is dependent not only on hydrophobicity, permeability (membrane penetration), solubility, absorption, plasma protein binding, and distribution, but also on π–π stacking and electrostatic forces. Also, the coordination behavior, the polarity and dipole moment of the ligand, and the reduced core metal ion *via* charge equilibration are possible explanations for the complicated ability of cells to pass through the lipid layer of the cell membrane.^151^ In particular, proteins containing a number of charged amino acids with negative phosphate residues exhibit higher electrostatic interactions with complexes possessing lower hydrophobicity, leading to greater cytotoxic effects.^152^

## 4. Conclusion

All compounds were treated with diverse analytical, spectral, and X-ray diffraction analyses. The observed results for complexes (1–3) suggested an octahedral geometry. The gel electrophoresis results showed that complex (2) displayed excellent metallo nuclease efficacy. The overall DNA binding properties of all compounds reveal that complexes (1–3) could bind with DNA through intercalation, which was further confirmed by their biothermodyanamic properties. The observed BSA binding constants of all samples pointed toward the possibility that the complexes could bind with BSA in the static mode, which was further supported by FRET measurements. Complex (2) also had the highest DNA/BSA binding affinities among them. The electronic configuration data for these substances was observed from DFT computations and their molecular docking studies on the interacting affinity of these substances against DNA/BSA/SARS-CoV-2. The computational findings also demonstrated that the metal complexes bind spontaneously inside the active sites of these biomolecules. Also, the enhanced reactivity of the metal complexes with the ligand is well accounted for in the context of FMO theory. The overall theoretical measurements for all substances were reported to be in excellent accord with the experimental results. The antimicrobial properties revealed that the metal complexes have significantly higher inhibition potency than the free ligands (**HL**). The scavenging properties put forward by complexes (1–3) stood out as having greater potential to scavenge radicals than the free ligand. The observed *in vitro* anti-cancer properties for all the substances, including the standard drug cisplatin (**CP**), revealed that complexes (2–3) demonstrated the best cytotoxic efficiency among them, with less influence on normal cells than cisplatin. Additionally, the partition coefficient (log *P*) values of all test compounds showed good correlation with cytotoxic activity (IC_50_) and complexes (2–3) might function as a new class of anticancer agent in the future.

## Conflicts of interest

The authors declare that there are no conflicts of interest in this work.

## Supplementary Material

MD-014-D2MD00394E-s001

MD-014-D2MD00394E-s002
