# Supplementary material for: Exploring the antioxidant, antimicrobial, cytotoxic and biothermodynamic properties of novel morpholine derivative bioactive Mn(ii), Co(ii) and Ni(ii) complexes – combined experimental and theoretical measurements towards DNA/BSA/SARS-CoV-2 3CLPro
Source: RSC Med Chem. 2023 Jan 3;14(9):1667–97. doi: 10.1039/d2md00394e (PMC10508264; doi:10.1039/d2md00394e)
Supplement: MD-014-D2MD00394E-s001 [file MD-014-D2MD00394E-s001.pdf]

Electronic Supplementary Material (ESI) for RSC Medicinal Chemistry.
This journal is © The Royal Society of Chemistry 2022

Electronic Supplementary Information File

Exploring the antioxidant, antimicrobial, cytotoxic and biothermodynamic properties of novel morpholine derivative bio-active Mn(II), Co(II) and Ni(II) complexes - A combined experimental and theoretical measurements towards DNA/BSA/SARS-CoV-2 3CL^{pro}

Karunganathan Sakthikumar [a], Bienfait Kabuyaya Isamura [a,c] and Rui Werner Maçedo Krause [a, b] *

[a] *Department of Chemistry, Center for Chemico- and Biomedicinal Research (CCBR), Faculty of Science, Rhodes University, Grahamstown 6140, Eastern Cape, South Africa*

[b] *Center for Chemico- and Biomedicinal Research (CCBR), Faculty of Science, Rhodes University, Grahamstown 6140, Eastern Cape, South Africa*

[c] *Department of Chemistry, The University of Manchester, Manchester M13 9PL, Great Britain*

* *Correspondence should be addressed to Rui Werner Maçedo Krause: r.krause@ru.ac.za*

* Corresponding author:

Prof. Rui WM Krause,

Professor, Organic & Medicinal Chemistry,

Department of Chemistry, Faculty of Science, Rhodes University, Grahamstown 6140,

Eastern Cape, South Africa.

Email: r.krause@ru.ac.za,

Phone: +27 741622674; +27 46 603 7030

2. Experimental section

2.1. Materials and techniques

All chemicals, reagents, and solvents of analytical-grade were procured from Sigma-Aldrich, BD Biosciences, and Alfa Aesar's repositories. The ligand (**HL**) and its mixed ligand complexes (**1–3**) were examined by a variety of analytical and spectroscopic studies. The full details were summarized in our earlier reports.[21, 13]

2.2. Assessment of DNA/BSA binding features

2.2.1. Assessment of DNA nuclease efficacy

All substances were examined for DNA cleavage ability and the characteristics were examined for all substances along with DNA by gel electrophoresis approach under H_2O_2 in Tris-HCl buffer solution with a pH of 7.4.[21, 13] The characteristics were examined of all substances along with DNA containing H_2O_2 and Tris-HCl buffer solution with a pH of 7.4.[21, 13] The gel layer was removed from the tank solution after the experiment was finished and put in front of a UV transilluminator. Additionally, each band lane was scrutinized with control (DNA+H_2O_2).[22, 13]

2.2.2. Analysis of DNA-interaction characteristics

The DNA-binding experiment was carried out by an electronic absorption spectrophotometer by raising the DNA concentration from zero to 50 μM to the given concentration of all samples (50 μM) in Tris-HCl buffer (5mM Tris-HCl/50 mM NaCl) with a pH of 7.4 at 25° C. Concentration of DNA was also verified spectrophotometrically with an extinction coefficient of 6,600 M^{-1} cm^{-1} at 260 nm.[23–26]. Additional information was summarized in our prior report.[13]

2.2.3. Assessment of biothermodynamic characteristics

The deoxyribonucleic acid thermal denaturation properties were performed by an electronic absorption spectrophotometer connected to a temperature-controlled sample container in both the presence and absence of the substances. In a 5mM Tris-HCl/50mM NaCl buffer solution with a pH of 7.4, CT-DNA was treated with all test substances in a 1:1 ratio (50 μM). The experiments were executed by observing the absorption (260 nm) of CT DNA at different temperatures between 25 and 100 °C in the absence and presence of the complexes.[27–29, 13]

2.2.4. Assessment of DNA affinity by hydrodynamic technique

Hydrodynamic properties were performed using an Ostwald viscometer with the help of a thermostat (25 ± 0.1 °C). The experiment was performed of all samples, including ethidium bromide at various concentrations (20, 40, 60, 80, 100 μM) and each test compound was mixed with the deoxyribonucleic acid solution (100 μM) in the Ostwald viscometer. With a help of digital stopwatch, average flow time was gauged three times for each addition, and the specific viscosity of deoxyribonucleic acid was also measured in the presence and absence of test samples.[13, 30]

2.2.5. Assessment of DNA/BSA binding characteristics by a fluorometeric *technique*

In the emission spectral titration, ethidium bromide (EB) ($C_{21}H_{20}BrN_3$) solution (20 μM) in Tris-HCl buffer solution was incubated with the DNA solution (200 μM) with saturated binding levels and kept in a dark place for two hours. The titration was performed for all tested compounds (1–200 μM) with pre-incubated EB-bound deoxyribonucleic acid and carefully monitored the intensity variations between 510 nm and 610 nm in the presence and absence of deoxyribonucleic acid during the initial emission and excitation of EB.[31] Also, the emission spectral titration for all compounds was also carried out at a fixed concentration (25 μM) of BSA with an incremental concentration of substances (0–25 μM)

in a Tris-HCl buffer solution with a pH of 7.4 and the binding ability of all samples with BSA was examined at a fixed excitation wavelength of 278 nm and perceived the emission at 350 nm.[32] Concentration of BSA was also confirmed spectrophotometrically with an extinction coefficient of 43,824 M^{-1} cm^{-1} at 278 nm. Additional data was included in our prior report.[13]

4.2.6. Förster's theory-based FRET computation

The intersection of the acceptor's (compound) absorption spectrum and the donor's (BSA) emission spectrum describes that the acceptor group may get excited as a result of the excited donor's energy being lost to the ground state. Resonance refers to the phenomenon of matching energy.[33–35] According to Förster's theory, the critical distance between donor and acceptor molecules can be estimated using the FRET approach to assess the binding affinity between BSA-substance systems, and full details of other FRET parameters with their representations was also included in the previous report.[13]

2.2.7. Analysis of DNA binding characteristics using the CV method

The electrochemical analysis is a critical approach to examining the electron transfer reactions of the test compounds. With a scan rate of 0.1V, the electrochemical analyzer (CHI 620C) was utilized with a three-electrode system consisting of glassy carbon as the working electrode, Ag/AgCl as the reference electrode, and platinum wire as the counter/auxiliary electrode to observe the changes in peak current as well as peak potential under the interaction between test compound and deoxyribonucleic acid. The CV analysis for free substances was performed at 10 μM at 25 °C in a 5mM Tris-HCl buffer solution with a pH of 7.4. While CT-DNA increases (0–10 μM) in each sample solution, shifts in potential, including variations in the anodic and cathodic peak currents, have been monitored and the changes in peak current as well as peak potential further afford data on

the binding constant as well as the mode of interaction for test compound–DNA adducts.[36,13]

2.2.8. Assessment of BSA binding characteristics by electronic absorption titration

The absorption titrations were done with a 25 µM concentration of BSA at 25°C in a Tris-HCl buffer solution with a pH of 7.4. While the sample concentrations (0–25 µM) increased in the same BSA concentration solution, the change in the absorption band at 278 nm was continuously measured. The BSA property is further supported by the evaluation of a strong absorption band at 278 nm in the UV region. The increment of the test compound causes a slow rise in the absorption band of the BSA, which leads to the hyperchromism effect with hypsochromic shift, indicating that alterations in conformation happened owing to non-covalent interactions between the test compound and bovine serum albumin.[37, 13]

2.3. DFT and molecular docking simulations

To validate the results found from the experimental studies, the synthesized compounds were further investigated for their interaction with DNA/BSA/SARS-CoV-2 $3CL^{pro}$. All test compounds were fully optimized with the help of the hybrid B3LYP functional as accomplished in the Gaussian 09 package.[38] To demonstrate the global and local reactivity of all substances, the FMO theory[39] and molecular electrostatic potentials[40] were studied. Using the B3LYP-optimized structures of each substance, docking computations were also carried out. The Autodock Vina software was used for input structure preparation and calculations[41] and the visualization was performed on Discovery Studio.[42]

2.4. UV-Vis absorption titrations for in vitro antioxidant assay

All samples were evaluated for their scavenging abilities using the UV-Vis absorption titrations at different concentrations of 40, 80, 120, 160, 200, and 240 μM. While performing the antioxidant properties for the DPPH$^{\square}$, OH$^{\square}$, $SO_2^{\square-}$, and NO$^{\square}$ radical scavenging, the absorbance at 517, 230, 590, and 546 nm, respectively, was closely observed. The observed IC_{50} values of all samples were further compared with standard ascorbic acid.[43, 44, 13]

2.5. Assessment of in-vitro antimicrobial properties

In-vitro antimicrobial properties were evaluated for all samples by the agar disc diffusion method towards some selected fungal and bacterial strains.[45–47] All the microbial strains were grown for 24 h at 37° C with 200 rpm agitation in Mueller Hinton broth (MHB) and they were treated with 20 μL of test substances (100 μM) and incubated for 48 h for bacteria and 7 days for fungal strains. It was continuously monitored for the diffusion of chemical substances into the agar medium and inhibition of the growth of the microorganism. Also, the diameter of clear zone inhibition was assessed in millimeter with the help of a ruler under the plate's base. Additionally, the observed inhibition zone values were comparatively analyzed with standard antifungal drugs *ketoconazole* and *amphotericin B* as well as the standard antibiotic medications *streptomycin* and *amikacin* and refer to more details from our previous report.[13]

2.6. MTT Cell Viability Assay for anticancer characteristics

All substances directed towards the A549, HepG2, MCF-7, and NHDF cell lines were evaluated by the MTT approach. The MTT test is an enzyme-based colorimetric method that is quantitative, sensitive, and consistently supported to examine the viability, cytotoxicity, and proliferation of cells. The cells were then kept in medium consisting of varied doses of the chemical substances, which also treated with tetrazolium dye and The

cells were further incubated for four hours at 37°C. The viable cells consists of NAD(P)H-dependent oxidoreductase enzymes, which converts the water-soluble yellow MTT tetrazolium salt into water-insoluble purple-blue coloured stable formazan crystals. While the cells die, they lose the colour-changing capability. Then, the medium was separated out and blended with DMSO (100 μL). After agitating gradually twice for 5 s, the absorbance of the formazan dye generated by the cells is evaluated in an ELISA plate reader at 570 nm. The collected data (mean O.D. ± S.D) was utilized to compute the IC_{50} value compared with the standard cisplatin-anticancer medication[48] and comprehensive details of cell culture and protocols of the MTT assay are given in the previous report.[13]

2.7. Determination of Lipophilicity (hydrophobicity)

The lipophilicity of all complexes and free ligand has evaluated by the flask- shaking method through n-octanol/deionized water phase partition.[49–51] 0.015 M test compound solutions were prepared in an equal volume (1:1 $_{V/V}$) of n-octanol-deionized water (10 mL) and agitated continuously for 24 h. The organic (n-octanol) and aqueous (deionized water) phases were then cautiously segregated using a separating funnel after reaching the equilibrium condition. Finally, all compounds' concentration in each segregated phase was analysed using an electronic absorption spectrophotometer.[52] The partition coefficient (log $P_{o/w}$) and distribution coefficient (log $D_{o/w}$) findings of all substances were also acquired from molar absorption coefficients, conductivity and p^H measurements.[53, 54]

(**Note:** Refer to the manuscript for references of the experimental section: 2a (2.1 – 2.7)

3a. Results and Discussions

3.1. Structural Characterization

Table S1 Analytical and physical data of ligand (**HL**) and its mixed ligand complexes (**1–3**).

**Compounds (EF & FW)**	**Colour**	**Yield (%)**	**M.P (°C)**	**Found (Calcd) (%) C**	**H**	**N**	**M**	**$\wedge_m$**
(**HL**) ($C_{17}H_{18}N_2O_2$) (282.33) Ligand	Dark yellow	87.52	148	72.55 (72.25)	06.53 (06.37)	10.04 (09.91)	----	16.12
(**1**) ($C_{44}H_{42}N_6O_4$)Mn (773.12)[Mn(L)$_2$(bpy)]	Reddish Brown	77.55	208	68.38 (68.29)	05.48 (05.43)	10.93 (10.87)	07.23 (07.10)	45.57
(**2**) ($C_{44}H_{42}N_6O_4$)Co (777.11) [Co(L)$_2$(bpy)]	Dark Brown	80.43	216	68.08 (67.94)	05.47 (05.40)	10.78 (10.81)	07.66 (07.58)	36.73
(**3**) ($C_{44}H_{42}N_6O_4$)Ni (776.88)[Ni(L)$_2$(bpy)]	Pale brown	78.62	178	68.09 (67.96)	05.47 (05.41)	10.80 (10.81)	07.62 (07.56)	41.57

EF → Empirical Formula, FW→ Formula Weight, M.P→ Melting Point, $\wedge_m$ → Molar Conductance

3.1.1. Synthesis of Schiff base ligand (HL)

2-(4-morpholinobenzylideneamino)phenol Schiff base ligand (**HL**) was synthesized by stirring an equal molar quantity (0.01 M) of ortho amino phenol and 4-(4-morpholinyl) benzaldehyde (0.01 M) in ethanol (30 ml) and the dark yellow precipitate was obtained after refluxing for three hours and in a water bath, the solution's volume was decreased to one-third and cooled at 25 °C. In vacuum desiccators over anhydrous $CaCl_2$, the gathered pure dark yellow solid ligand was progressively dried at ambient temperature. It was discovered that the isolated ligand's (**HL**) yield was 87.52 percentages (**Scheme 1**).

3.1.2. Synthesis of mixed ligand complexes (1-3)

A solution of synthesized primary ligand (**HL**) (0.002 M) in methanol (40 mL) was gradually added to a solution of Metal(II) acetate salts (0.001 M) [$Mn^{II}(OAc)_2.4H_2O$, $Co^{II}(OAc)_2.4H_2O$, and $Ni^{II}(OAc)_2.4H_2O$] in absolute methanol and the resulting mixture solution were stirred for 30 minutes and the hot mixture of solution was slowly added into methanolic solution of 2,2'-bipyridine (bpy) as secondary ligand (0.001 M). The obtained finally mixture solutions were refluxed for 3 hours. The solid product was obtained by

filtration and they are also purified by recrystallization technique in the presence of methanol-petroleum ether mixture. Moisture and other solvents were removed by anhydrous $CaCl_2$ in the vacuum desiccators. Similar techniques were used to synthesize mixed ligand complexes (**1**–**3**), and the yield was obtained to be 77.55–80.43 % percentages (**Scheme 1**) and they were summarized in **Table S1**.

3.1.3. Analytical Methods: Elemental Analysis and Molar Conductance Studies

The synthesized ligand (**HL**) and its mixed ligand complexes (**1**–**3**) were found to be intensely coloured and they are soluble in CH_3OH, C_2H_5OH, $CHCl_3$ and DMSO. The molar conductance ($\wedge_m$) was observed in the range of 36.73–45.57 Ohm^{-1} cm^2 mol^{-1}, which was higher than that of free ligand (16.12 $Ohm^{-1}cm^2$ mol^{-1}). The molar conductance results are attributed that they are non-electrolytes. The analytical information data and physical properties of all test compounds are enclosed in **Table S1**.

3.1.4. Mass Spectra (ESI/LC-MS)

Ligand (**HL**) (**Fig. S1**) shows the molecular ion peak at *m/z* 282.3 (M) consequent to $[C_{17}H_{18}N_2O_2]^+$ and other fragmented molecular ion peaks are found at 244.3 (M+1) $[C_{14}H_{15}N_2O_2]^+$, 216.2 (M+2) $[C_{13}H_{14}N_2O]^+$, 189.3 (M+1) $[C_{11}H_{12}N_2O]^+$, 176.3 (M+2) $[C_{11}H_{12}NO]^+$, 162.2 (M+1) $[C_{10}H_{11}NO]^+$ and 118.1 (M+1) $[C_8H_7N]^+$. Complex (**1**) (**Fig. S2**) molecular ion peak at *m/z* 773.3 (M) consequent to $[C_{44}H_{42}N_6O_4Mn]^+$ and other fragmented molecular ion peaks are found at 666.2 (M) $[C_{38}H_{37}N_5O_3Mn]^+$, 447.1 (M) $[C_{24}H_{27}N_3O_2Mn]^+$, 314.3 (M+1) $[C_{15}H_{18}N_2O_2Mn]^+$ and 283.0 (M+1) $[C_{17}H_{18}N_2O_2]^+$. Complex (**2**) (**Fig. S3**) molecular ion peak at *m/z* 778.1 (M+1) corresponding to $[C_{44}H_{42}N_6O_4Co]^+$ and other fragmented molecular ion peaks are found at 671.9 (M+1) $[C_{38}H_{37}N_5O_3Co]^+$, 502.7 (M+1) $[C_{27}H_{26}N_4O_2Co]^+$, 454.2 (M+2) $[C_{25}H_{22}N_4OCo]^+$, 317.1 (M) $[C_{15}H_{18}N_2O_2Co]^+$ and 283.0 (M+1) $[C_{17}H_{18}N_2O_2]^+$. Complex (**3**) (**Fig. S4**) molecular

ion peak at *m/z* 777.6 (M+1) corresponding to $[C_{44}H_{42}N_6O_4Ni]^+$ and other fragmented molecular ion peaks are found at 671.7 (M+1) $[C_{38}H_{37}N_5O_3Ni]^+$, 502.1 (M+1) $[C_{27}H_{26}N_4O_2Ni]^+$, 453.3 (M+1) $[C_{25}H_{22}N_4ONi]^+$, 317.5 (M+1) $[C_{15}H_{18}N_2O_2Ni]^+$ and 283.0 (M+1) $[C_{17}H_{18}N_2O_2]^+$. Also, the generation of other peaks are followed by fragmented molecular ion of ligand (**HL**). The observed similar fragmented molecular ion peaks for all complexes were good agreed with molecular structures.[1]

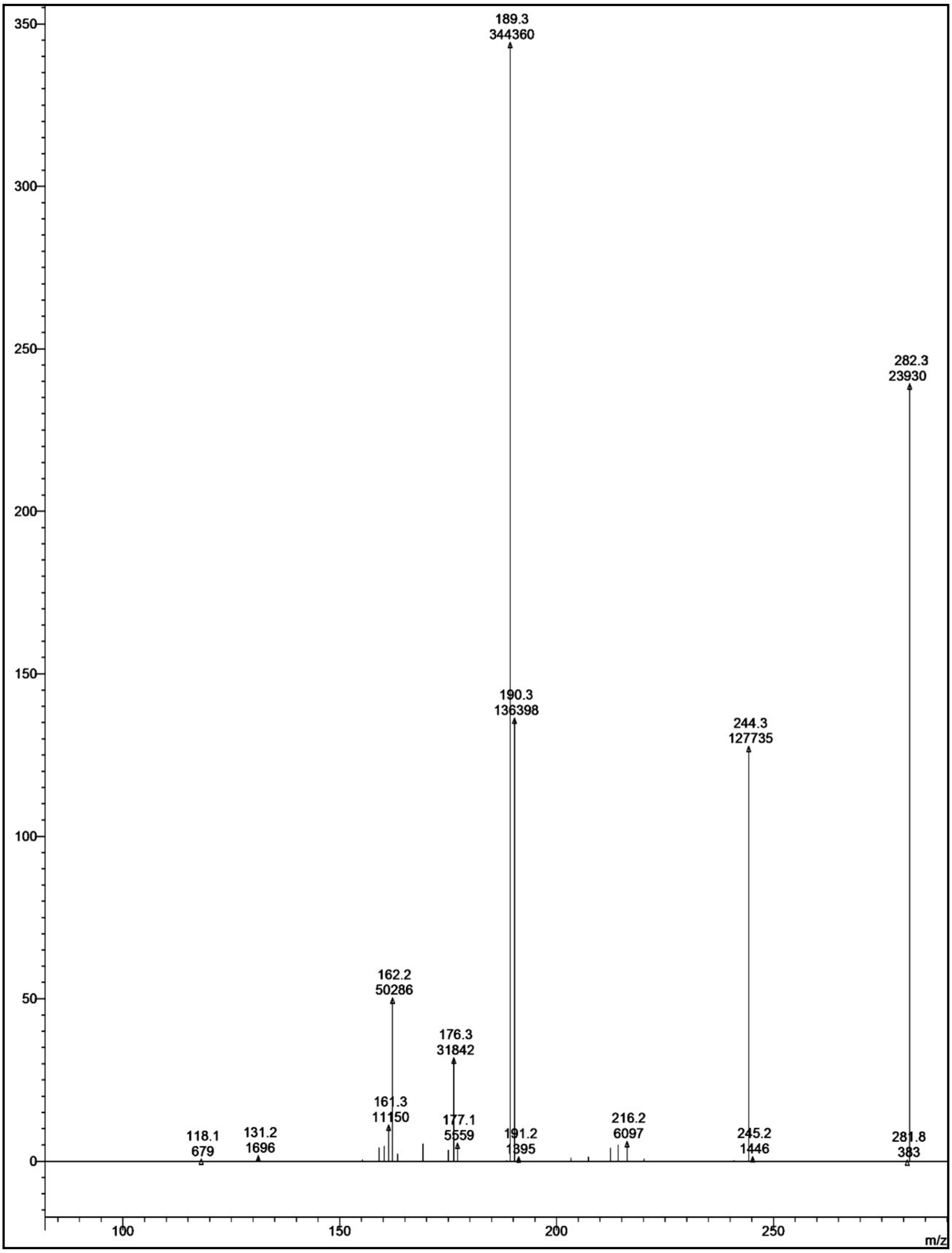

Fig. S1 ESI-LC/MS mass spectrum of ligand (**HL**).

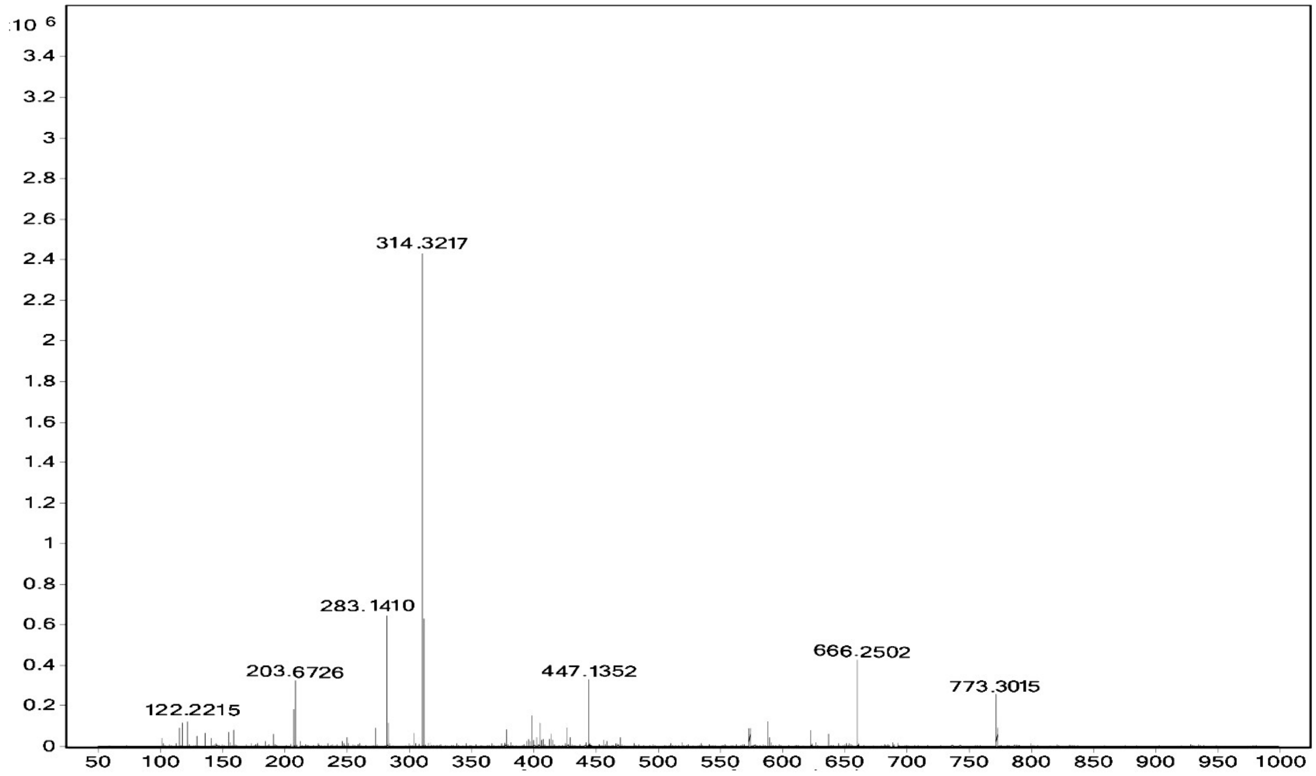

Fig. S2 ESI-LC/MS mass spectrum of complex (**1**)

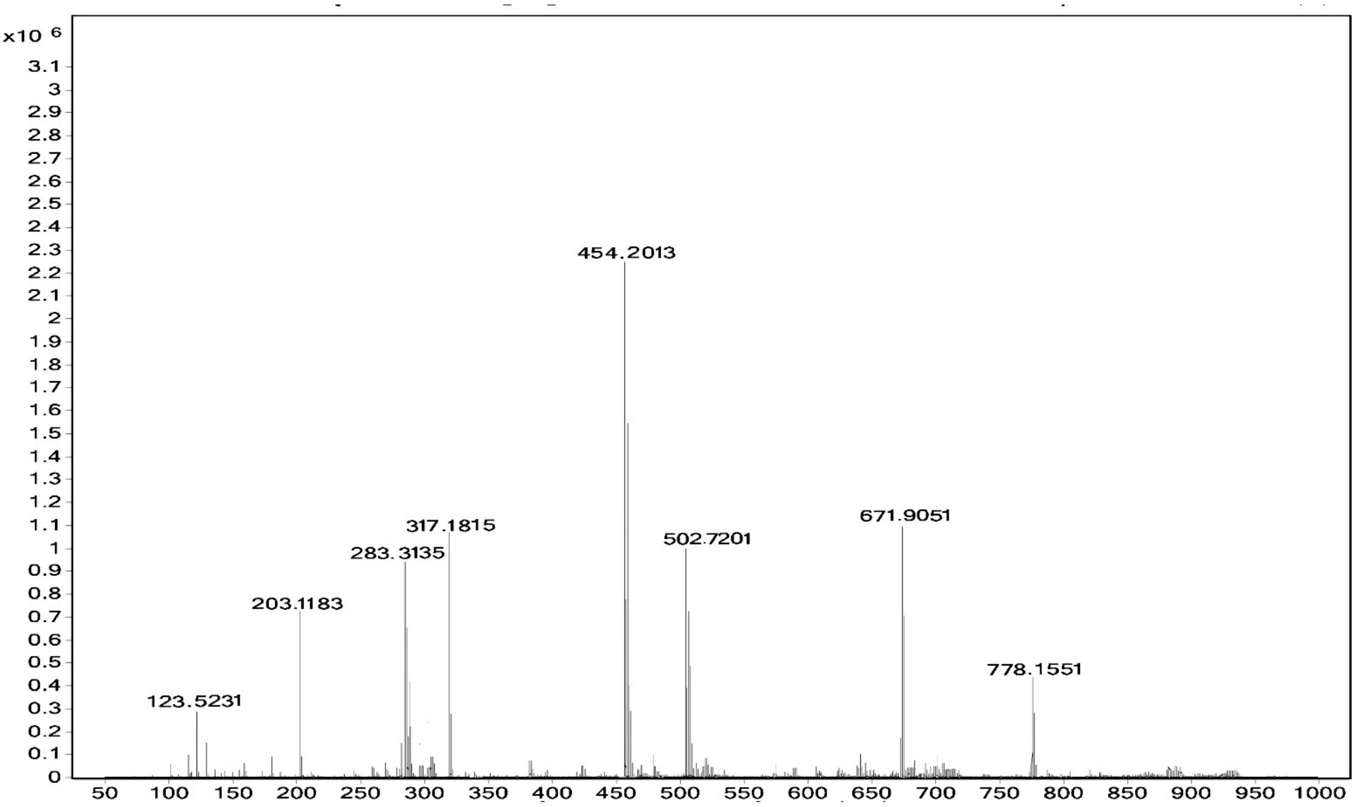

Fig. S3 ESI-LC/MS mass spectrum of complex (**2**)

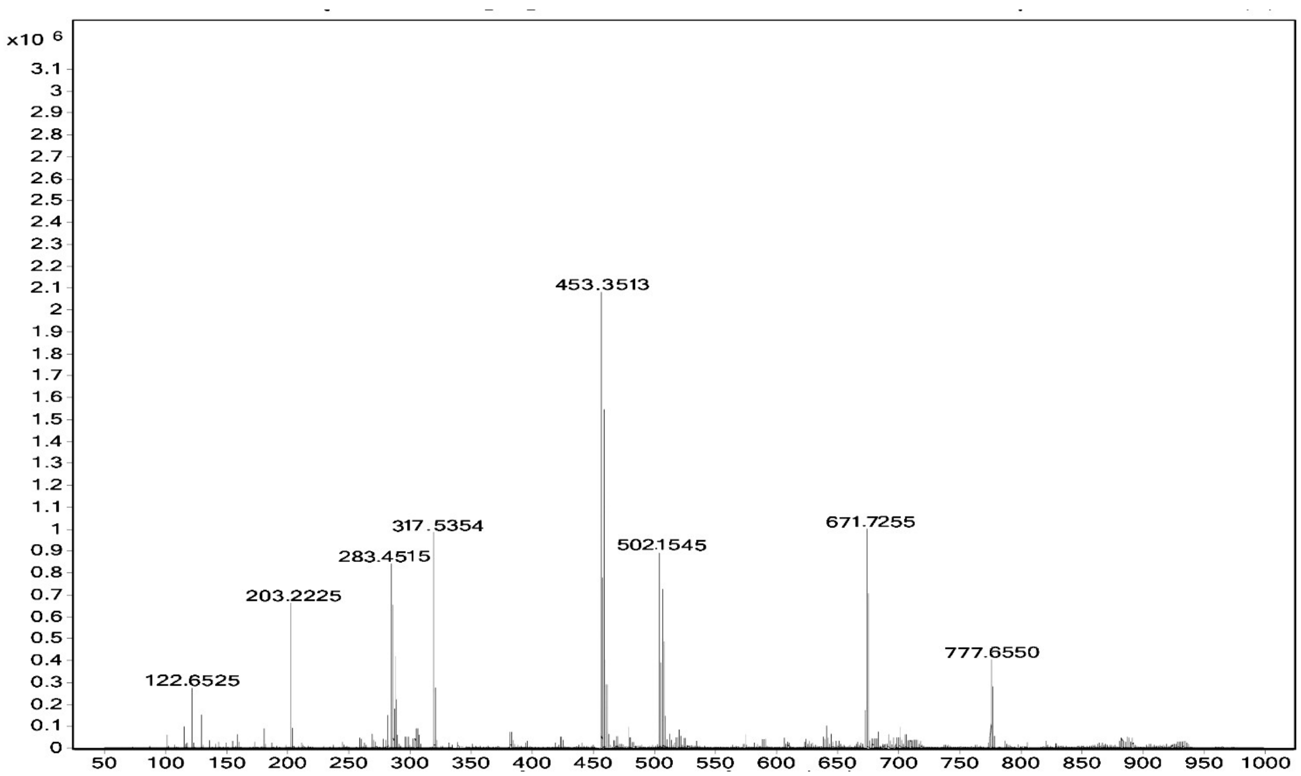

Fig. S4 ESI-LC/MS mass spectrum of complex (**3**)

3.1.5. Proton Nuclear Magnetic Resonance Spectra (^{1}H NMR)

The ^{1}H NMR spectral properties were performed in presence of $CDCl_3$ solvent using $Si(CH_3)_4$ as an internal standard. The ligand (**HL**) demonstrates the following signals in **Fig. S5.** δ values of ligand (**HL**): aromatic protons (m, 8H) at 6.45 –7.65 ppm; azomethine (-HC=N-) proton (s,1H) at 8.58 ppm; morpholinic-OCH_2 protons (t, 4H) at 3.85 ppm; morpholinic-N-CH_2 (t, 4H) at 3.30 ppm; phenolic-OH proton (s, 1H) at 5.10 ppm.[2] Furthermore, the strong singlet peak for $CDCl_3$ solvent was observed at 7.26 ppm in the ligand (**HL**). Moreover, all complexes (**1–3**) are not supported to ^{1}H NMR spectral properties, which consist of $3d^5$ (**1-Mn^{II}**), $3d^7$ (**2-Co^{II}**), and $3d^8$ (**3-Ni^{II}**) electronic configurations with paramagnetic due to weak filed and high spin ligand and since nickel complex (**3**) consisting of $3d^8$, they have two unpaired electrons in the e_g orbital on accordance with crystal field theory, they also not supported to ^{1}H NMR spectral

properties due to low splitting degeneration with low energy gap in the presence of weak filed and high spin ligand.

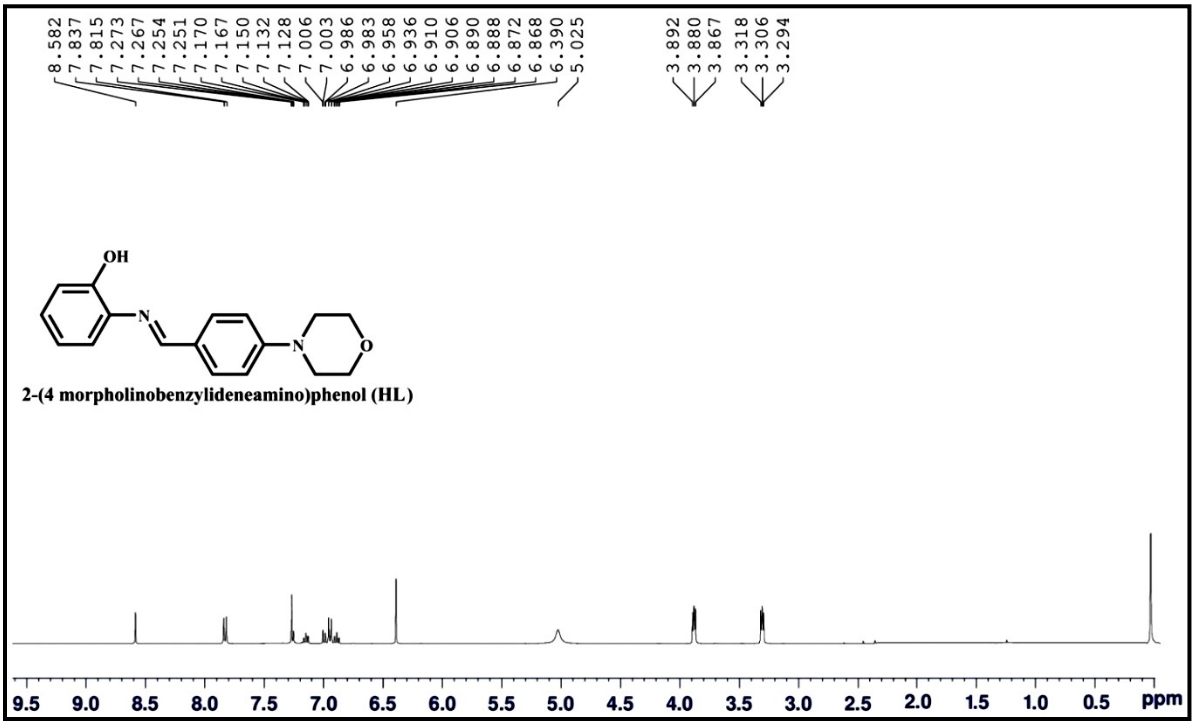

Fig. S5 ^{1}H NMR spectra of ligand (**HL**).

3.1.6. ^{13}C NMR spectrum

The ^{13}C NMR spectrum of the ligand (**HL**) was recorded by a Bruker Advance III HD Nanobay 400 MHz spectrometer operating in Fourier transform mode in the presence of $CDCl_3$ at room temperature.[3, 4] The following signals of ligand (**HL**) were demonstrated in **Fig. S6**. The observed peak at 156.84 ppm was assigned iminic (azomethine) carbons. The aromatic carbons of the ligand (**HL**) are observed in the range 113.47 – 135.88 ppm. The observed signal in the region 151.95–153.61 ppm indicates the carbon atom adjacent to the phenolic oxygen in the free ligand (**HL**). The peak of morpholinic-$\underline{C}H_2$-O-$\underline{C}H_2$ was found at 66.64 ppm. The chemical shifts for =N-CH_2-$\underline{C}H_2$-N and =N-$\underline{C}H_2$-CH_2-N groups were also obtained at 65.19 and 63.35 respectively. The observed peaks at 47.87 and 47.29 ppm are attributed to morpholinic-$\underline{C}H_2$-N-$\underline{C}H_2$ carbons. Also, the strong triplet peaks for $CDCl_3$ solvent were observed in the range of 76.74-77.38 ppm. Moreover, all complexes (**1–3**) are

not supported to ^{13}C NMR spectral properties due to weak filed and high spin ligand with paramagneticsm.

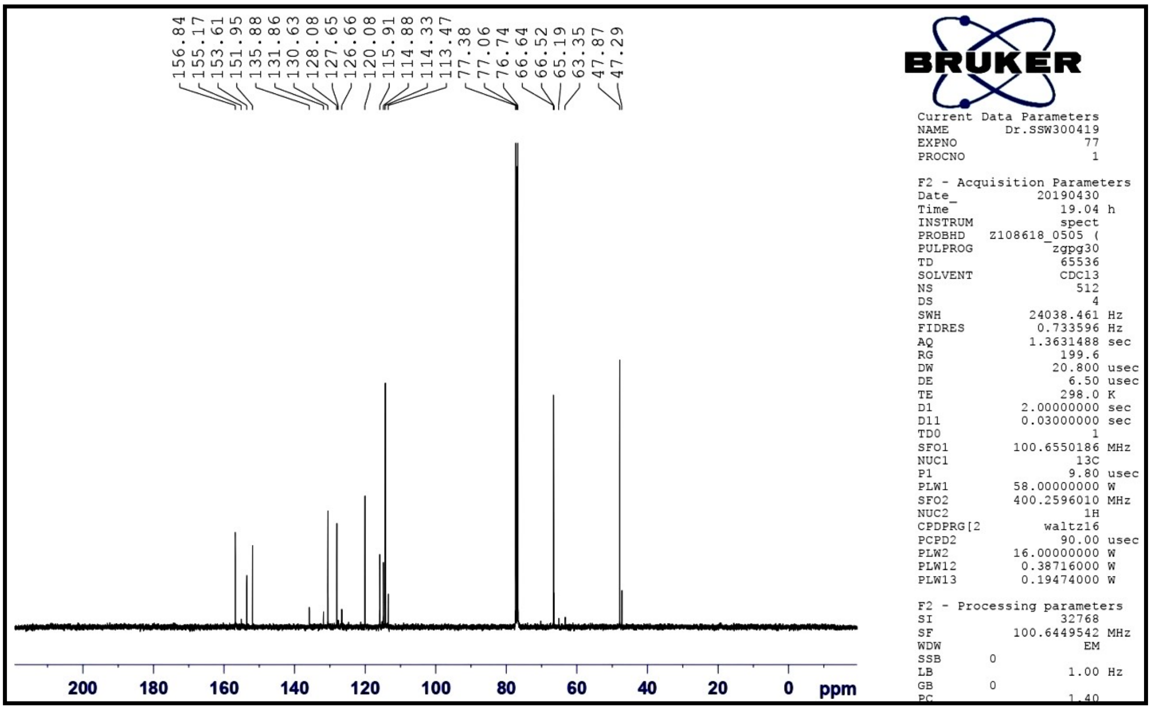

Fig. S6 ^{13}C NMR spectrum of ligand (**HL**).

3.1.7. Fourier Transform Infrared Spectra (FT-IR)

The IR spectra were executed using Shimadzu FT-IR spectrometer (4000–400 cm^{-1}) using KBr pellets. The FTIR spectra of the mixed ligand complexes (**1**–**3**) investigated the frequency changes of free ligand (**HL**) due to the complexation and their observed results were also summarized in **Table S2.** FT-IR spectrum of ligand (**HL**) exhibits a strong sharp band of the azomethine group (-HC=N-) at 1632 cm^{-1} the band is also shifted to lower frequencies in the range of 1602–1610 cm^{-1} for all complexes(**1**–**3**) due to strong complexation with the central metal ion.[5] The sharp peak of the hydroxy group was identified at 3403 cm^{-1} in the free ligand (**HL**) and the same peak disappeared in the spectra of all complexes due to deprotonation of -OH group during complexation.[6] Also, the broad band was observed in the region of 3412–3422 cm^{-1} due to the formation of hydrogen band between iminic hydrogen and a hydroxyl group. It is also evidenced that the observed phenolic C-O group at 1268 cm^{-1} in the ligand (**HL**) is shifted to higher

frequencies in the range of 1288–1316 cm^{-1} in all complexes (**1**–**3**) pointing out confirming deprotonation of the phenolic–OH on chelation.[7] In the FT-IR spectra of all mixed ligand complexes, the peaks consequent to the ring stretching frequencies of γ(C=N) and γ(C=C) at 1503, 1422 cm^{-1} of free 2,2'-bipyridine were shifted to the region of 1516–1542 cm^{-1} and 1416–1418 cm^{-1} respectively, which indicate the complexation owing to coordination of the heterocyclic nitrogen atoms to the metal ion.[8] In the far IR spectra of all complexes, the medium bands were found in the region 450–478 cm^{-1} and 518–528 cm^{-1}, which are consequent to M-N and M-O vibrations respectively and other absorption bands like morpholinic-C-N-C, morpholinic-C-O-C, aromatic C-H and aliphatic C-H have no appreciable changes in the complexes (**1**–**3**) (**Fig. S7**).

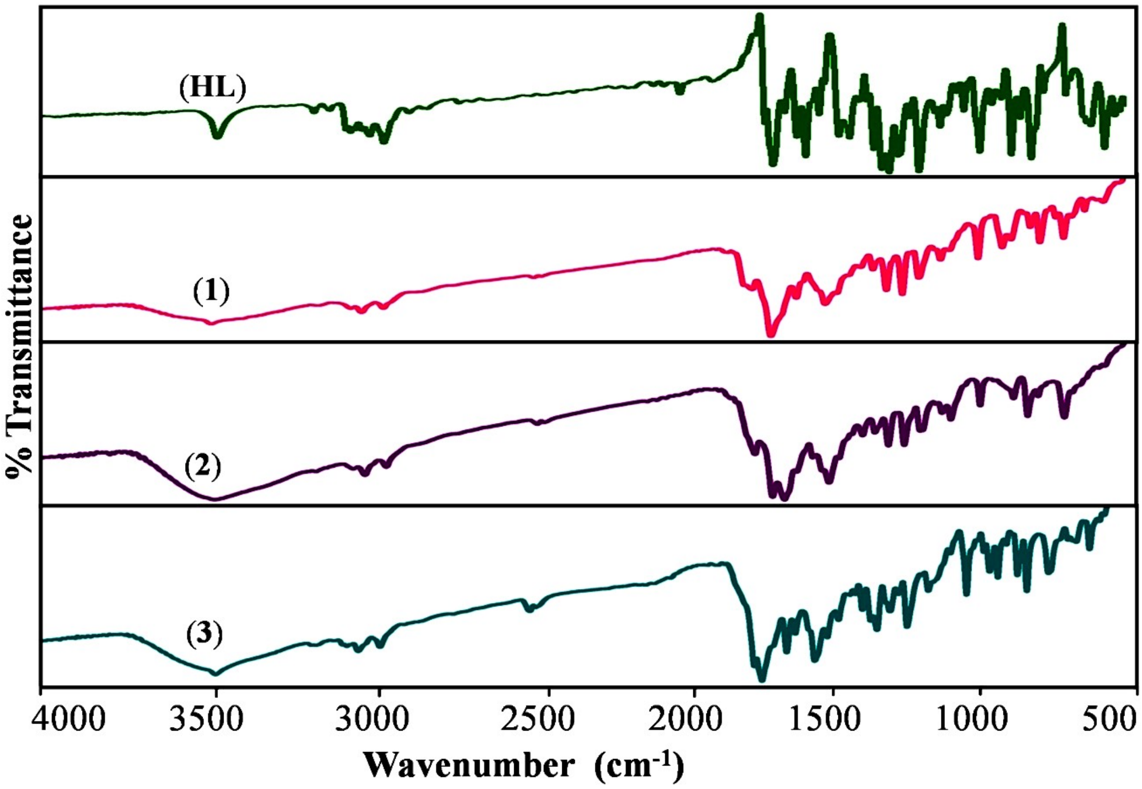

Fig. S7 FT-IR spectra of ligand (**HL**) and its mixed ligand complexes (**1**–**3**).

Table S2 FT-IR spectral data (cm^{-1}) of the ligand (**HL**) and its complexes (**1**–**3**).

Com pounds	HC=N	Ph-C-O	Morp-C-N-C	Morp-C-O-C	C-H			Ph-OH / H-bond	Bpy C=N & C=C	M-N	M-O
					Ar-C-H	Ali-C-H	Iminic H-C=N				
(**HL**)	1632	1268	1341	1112 (s) 1181(as)	2974	2918	2858	3403	---	---	---
(**1**)	1608	1304	1352	1115 (s) 1166(as)	2964	2924	2850	3416	1516 1417	467	528
(**2**)	1610	1312	1345	1114 (s) 1173(as)	2964	2929	2850	3422	1542 1418	457	526
(**3**)	1602	1307	1349	1112 (s) 1166(as)	2972	2926	2851	3414	1524 1418	478	526

s → symmetry, as → asymmetry, Phen-C-O → Phenolic C-O, Morp-C-N-C → Morpholinic C-N-C, Morp-C-O-C → Morpholinic C-O-C, Ar-C-H → Aromatic C-H, Ali-C-H → Aliphatic C-H Ph-OH → Phenolic OH, bpy→ 2,2'-bipyridine.

3.1.8. Electronic Absorption Spectra and Magnetic Susceptibility

Ligand (**HL**) and its complexes (**1**–**3**) were carried out in methanol by an electronic absorption spectrophotometer. The observed results of absorption maxima and magnetic moment are summarized in **Table S3**. The free ligand (**HL**) exhibited three absorption bands at 358 nm (27,933 cm^{-1}), 237 nm (42,194 cm^{-1}) and 205 nm (48,780 cm^{-1}), which assigns n→π*, π→π* and π→π* transitions respectively owing to the azomethine chromophore and phenyl rings.[9] The bands are transferred into a higher wavelength, which attributes the formation of complex due to the lone pair electron of a sp^2-hybridized orbital of the imino nitrogen in the ligand donates to the metal centre. Moreover, Cobalt complex (**2**) demonstrated the bands at 648 nm (15,432 cm^{-1}) and 498 nm (20,080 cm^{-1}) consequent to $^4T_{1g}$ (F) → $^4A_{2g}$ (F) (υ_2) and $^4T_{1g}$ (F) → $^4T_{1g}$ (P) (υ_3) transitions. On the other hand, the lowest energy bands consequent to $^4T_{1g}$ (F) → $^4T_{2g}$ (F) (υ_1) transition are not found in the IR region (above 1000 nm) and their μ_{eff} value is observed at 5.20 BM, which was also greater than the spin-only value due to orbital angular momentum contribution in d^7 system and the results propose to an octahedral structure.[10–12] Nickel complexes (**3**) displayed three bands at 918 nm (10,893 cm^{-1}), 562 nm (17,793 cm^{-1}) and 420 nm (23,810 cm^{-1}) corresponding to $^3A_{2g}$ (F) → $^3T_{2g}$ (F) (υ_1), $^3A_{2g}$ (F) → $^3T_{1g}$ (F) (υ_2) and $^3A_{2g}$ (F) →

$^3T_{1g}$ (P) (υ_3) transitions and their μ_{eff} value was found in the range of 3.18 B.M. The results lead to an octahedral geometry [13–15] (**Table S3 & Fig. S8**). Manganese complex (**1**) has no appearance of absorption bands in the visible region and CFT does not envisage the d-d transitions due to d^5 electronic configuration and have very weakly coloured due to spin forbidden d-d transition and Laporte forbidden.[16] The obtained results suggest that the complex (**1**) belongs to an octahedral environment around the central metal(II) ion.

Table S3 Electronic spectral data and magnetic susceptibility values of the synthesized ligand (**HL**) and its complexes (**2–3**).

Compounds	Band Position λ_{max} nm (γ-cm^{-1})	Assignment	μ_{eff} (B.M)	Geometry
(**HL**)	358 (27,933)	$n\rightarrow\pi^*$	--	--
	237 (42,194)	$\pi\rightarrow\pi^*$		
	205 (48,780)	$\pi\rightarrow\pi^*$		
(**2**)	648 (15,432)	$^4T_{1g}$ (F) → $^4A_{2g}$ (F) (υ_2)	5.20	Octahedral
	498 (20,080)	$^4T_{1g}$ (F) → $^4T_{1g}$ (P) (υ_3)		
(**3**)	918 (10,893)	$^3A_{2g}$ (F) → $^3T_{2g}$ (F) (υ_1)	3.18	Octahedral
	562 (17,793)	$^3A_{2g}$ (F) → $^3T_{1g}$ (F) (υ_2)		
	420 (23,810)	$^3A_{2g}$ (F) → $^3T_{1g}$ (P) (υ_3)		

INCT→ Intra-ligand charge transfer, μ_{eff} → Effective magnetic moment, B.M→ Bohr magnetons. $Wavenumber\ (\gamma\ cm^{-1}) = \left(\frac{1}{wavelength(nm)}\right) = \lambda^{-1} \times 10^7$ cm^{-1}.

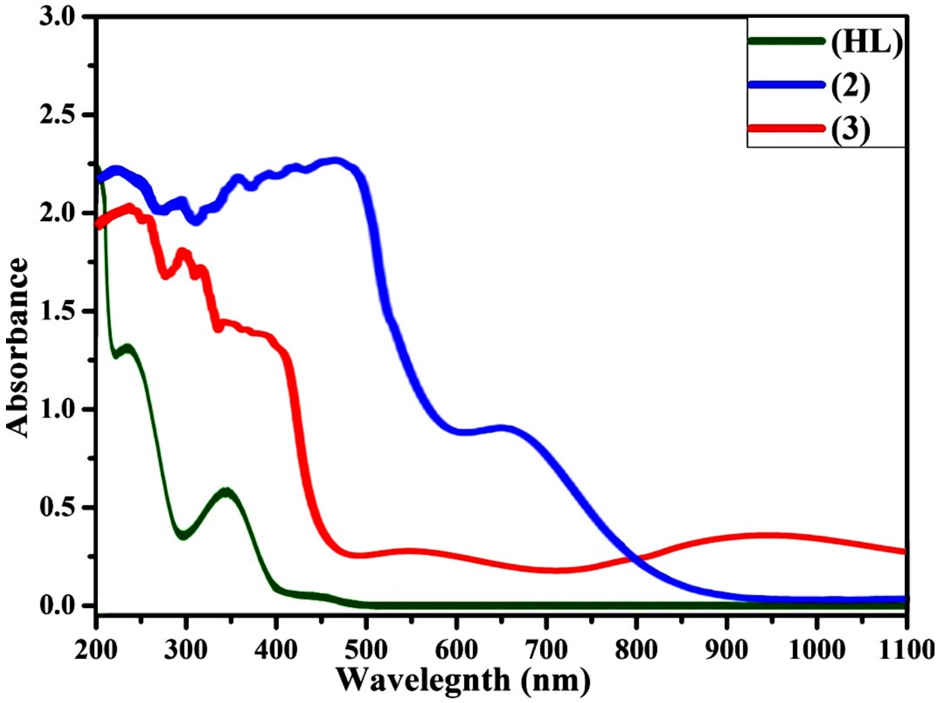

Fig. S8 Electronic spectra of ligand (**HL**) and its complexes (**2–3**).

3.1.9. Thermogravimetric Analysis (TGA)

TGA thermograms of the mixed ligand complexes (**1**–**3**) have been recorded in the temperature range from 40 °C to 730 °C (**Figs. S9 & S10**). The stages of decomposition, temperature range, decomposition products, the obtained mass loss and calculated mass loss percentages of complexes (**1**–**3**) are summarized in **Table S4.**[17,18] There are exposed three steps during the thermal decomposition of complexes. In the first step process at 40–180°C, the observed weight losses of decomposition of [M^{II} $(L)_2$ (bpy)] complexes were found in the range of 10.98–11.06 % respectively, which corresponds to the loss of morpholine moieties (C_4H_8NO). In the second degradation stages, the weight losses were evaluated in the temperature range of 180–420 °C, which is further confirmed that the elimination of the rest aromatic part of ligand ($C_{13}H_9NO$) present in the complexes and they are found in the range of 25.02–25.33 % for complexes (**1**–**3**). Similarly, the obtained weight losses in the third degradation stage at 420–730 °C were pointed out in the range of 55.18–56.42 % elimination of ligand $C_{17}H_{17}N_2O$ (L) and co-ligands 2,2'-bipyridine [$C_{10}H_8N_2$] in the mixed ligand complexes (**1**–**3**). The observed final percentage of rest products of metal oxide (MO) residue at above 730 °C was found in the range of 9.24–9.68 % for mixed ligand complexes (**1**–**3**). The thermal degradation steps of complexes (**1**–**3**) are shown in the flow chart (**Fig. S9**). Based on the above observations, the proposed structures of all complexes have been demonstrated in the experimental section.

Table S4 Thermal analysis of complexes (**1**–**3**) by TGA method.

Complexes (M.W)	Step	Temperature range (°C)	% Weight loss [found (calcd)]	Assignment
(**1**) [Mn(L)$_2$(bpy)] ($C_{44}H_{42}N_6O_4$)Mn (773.12)	I	40–180	11.05 (11.12)	C_4H_8NO
	II	180–420	25.33 (25.22)	$C_{13}H_9NO$
	III	420–730	56.42 (56.54)	$C_{17}H_{17}N_2O$,$C_{10}H_8N_2$
	Residue	>730	09.24 (09.17)	MnO
(**2**) [Co(L)$_2$(bpy)] ($C_{44}H_{42}N_6O_4$)Co (777.11)	I	40–180	10.98 (11.06)	C_4H_8NO
	II	180–420	25.14 (25.09)	$C_{13}H_9NO$
	III	420–730	56.18 (56.25)	$C_{17}H_{17}N_2O$, $C_{10}H_8N_2$
	Residue	>730	09.68 (09.64)	CoO
(**3**) [Ni(L)$_2$(bpy)] ($C_{44}H_{42}N_6O_4$)Ni (776.88)	I	40–180	11.00 (11.06)	C_4H_8NO
	II	180–420	25.02 (25.10)	$C_{13}H_9NO$
	III	420–730	56.32 (56.27)	$C_{17}H_{17}N_2O$, $C_{10}H_8N_2$
	Residue	>730	09.45 (09.61)	NiO

M.W → Molecular Weight, TGA →Thermo Gravimetric Analysis.

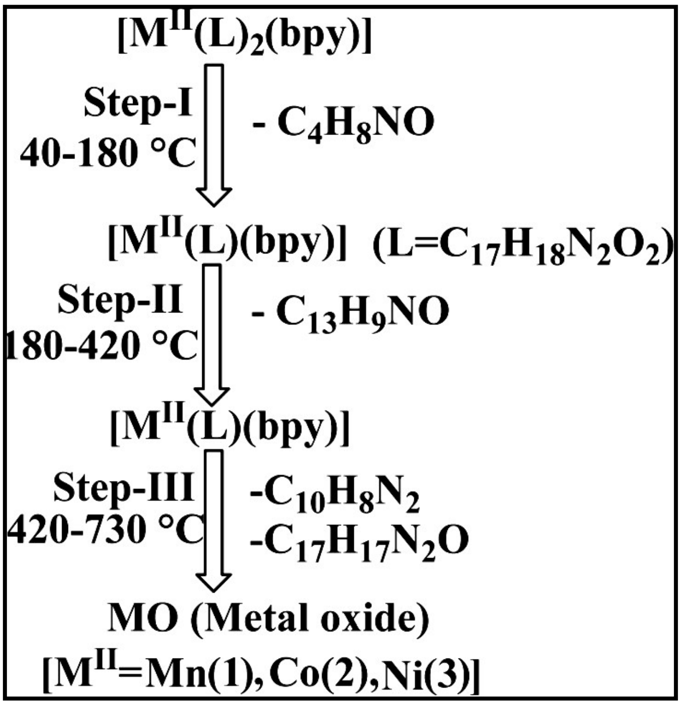

Fig. S9 The various stages for thermal decomposition of complexes (**1**–**3**) at temperature range of 40–730 °C.

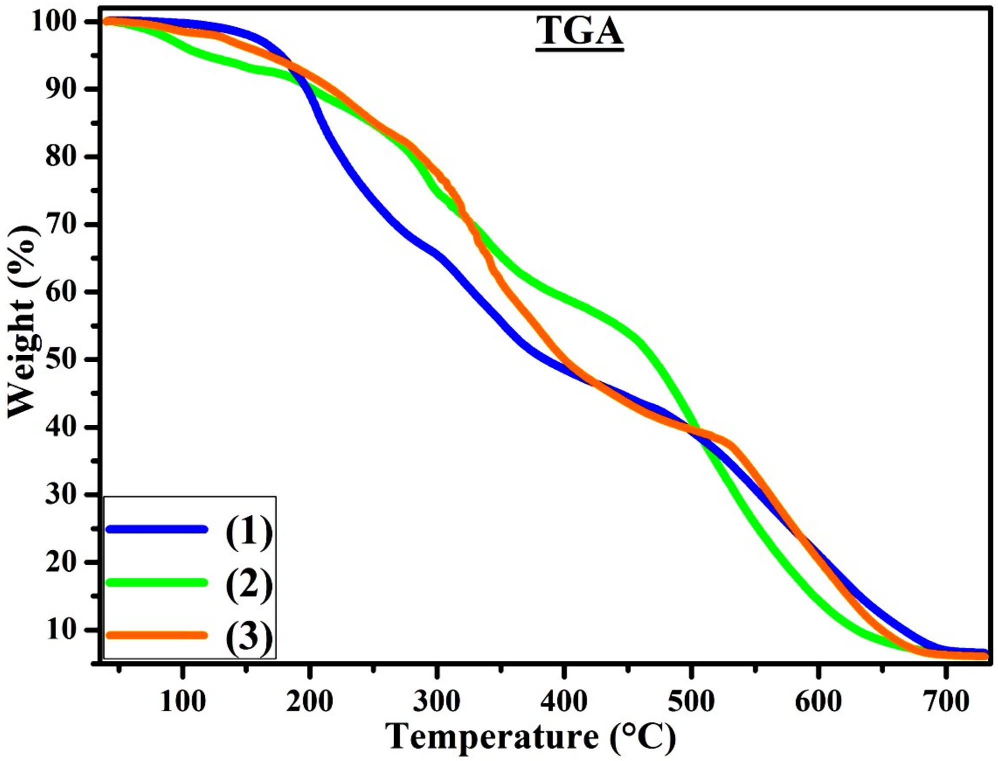

Fig. S10 TG plots of complexes (**1**–**3**) recorded under nitrogen atmosphere between the temperature range 40 and 750 °C at a heating rate of 20° C/min.

3.1.11. Single-Crystal X-ray Diffraction Analysis (SCXRD)

Schiff base ligand (**HL**) was obtained as a light yellow coloured single crystalline form in the presence of ethyl acetate medium by slow evaporation of the chloroform and ethanol mixture solution. Single-crystal XRD analysis of Schiff base ligand (**HL**) was performed to attain detailed information on molecular conformations in the solid-state. This analysis also proves the molecular structure and atom connectivity as shown in **Figs. 11** & **S12**. The information on the XRD data collection and structure refinements are enclosed in **Table S5** and hydrogen bonding geometry is given in **Table S6**. The crystallographic data were gathered at room temperature with the assistance of MoKα radiation in the wavelength of 0.71073 Å by Bruker AXS KAPPA APEX-2 diffractometer equipped with a graphite monochromator. The molecular structure of ligand was resolved by direct methods and refined by full-matrix least-squares calculations using the SHELXL-2014 program.[19] The

ligand (**HL**) crystalline form was isomorphous in the monoclinic system and its space group P21/c with molecular formula $C_{17}H_{18}N_2O_2$ in the unit cell. The obtained results were also good agreed with spectral and analytical data. The bond length (Å) between carbon-carbon in the ligand (**HL**) was obtained as C(1)-C(2)→1.197(11), C(2)-C(3)→1.423(13), C(3)-C(4)→1.466(14), C(4)-C(5)→1.410(5), C(5)-C(6)→1.446(5), C(7)-C(8)→1.229(11), C(8)-C(9)→1.382(12), C(9)-C(10)→1.193(11), C(10)-C(11)→1.562(12), C(11)-C(12)→1.377(11), C(12)-C(13)→1.223(12), C(14)-C(15)→1.405(13) and C(16)-C(17)→1.348(13). The bond length (Å) between carbon-hydrogen was observed as C(2)-H(2), C(2)-H(2), C(3)-H(3), C(4)-H(4), C(5)-H(5), C(7)-H(7), C(9)-H(9), C(10)-H(10), C(12)-H(12), C(13)-H(13)→0.9300, C(14)-H (14A), C(14)-H (14B), C(15)-H (15A), C(15)-H (15B), C(16)-H (16A), C(16)-H (16B), C(17)-H (17A), C(17)-H (17B)→0.9700. Also, the bond lengths (Å) for carbon-nitrogen, carbon-oxygen and oxygen-hydrogen atoms were observed as C(6)-N(1)→1.215(10), C(7)-N(1)→1.360(11), C(11) N(2)→1.266(10), C(14)-N(2)→1.467(12), C(17)-N(2)→1.587(11), C(1)-O(1)→1.420(10), C(15)-O(2)→1.491(12), C(16)-O(2)→1.463(13) and O(1)-H(1O)→1.28(14). The bond angle (°) for carbon-carbon-carbon in the ligand (**HL**) was found as C(1)-C(2)-C(3)→105.2(8), C(2)-C(3)-C(4)→137.5(8), C(5)-C(4)-C(3)→120.9(8), C(4)-C(5)-C(6)→96.7(7), C(5)-C(6)-C(1)→139.6(7), C(7)-C(8)-C(9)→109.4(8), C(7)-C(8)-C(13)→122.4(9), C(9)-C(8)-C(13)→128.1(8), C(10)-C(9)-C(8)→107.1(9), C(9)-C(10)-C(11)→124.8(9), C(12)-C(11)-C(10)→128.3(8), C(13)-C(12)-C(11)→105.4(9), C(12)-C(13)-C(8)→126.3(9). Also, The bond angle (°) for carbon-carbon-oxygen, oxygen-carbon-hydrogen, nitrogen-carbon-carbon and carbon-nitrogen-carbon in the ligand (HL) was found as C(2)-C(1)-O(1)→105.8(8), O(1)-C(1)-C(6)→134.4(7), C(14)-C(15)-O(2)→116.6(8), O(2)-C(15)-H(15A)→108.1,O(2)-C(15)-H(15B)→108.1, C(17)-C(16)-O(2)→96.1(9), O(2)-C(16)-H(16A)→112.5, O(2)-C(16)-H(16B)→112.5, C(1)-O(1)-H(1O)→116(5), C(16)-O(2)-C(15)→121.0(7), N(1)-C(6)-C(5)→106.2(7), N(1)-C(6)-

C(1)→113.9(7), C(8)-C(7)-N(1)→127.1(9), C(6)-N(1)-C(7)→119.3(7), C(11)-N(2)-C(14)→99.3(7), C(11)-N(2)-C(17)→119.8(7), C(14)-N(2)-C(17)→127.9(7). The two phenyl rings are bridged by the iminic group (HC=N) in the molecular structure of ligand and its bond length (Å) of HC=N was found at 1.360(11) and its bond angle of C(6)=N(1)-C(7) around 119.3(7)°. Also, the bond angle of nitrogen in the sp^3 hybridized morpholine ring system [C(14)-N(2)-C(17)] was 127.9(7)°. The dihedral angle between these two phenyl rings was observed at 7.8(13)°, which demonstrates that both the phenyl rings are almost coplanar. However, the atoms O(1) slightly deviates from the least-quares plane of the phenyl ring.[20] Furthermore, the phenyl rings are stacked face-to-face in crystalline lattice leading to π–π interactions, which are further supported to assemble the molecules in the unit cell. The intermolecular interactions, especially classical and non-classical H-bonds are playing an essential role in the generation of the crystal and their physicochemical characteristics.[21] These weak intermolecular interactions and hydrogen bonding interactions can be classified and analyzed with graph-set nomenclature, which is more helpful in comparing the stability of the interactions.[22] However, the crystal packing is stabilized through intra-molecular hydrogen bonding interactions (O-H--N) and π–π interactions. The H-bonding dimensions are summarized in **Table S6** and The crystal structure and the unit cell packing arrangement of molecules of the ligand (**HL**) is represented in **Figs. S11 and S12**. Moreover, the morpholine fragments are stabilized in the crystal packing by forming intermolecular hydrogen bonds C(2)-H(2)...O(2) between the morpholine ring oxygen O(2) and the hydrogen atom of phenyl ring [C(2)-H(2)][23] and the packing may also be completed by hydrophobic van der Waals force attractions between the morpholine bearing ligands. In addition, the intramolecular hydrogen bonds O(1)-H...N between the hydroxyl group and iminic group, may lead to the further stabilization of the molecular structure of the ligand (**HL**) (**Table S6**). In addition, some other crystal data including structure refinement, atomic coordinates, bond lengths [Å] and

angles [°], equivalent isotropic displacement and anisotropic displacement parameters for the ligand (**HL**) are also summarized in the section (**Tables S7 – S11**).

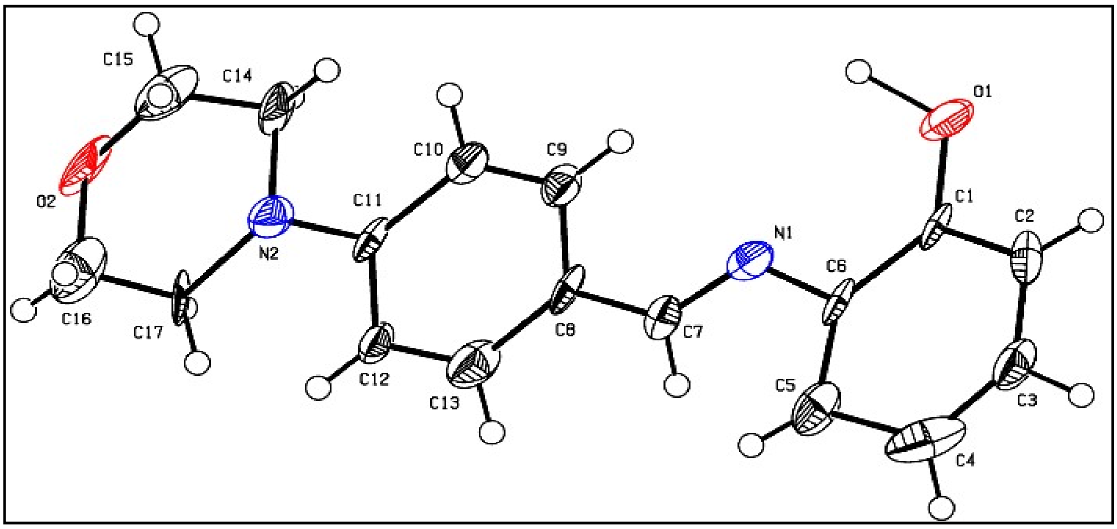

Fig. S11. The crystal structure of the ligand (**HL**) in ORTEP view.

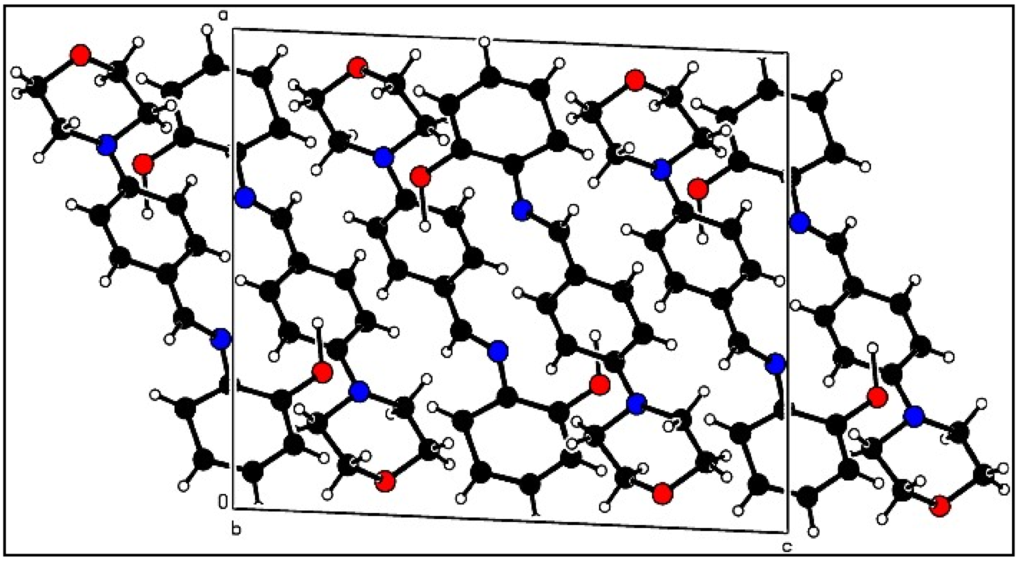

Fig.S12. Unit cell packing diagram of the crystal ligand (**HL**).

Table S5 Crystal data and structure refinement for the ligand (**HL**).

Empirical formula	$C_{17} H_{18} N_2 O_2$
Formula weight	282.33
Temperature	101 (2) K
Wavelength	0.71073 Å
Crystal system	Monoclinic
Space group	$P2_1/c$
Unit cell dimensions	a = 14.86 78 (12) Å, α = 90°<br>b = 7.5710 (6) Å, β = 93.197 (2)°<br>c = 12.3380 (8) Å, γ = 90°
Volume	1386.65 (18) $Å^3$
Z, calculated Density	4, 1.352 Mg/m^3
Absorption coefficient	0.090 mm^{-1}
F(000)	600
Crystal size	0.312 × 0.311 × 0.078 mm
Theta range for data collection	θ = 2.744 to 25.332°.
Limiting indices (Index ranges)	h = –17 → 17<br>k = –09 → 09<br>l = –14 → 14
Reflections collected	37050
Independent reflections	2505 [R(int) = 0.5296]
Completeness to Δ = 25.242°	98.8 %
Refinement method	Full-matrix least-squares on F2
Data / restraints / parameters	2505 / 0 / 218
Goodness-of-fit on F2	1.222
Final R indices [I > 2 σ (I)]	R1 = 0.1442, wR2 = 0.1293
R indices (all data)	R1 = 0.1002, wR2 = 0.0694
Largest diff. peak and hole	0.987 and –0.546 e.$Å^{-3}$

Table S6 Hydrogen bonds for the ligand (**HL**) (Å & °).

**D-H...A**	**d(D-H)**	**d(H...A)**	**d(D...A)**	**<(DHA)**
C(2)-H(2)...O(2)#1	0.93	2.53	3.255(12)	135.2

Symmetry transformations used to generate equivalent atoms: #1 x-1,-y+3/2,z+1/2

Table S7 Atomic coordinates (×10^4) and equivalent isotropic displacement parameters (Å^2 × 10^3) for Schiff base ligand (**HL**). U(eq) is defined as one third of the trace of the orthogonalized U^{ij} tensor for the ligand (**HL**).

Atoms	x	y	z	U(eq)
C(1)	2302(6)	7693(10)	5879(6)	26(2)
C(2)	1389(7)	7688(11)	6100(6)	35(2)
C(3)	781(7)	7005(11)	5358(7)	41(3)
C(4)	989(7)	6332(12)	4470(7)	58(3)
C(5)	2036(6)	6333(11)	4147(5)	43(3)
C(6)	2575(6)	6999(10)	4947(5)	26(2)
C(7)	3919(7)	6180(11)	4117(6)	30(2)
C(8)	4821(6)	6283(11)	3817(6)	31(2)
C(9)	5443(7)	7397(10)	4336(6)	31(2)
C(10)	6282(7)	7544(11)	3989(6)	34(2)
C(11)	6589(6)	6600(10)	3118(6)	27(2)
C(12)	5979(7)	5463(11)	2607(6)	32(2)
C(13)	5122(8)	5356(12)	2971(7)	44(3)
C(14)	8153(7)	7373(13)	3479(6)	41(2)
C(15)	8989(8)	7814(13)	2922(8)	56(3)
C(16)	8596(8)	6145(14)	1467(9)	61(3)
C(17)	7742(7)	5617(12)	1916(6)	35(2)
N(1)	3516(6)	7145(9)	4780(5)	34(2)
N(2)	7452(6)	6836(9)	2719(5)	37(2)
O(1)	2918(5)	8375(9)	6614(4)	51(2)
O(2)	9292(5)	6503(10)	2254(5)	64(2)

Table S8 Bond lengths [Å] and angles [°] for the ligand (**HL**).

Positions of atoms	Bond lengths [Å] and bond angles [°]
C(1)-C(2)	1.197(11)
C(1)-O(1)	1.420(10)
C(1)-C(6)	1.550(12)
C(2)-C(3)	1.423(13)
C(2)-H(2)	0.93
C(3)-C(4)	1.466(14)
C(3)-H(3)	0.93
C(4)-C(5)	1.410(5)
C(4)-H(4)	0.93
C(5)-C(6)	1.446(5)
C(5)-H(5)	0.93
C(6)-N(1)	1.215(10)
C(7)-C(8)	1.229(11)
C(7)-N(1)	1.360(11)
C(7)-H(7)	0.93
C(8)-C(9)	1.382(12)
C(8)-C(13)	1.521(13)
C(9)-C(10)	1.193(11)
C(9)-H(9)	0.93
C(10)-C(11)	1.562(12)
C(10)-H(10)	0.93
C(11)-N(2)	1.266(10)
C(11)-C(12)	1.377(11)
C(12)-C(13)	1.223(12)
C(12)-H(12)	0.93
C(13)-H(13)	0.93
C(14)-C(15)	1.405(13)
C(14)-N(2)	1.467(12)
C(14)-H(14A)	0.97
C(14)-H(14B)	0.97
C(15)-O(2)	1.491(12)
C(15)-H(15A)	0.97
C(15)-H(15B)	0.97
C(16)-C(17)	1.348(13)
C(16)-O(2)	1.463(13)
C(16)-H(16A)	0.97
C(16)-H(16B)	0.97
C(17)-N(2)	1.587(11)
C(17)-H(17A)	0.97
C(17)-H(17B)	0.97
O(1)-H(1O)	1.28(14)
C(2)-C(1)-O(1)	105.8(8)

C(2)-C(1)-C(6)	119.8(8)
O(1)-C(1)-C(6)	134.4(7)
C(1)-C(2)-C(3)	105.2(8)
C(1)-C(2)-H(2)	127.4
C(3)-C(2)-H(2)	127.4
C(2)-C(3)-C(4)	137.5(8)
C(2)-C(3)-H(3)	111.3
C(4)-C(3)-H(3)	111.3
C(5)-C(4)-C(3)	120.9(8)
C(5)-C(4)-H(4)	119.5
C(3)-C(4)-H(4)	119.5
C(4)-C(5)-C(6)	96.7(7)
C(4)-C(5)-H(5)	131.6
C(6)-C(5)-H(5)	131.6
N(1)-C(6)-C(5)	106.2(7)
N(1)-C(6)-C(1)	113.9(7)
C(5)-C(6)-C(1)	139.6(7)
C(8)-C(7)-N(1)	127.1(9)
C(8)-C(7)-H(7)	116.5
N(1)-C(7)-H(7)	116.5
C(7)-C(8)-C(9)	109.4(8)
C(7)-C(8)-C(13)	122.4(9)
C(9)-C(8)-C(13)	128.1(8)
C(10)-C(9)-C(8)	107.1(9)
C(10)-C(9)-H(9)	126.5
C(8)-C(9)-H(9)	126.5
C(9)-C(10)-C(11)	124.8(9)
C(9)-C(10)-H(10)	117.6
C(11)-C(10)-H(10)	117.6
N(2)-C(11)-C(12)	106.8(8)
N(2)-C(11)-C(10)	124.8(8)
C(12)-C(11)-C(10)	128.3(8)
C(13)-C(12)-C(11)	105.4(9)
C(13)-C(12)-H(12)	127.3
C(11)-C(12)-H(12)	127.3
C(12)-C(13)-C(8)	126.3(9)
C(12)-C(13)-H(13)	116.9
C(8)-C(13)-H(13)	116.9
C(15)-C(14)-N(2)	92.2(8)
C(15)-C(14)-H(14A)	113.3
N(2)-C(14)-H(14A)	113.3
C(15)-C(14)-H(14B)	113.3
N(2)-C(14)-H(14B)	113.3
H(14A)-C(14)-H(14B)	110.6
C(14)-C(15)-O(2)	116.6(8)
C(14)-C(15)-H(15A)	108.1
O(2)-C(15)-H(15A)	108.1

C(14)-C(15)-H(15B)	108.1
O(2)-C(15)-H(15B)	108.1
H(15A)-C(15)-H(15B)	107.3
C(17)-C(16)-O(2)	96.1(9)
C(17)-C(16)-H(16A)	112.5
O(2)-C(16)-H(16A)	112.5
C(17)-C(16)-H(16B)	112.5
O(2)-C(16)-H(16B)	112.5
H(16A)-C(16)-H(16B)	110
C(16)-C(17)-N(2)	114.0(8)
C(16)-C(17)-H(17A)	108.7
N(2)-C(17)-H(17A)	108.7
C(16)-C(17)-H(17B)	108.7
N(2)-C(17)-H(17B)	108.7
H(17A)-C(17)-H(17B)	107.6
C(6)-N(1)-C(7)	119.3(7)
C(11)-N(2)-C(14)	99.3(7)
C(11)-N(2)-C(17)	119.8(7)
C(14)-N(2)-C(17)	127.9(7)
C(1)-O(1)-H(1O)	116(5)
C(16)-O(2)-C(15)	121.0(7)

Symmetry transformations used to generate equivalent atoms

Table S9 Anisotropic displacement parameters (Å^2 × 10^3) for the ligand (**HL**). The anisotropic displacement factor exponent takes the form: $-2\pi^2[h^2a^{*2}U^{11} + ... + 2 h k a^* b^* U^{12}]$.

**Atoms**	**U^{11}**	**U^{22}**	**U^{33}**	**U^{23}**	**U^{13}**	**U^{12}**
C(1)	8(3)	24(3)	48(4)	6(3)	10(3)	-1(2)
C(2)	32(6)	30(5)	44(6)	1(4)	23(5)	1(4)
C(3)	19(5)	38(6)	66(7)	-5(5)	10(5)	1(4)
C(4)	44(7)	29(6)	99(9)	-1(6)	-32(6)	-2(5)
C(5)	19(5)	48(6)	62(7)	3(5)	1(4)	9(4)
C(6)	8(3)	24(3)	48(4)	6(3)	10(3)	-1(2)
C(7)	18(5)	36(5)	36(5)	2(4)	5(4)	1(4)
C(8)	7(4)	34(5)	53(6)	5(5)	8(4)	3(4)
C(9)	24(5)	25(5)	44(6)	-4(4)	8(4)	-4(4)
C(10)	24(5)	38(5)	42(6)	-4(5)	5(4)	-8(4)
C(11)	8(4)	25(5)	47(6)	5(4)	5(4)	0(4)
C(12)	15(5)	34(5)	47(6)	-11(5)	9(4)	-3(4)
C(13)	26(6)	37(5)	70(7)	-9(5)	0(5)	1(4)
C(14)	14(5)	53(6)	56(6)	-4(5)	11(4)	8(4)
C(15)	24(6)	47(7)	97(9)	-1(6)	-6(6)	1(5)
C(16)	31(6)	47(7)	108(10)	-5(6)	14(6)	10(5)
C(17)	21(5)	38(5)	49(6)	-4(5)	28(4)	5(4)
N(1)	24(4)	38(5)	41(5)	-3(4)	-1(3)	-7(3)
N(2)	21(4)	40(5)	50(5)	-14(4)	1(4)	4(3)
O(1)	23(4)	58(4)	71(5)	-22(4)	0(3)	-13(3)
O(2)	7(3)	74(5)	111(6)	-14(5)	8(4)	1(3)

Table S10 Hydrogen coordinates (× 10^4) and isotropic displacement parameters ($Å^2$ × 10^3) for the ligand (**HL**).

**Atoms**	**x**	**y**	**z**	**U(eq)**
H(2)	1133	8053	6642	42
H(3)	52	6977	5467	49
H(4)	422	5898	4114	70
H(5)	2295	6012	3599	52
H(7)	3463	5353	3862	36
H(9)	5245	7933	4858	37
H(10)	6792	8274	4257	41
H(12)	6178	4895	2095	38
H(13)	4604	4640	2704	53
H(14A)	7874	8346	3800	49
H(14B)	8339	6442	3886	49
H(15A)	9622	8078	3297	68
H(15B)	8784	8856	2604	68
H(16A)	8442	7165	1112	74
H(16B)	8880	5247	1095	74
H(17A)	7886	4473	2148	42
H(17B)	7122	5540	1503	42
H(1O)	3940(110)	8420(160)	6530(80)	110(40)

Table S11 Torsion angles [°] for for the ligand (**HL**).

Positions of atoms	Torsion angles [°]
O(1)-C(1)-C(2)-C(3)	179.9(6)
C(6)-C(1)-C(2)-C(3)	-1.0(11)
C(1)-C(2)-C(3)-C(4)	1.4(15)
C(2)-C(3)-C(4)-C(5)	-3.2(16)
C(3)-C(4)-C(5)-C(6)	3.5(11)
C(4)-C(5)-C(6)-N(1)	-177.9(7)
C(4)-C(5)-C(6)-C(1)	-4.6(13)
C(2)-C(1)-C(6)-N(1)	176.6(9)
O(1)-C(1)-C(6)-N(1)	-4.7(13)
C(2)-C(1)-C(6)-C(5)	3.8(15)
O(1)-C(1)-C(6)-C(5)	-177.6(9)
N(1)-C(7)-C(8)-C(9)	7.8(13)
N(1)-C(7)-C(8)-C(13)	-169.2(8)
C(7)-C(8)-C(9)-C(10)	-175.5(9)
C(13)-C(8)-C(9)-C(10)	1.3(13)
C(8)-C(9)-C(10)-C(11)	-0.9(13)
C(9)-C(10)-C(11)-N(2)	175.4(10)
C(9)-C(10)-C(11)-C(12)	-0.3(15)
N(2)-C(11)-C(12)-C(13)	-175.1(8)
C(10)-C(11)-C(12)-C(13)	1.2(13)
C(11)-C(12)-C(13)-C(8)	-0.8(13)
C(7)-C(8)-C(13)-C(12)	176.0(10)
C(9)-C(8)-C(13)-C(12)	-0.4(15)
N(2)-C(14)-C(15)-O(2)	-49.9(10)
O(2)-C(16)-C(17)-N(2)	46.5(10)
C(5)-C(6)-N(1)-C(7)	-23.2(11)
C(1)-C(6)-N(1)-C(7)	161.6(7)
C(8)-C(7)-N(1)-C(6)	173.3(9)
C(12)-C(11)-N(2)-C(14)	-156.1(7)
C(10)-C(11)-N(2)-C(14)	27.4(10)
C(12)-C(11)-N(2)-C(17)	-11.9(10)
C(10)-C(11)-N(2)-C(17)	171.6(7)
C(15)-C(14)-N(2)-C(11)	-171.9(7)
C(15)-C(14)-N(2)-C(17)	48.1(11)
C(16)-C(17)-N(2)-C(11)	171.0(9)
C(16)-C(17)-N(2)-C(14)	-56.0(13)
C(17)-C(16)-O(2)-C(15)	-61.2(11)
C(14)-C(15)-O(2)-C(16)	72.9(12)

Symmetry transformations used to generate equivalent atoms

References

1. K. Sakthikumar, J. Dhaveethu Raja, R. Vijay Solomon, M. Sankarganesh, Density functional theory molecular modelling, DNA interactions, antioxidant, antimicrobial, anticancer and biothermodynamic studies of bioactive water-soluble mixed ligand complexes, J. Biomol. Struct.37 (2018) 2498–2514, http://dx.doi.org/10.1080/07391102.2018.1492970.
2. K. Sakthikumar, R.V. Solomon, J.D. Raja, Spectro-electrochemical assessments of DNA/BSA interactions, cytotoxicity, radical scavenging and pharmacological implications of biosensitive and biologically active morpholine-based metal(II) complexes: a combined experimental and computational investigation, RSC Advances. 9 (2019) 14220–14241, http://dx.doi.org/10.1039/c8ra09218d.
3. Y.M. Song, Q. Wu, P.J. Yang, N.N. Luan, L.F. Wang, Y.M. Liu, DNA Binding and cleavage activity of Ni(II) complex with all-trans retinoic acid, J. Inorg. Biochem. 100 (2006) 1685–1691, http://dx.doi.org/10.1016/j.jinorgbio.2006.06.00.
4. K. Sakthikumar, J.D. Raja, M. Sankarganesh, J. Rajesh, Antimicrobial, Antioxidant and DNA Interaction Studies of Water-soluble Complexes of Schiff Base Bearing Morpholine Moiety, Indian J. Pharm. Sci. 80 (2018) 723–738, http://dx.doi.org/10.4172/pharmaceutical-sciences.1000413.
5. E.S. Aazam, A.F. EL Husseiny, H.M. Al-Amri, Synthesis and photoluminescent properties of a Schiff-base ligand and its mononuclear Zn(II), Cd(II), Cu(II), Ni(II) and Pd(II)metal complexes, Arab. J. Chem. 5 (2012) 45–53, http://dx.doi.org/10.1016/j.arabjc.2010.07.022.
6. N. Raman, S. Ali, D. Raja, Designing, synthesis and spectral characterization of Schiff base transition metal complexes: DNA cleavage and antimicrobial activity studies, J. Serb. Chem. Soc. 73 (2008) 1063–1071l, http://dx.doi.org/10.2298/jsc0811063r.

7. Y. Li, Z.Y. Yang, Z.C. Liao, Z.C. Han, Z.C. Liu, Synthesis, crystal structure, DNA binding properties and antioxidant activities of transition metal complexes with 3-carbaldehyde-chromone semicarbazone, Inorg. Chem. Commun. 13 (2010)1213–1216, https://doi.org/10.1016/j.inoche.2010.07.005.

8. G.D. Dimitrov, M.S. Atanassova, Synthesis and Spectroscopic Characterization of a Complex of 1, 10-Phenanthroline with Magnesium, Z. anorg. allg. Chem. 629 (2003) 12–14, https://doi.org/10.1002/zaac.200390005.

9. M. Shakir, A. Abbasi, M. Azam, A.U. Khan, Synthesis, spectroscopic studies and crystal structure of the Schiff base ligand L derived from condensation of 2-thiophenecarboxaldehyde and 3,3′-diaminobenzidine and its complexes with Co(II), Ni(II), Cu(II), Cd(II) and Hg(II): Comparative DNA binding studies of L and its Co(II), Ni(II) and Cu(II) complexes, Spectrochim. Acta A Mol. Biomol. 79 (2011) 1866–1875, http://dx.doi.org/10.1016/j.saa.2011.05.077.

10. N. Raman, Y.P. Raja, A. Kulandaisamy, Synthesis and characterisation of Cu(II), Ni(II), Mn(II), Zn(II) and VO(II) Schiff base complexes derived fromo-phenylenediamine and acetoacetanilide, J. Chem. Sci. 113 (2001) 183–189, https://doi.org/10.1007/bf02704068.

11. A.B.P Lever, Inorganic Electronic Spectroscopy, second ed., Elsevier, Amsterdam, Foundations of Electron Spin Resonance, Academic Press, New York, (1978) 1-420.

12. T. Sato, T. Nakamura, The complexes formed in the divalent transition metal-sulphuric acid-di-(2-ethylhexyl)-phosphoric acid extraction systems-cobalt(II), nickel(II) and copper(II) complexes, J. inorg. nucl. Chem. 34 (1972) 3721–3730, https://doi.org/10.1016/0022-1902(72)80017-4.

13. A. B. P. Lever, Electronic spectra of some transition metal complexes: Derivation of Dq and B, J. Chem. Educ. 45 (1968) 711, https://doi.org/10.1021/ed045p711.

14. A. Kumar Singh, R. Mukherjee, Structure and properties of bivalent nickel and copper complexes with pyrazine-amide-thioether coordination: stabilization of trivalent nickel, Dalton Trans.17 (2005) 2886, https://doi.org/10.1039/b504893a.

15. F. Blasco, L. Perelló, J. Latorre, J. Borrás, S. Garciá-Granda, Cobalt(II), Nickel(II), and Copper(II) complexes of sulfanilamide derivatives: Synthesis, spectroscopic studies, and antibacterial activity. Crystal structure of [Co(sulfacetamide)2(NCS)2], J. Inorg. Biochem. 61 (1996)143–154, https://doi.org/10.1016/0162-0134(95)00053-4.

16. P.K. Panchal, H.M. Parekh, P.B. Pansuriya, M.N. Patel, Bactericidal activity of different oxovanadium(IV) complexes with schiff bases and application of chelation theory, J. Enzyme Inhib. Med. Chem. 21 (2006) 203-209, https://doi.org/10.1080/14756360500535229.

17. W. Gurnule, P. Rahangdale, L. Paliwal, R. Kharat, Synthesis, characterization and ion-exchange properties of 4-hydroxyacetophenone, biuret and formaldehyde terpolymer resins, React. Funct. Polym. 55 (2003) 255–265, http://dx.doi.org/10.1016/s1381-5148(03)00016-6.

18. P.P. Kalbende, M.V. Tarase, A.B. Zade, Preparation, Characterization, and Thermal Degradation Studies of p-Nitrophenol-Based Copolymer, J. Chem. 2013 (2013) 1–9, https://doi.org/10.1155/2013/846327.

19. M.G. Miguel, S. Nunes, S.A. Dandlen, A.M. Cavaco, M.D. Antunes, Phenols, flavonoids and antioxidant activity of aqueous and methanolic extracts of propolis (Apis mellifera L.) from Algarve, South Portugal, Food. Sci. Tech (Campinas). 34 (2014) 16–23, http://dx.doi.org/10.1590/s0101-20612014000100002.

20. Boora, E. Chirisa, S. Mukanganyama, Evaluation of Nitrite Radical Scavenging Properties of Selected Zimbabwean Plant Extracts and Their Phytoconstituents, J. Food Proc. 2014 (2014) 1–7, http://dx.doi.org/10.1155/2014/918018.

21. S. Shujah, N. Khalid, S. Ali, Homobimetallic organotin(IV) complexes with succinohydrazide Schiff base: Synthesis, spectroscopic characterization and biological screening, Russ. J. Gen. Chem. 87 (2017) 515–522, http://dx.doi.org/10.1134/s1070363217030227.

22. M. Balouiri, M. Sadiki, S.K. Ibnsouda, Methods for in vitro evaluating antimicrobial activity: A review, J. Pharm. Analysis. 6 (2016) 71–79, http://dx.doi.org/10.1016/j.jpha.2015.11.005.

23. I. Ali, W.A. Wani, K. Saleem, M.F. Hsieh, Anticancer metallo drugs of glutamic acid sulphonamides: in silico, DNA binding, hemolysis and anticancer studies, RSC Adv. 4 (2014) 29629–29641, http://dx.doi.org/10.1039/c4ra02570a.

Supplementary Figures

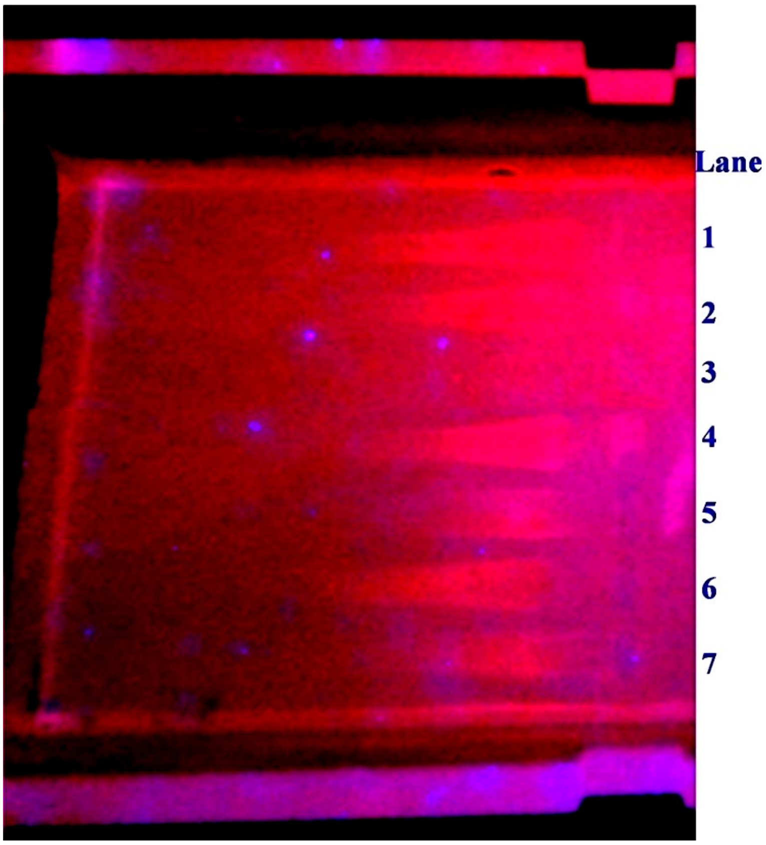

Fig. S13. Raw data for electrophoretic gels and blots showing the chemical nuclease activity of CT-DNA by the synthesized ligand (**HL**) and its complexes (**1–3**) in the presence of hydrogen peroxide. Lane: 1 DNA alone + H_2O_2; Lane: 2 ligand **(HL)** + DNA + H_2O_2 ; Lane: 4 complex (**1**) + DNA + H_2O_2; Lane: 5 complex (**2**) + DNA + H_2O_2; Lane: 6 complex (**3**) + DNA + H_2O_2 excluding Lanes: 3 and 7, which belongs to another scheme.

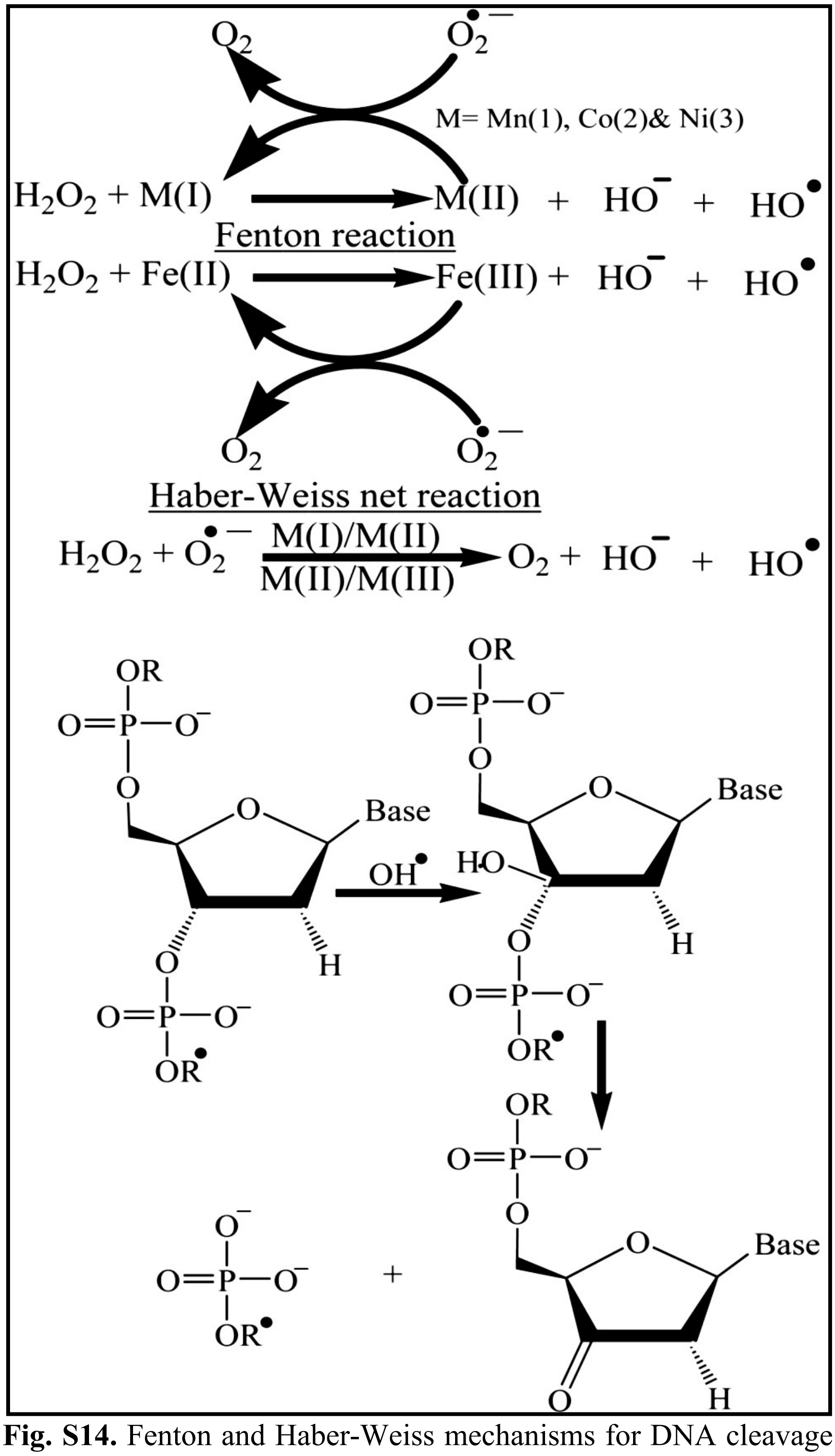

Fig. S14. Fenton and Haber-Weiss mechanisms for DNA cleavage in the H_2O_2 environment.

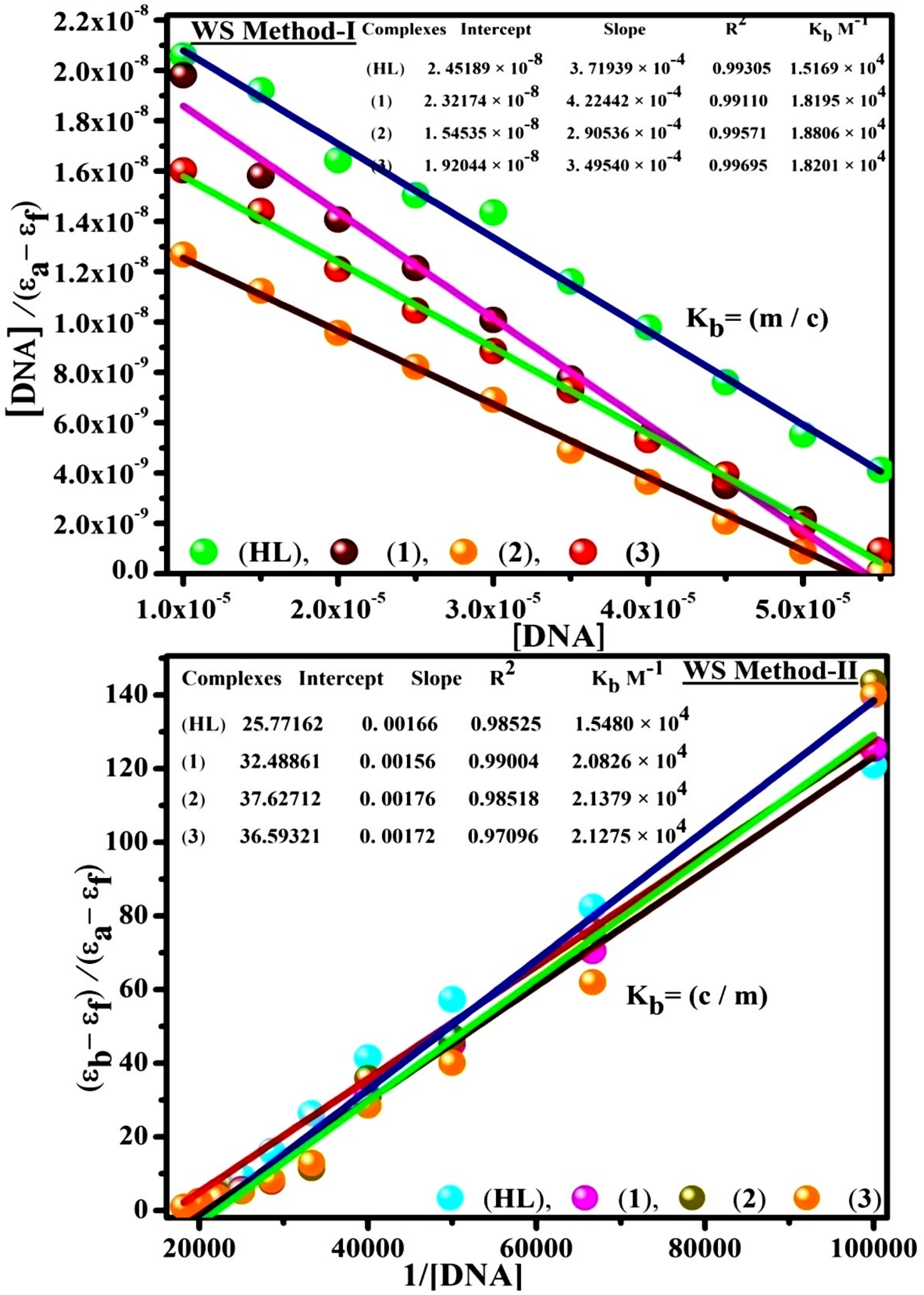

Fig.S15. Linear plots of {[DNA] / (ε_a–ε_f)} *vs* [DNA] and {(ε_b–ε_f)/ (ε_a–ε_f)} *vs* [DNA] M^{-1} by Wolfe-Shimmer methods (I & II) for the estimation of the intrinsic DNA binding constants (K_b) for ligand (**HL**) and complexes (**1**–**3**).

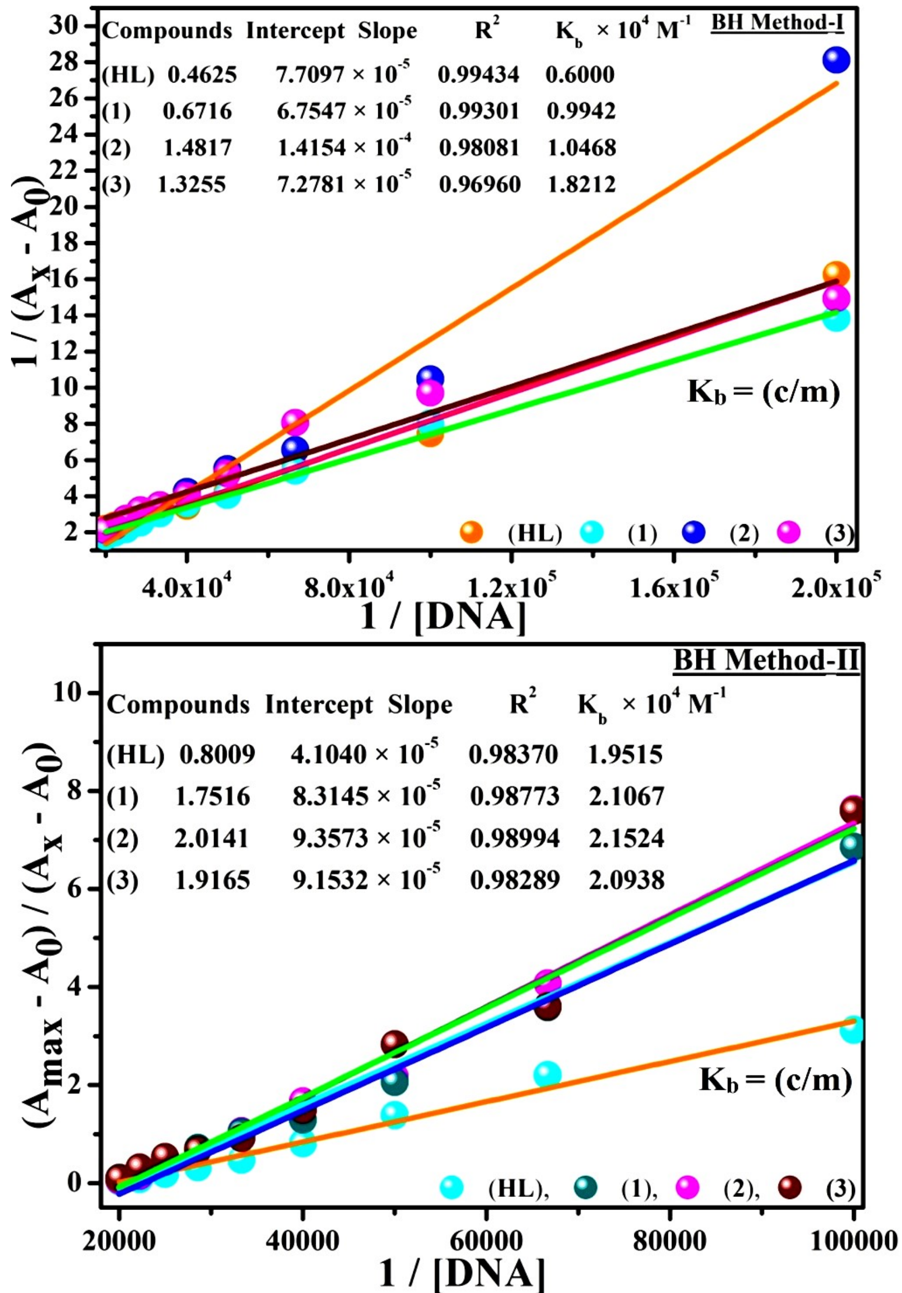

Fig.S16. Linear plots of [1 / (A_x – A_0)] *vs* {1 / [DNA]} M^{-1} and [(A_{max}–A_0) / (A_x – A_0)] *vs* {1 / [DNA]} M^{-1} by Benesi-Hildebrand (I & II) methods for the estimation of the intrinsic DNA binding constants (K_b) for ligand (**HL**) and complexes (**1–3**).

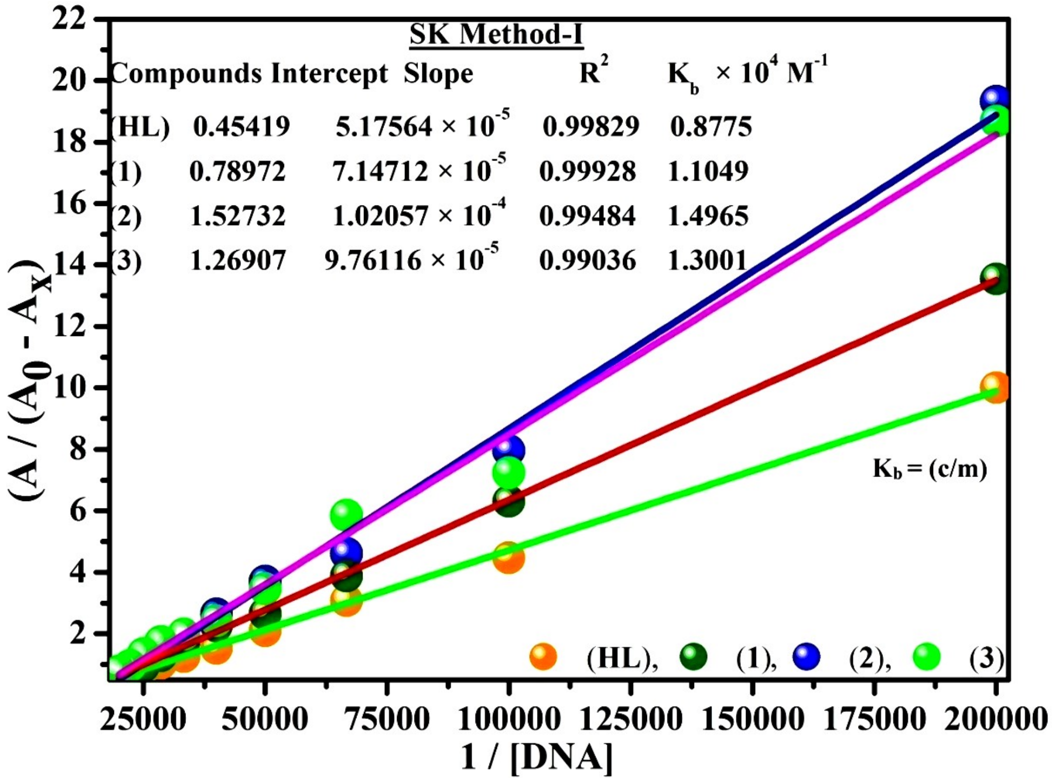

Fig.S17. Linear plot of [(A / (A_0–A)] *vs* {1 / [DNA]} M^{-1} by Sakthi-Krause method I for the estimation of the intrinsic DNA binding constants (K_b) for ligand (**HL**) and complexes (**1** –**3**).

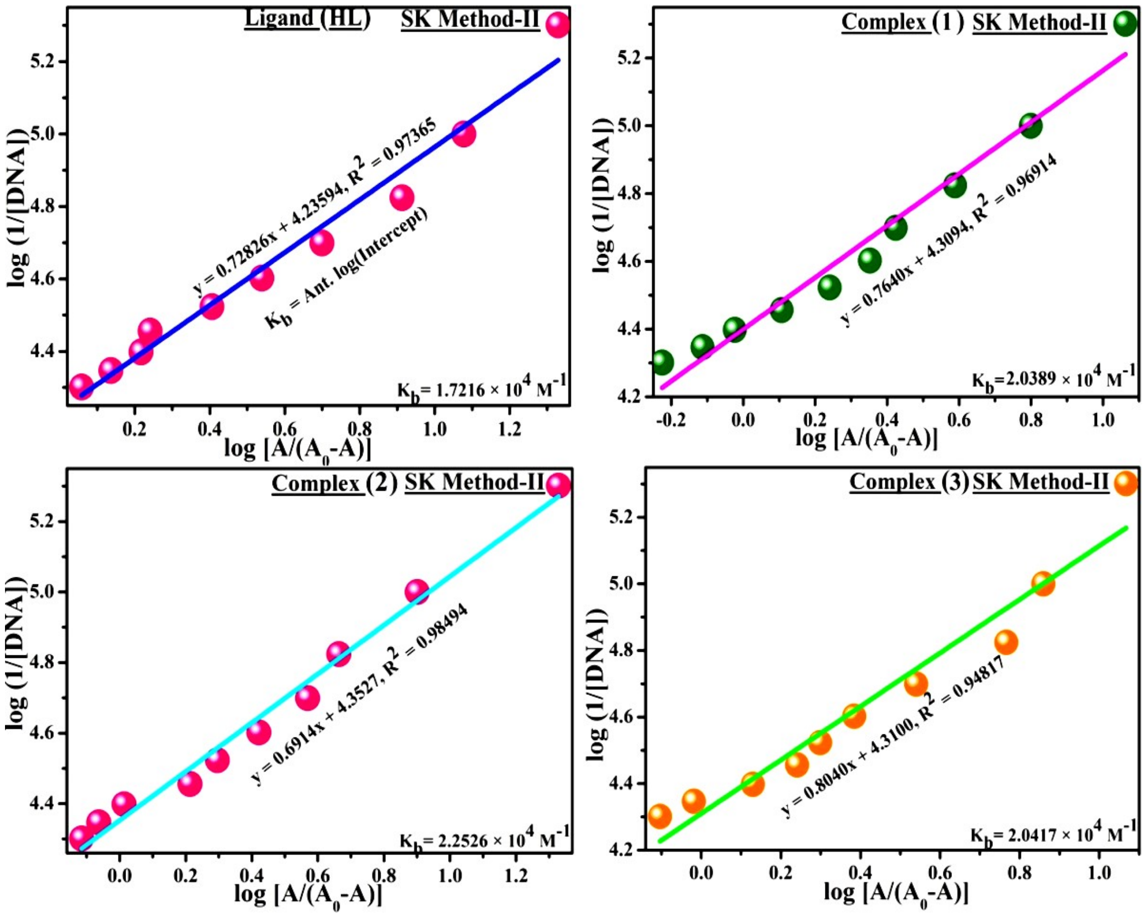

Fig.S18. Linear plot of log{1 / [DNA]} M^{-1} *vs* log [(A / (A_0–A)] by Sakthi-Krause method II for the determination of the intrinsic DNA binding constant (K_b) for ligand (**HL**) and complexes (**1**–**3**).

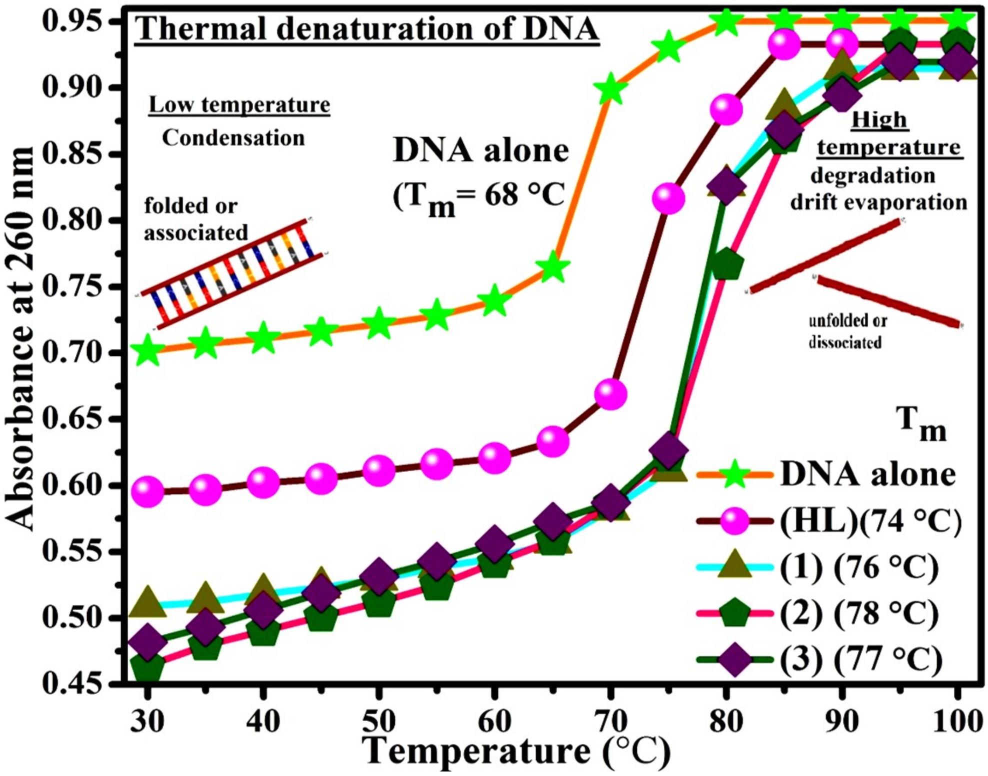

Fig.S19. DNA thermal denaturation profile at 260 nm in the absence and presence of compounds in 5mM Tris-HCl /50 mM NaCl buffer pH = 7.2, [DNA] / [Complex] = 1(R). The denaturation temperature (T_m) was taken as the mid-point of the hyperchromic transition.

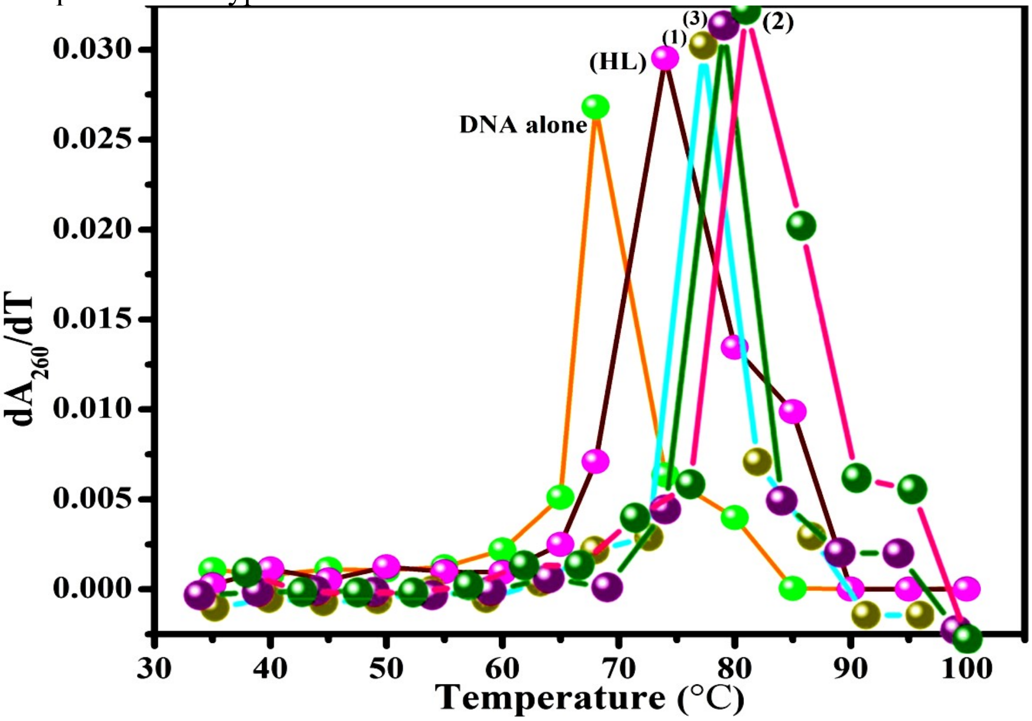

Fig.S19a. Derivative melting curve for DNA thermal denaturation at 260 nm in the absence and presence of compounds in 5mM Tris-HCl /50 mM NaCl buffer pH = 7.2, [DNA] / [Complex] = 1(R)

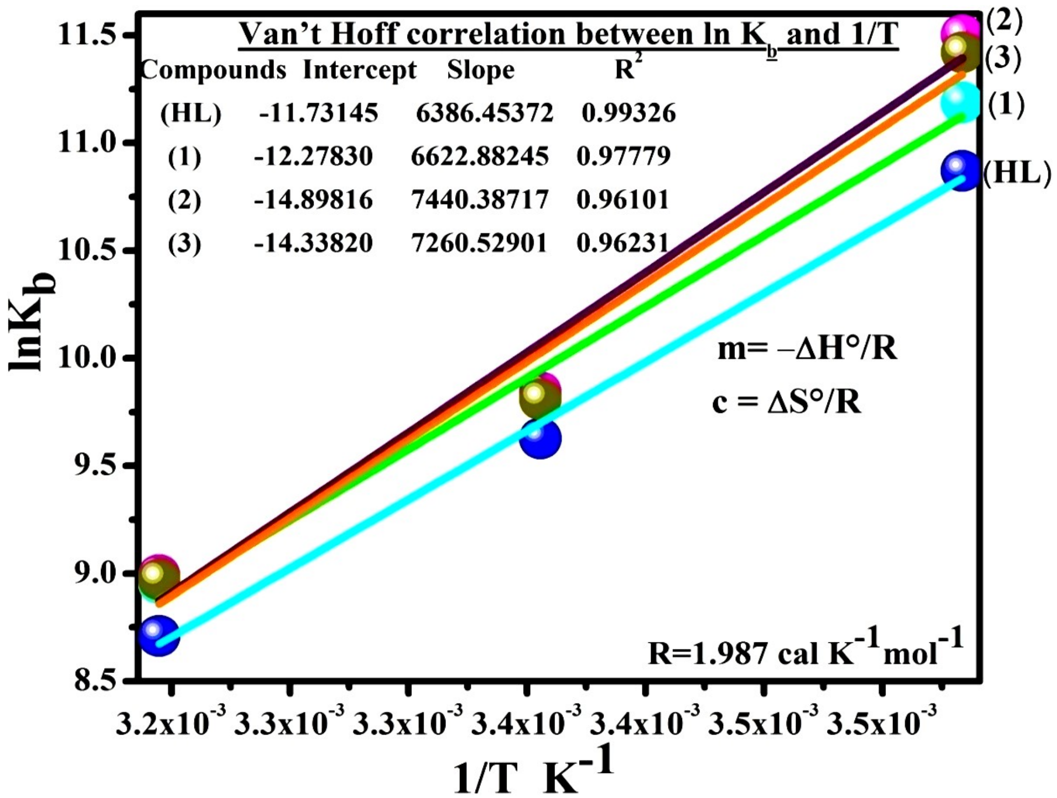

Fig. S20. The van't Hoff plot of ln K_b versus 1/T (K^{-1}) for the binding of test compounds to CT-DNA. The slope (m) and intercept (c) of the plot are equal to −ΔH∘/R and ΔS∘/R, respectively.

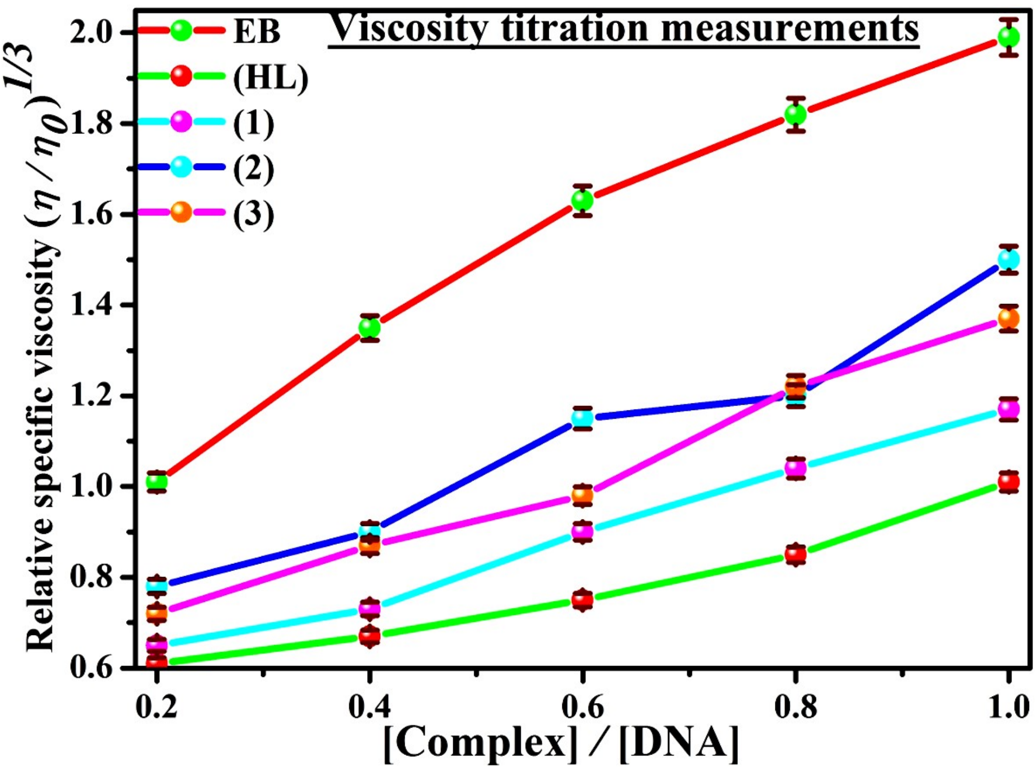

Fig.S21. Relative specific viscosities of CT-DNA in the presence of increasing amounts of ligand (**HL**), mixed ligand complexes (**1**–**3**) and Ethidium bromide (**EB**) at 25 °C in 5mM Tris-HCl buffer pH = 7.2 and Error limit ± 2.0 %.

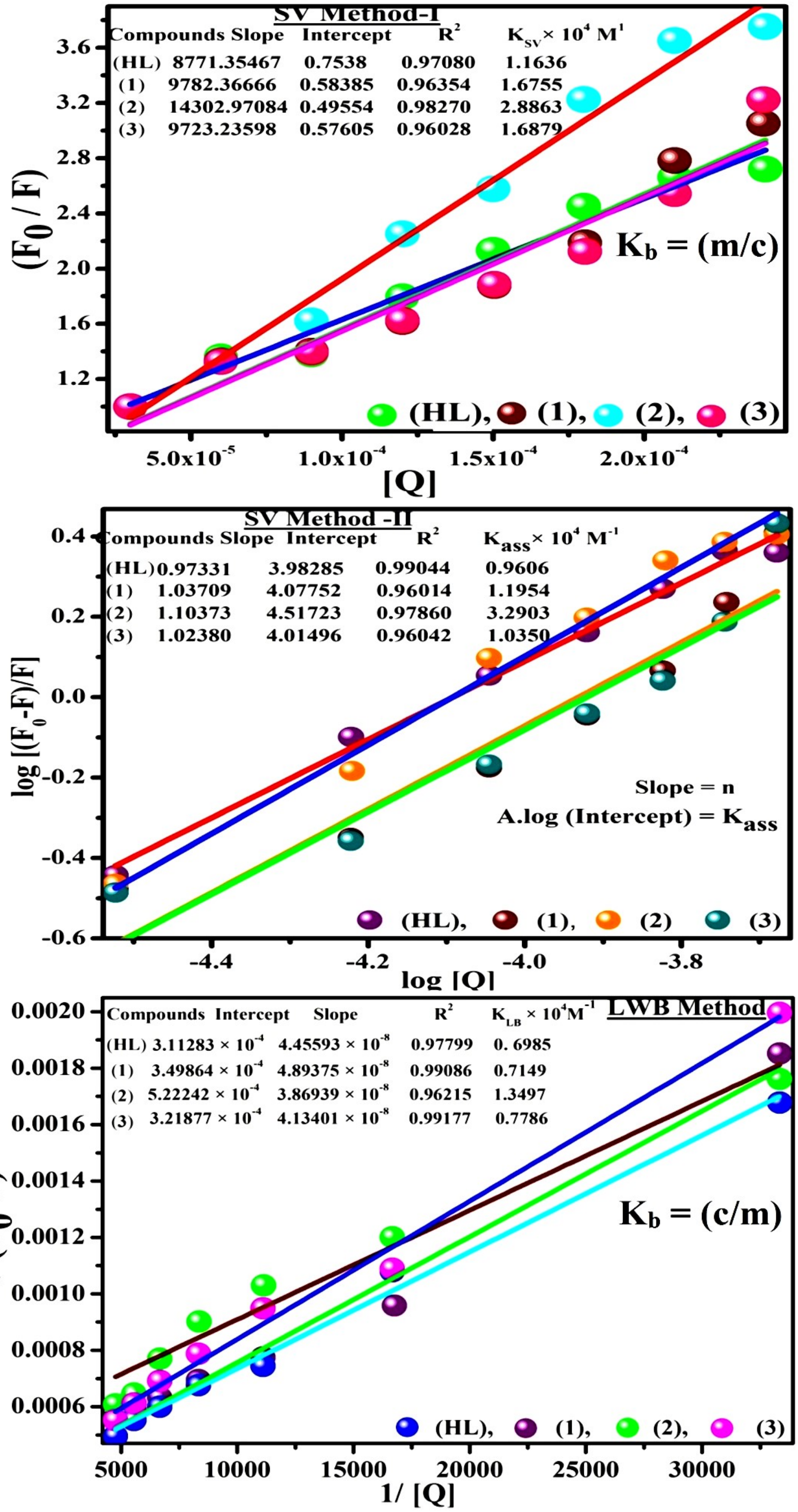

Fig.S22. Stern-Volmer linear plots of F_0 / F *vs* [Q] (Method I), log [(F_0–F) / F] *vs* log [Q] (SV Method-II) and Lineweaver-Burk linear plot of 1/ (F_0–F) *vs* 1/ [Q] for the quenching of fluorescence of ethidium bromide (EB)-DNA complex caused by ligand (**HL**) and mixed ligand complexes (**1**–**3**).

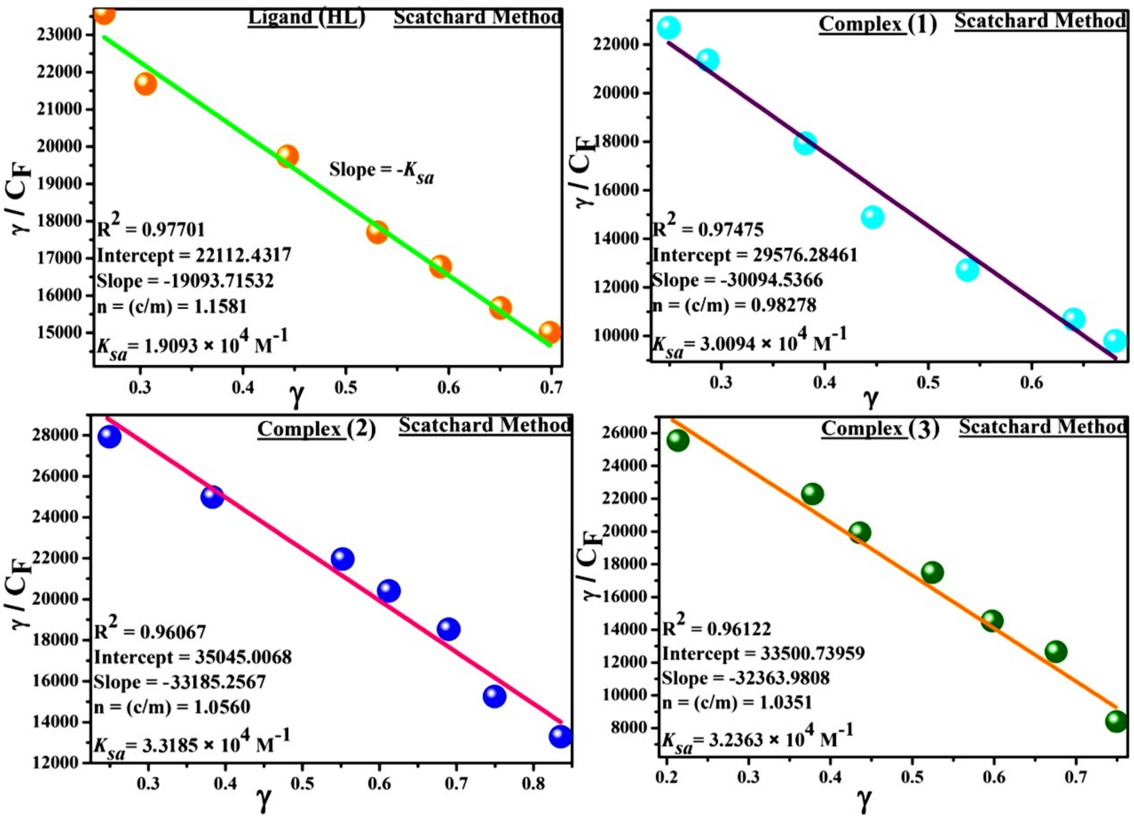

Fig.S23. Determining association binding constant and number of binding sites for ligand (**HL**) and complexes (**1–3**) – DNA by Scatchard linear plot of (γ / C_F) *vs* γ.

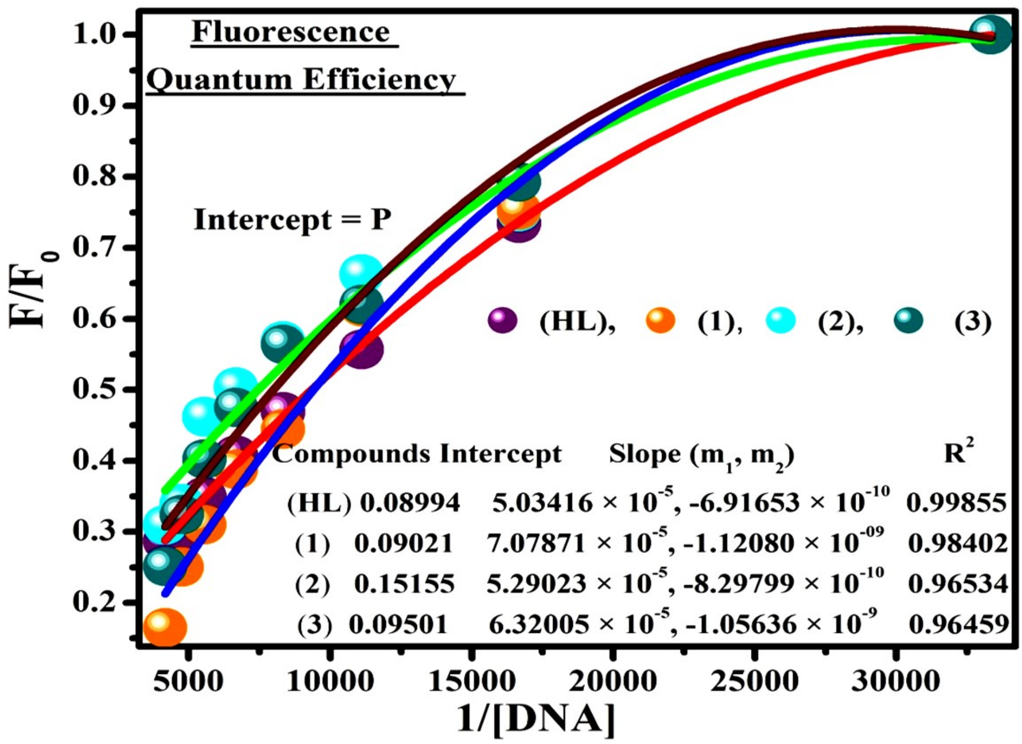

Fig.S24. Determination of ratio of the fluorescence quantum efficiency (P) of DNA bound and free complex ($P = \phi_b / \phi_f$) for ligand (**HL**) and complexes (**1**–**3**) from Stern-Volmer linear plots F/F0 *vs* 1/[DNA].

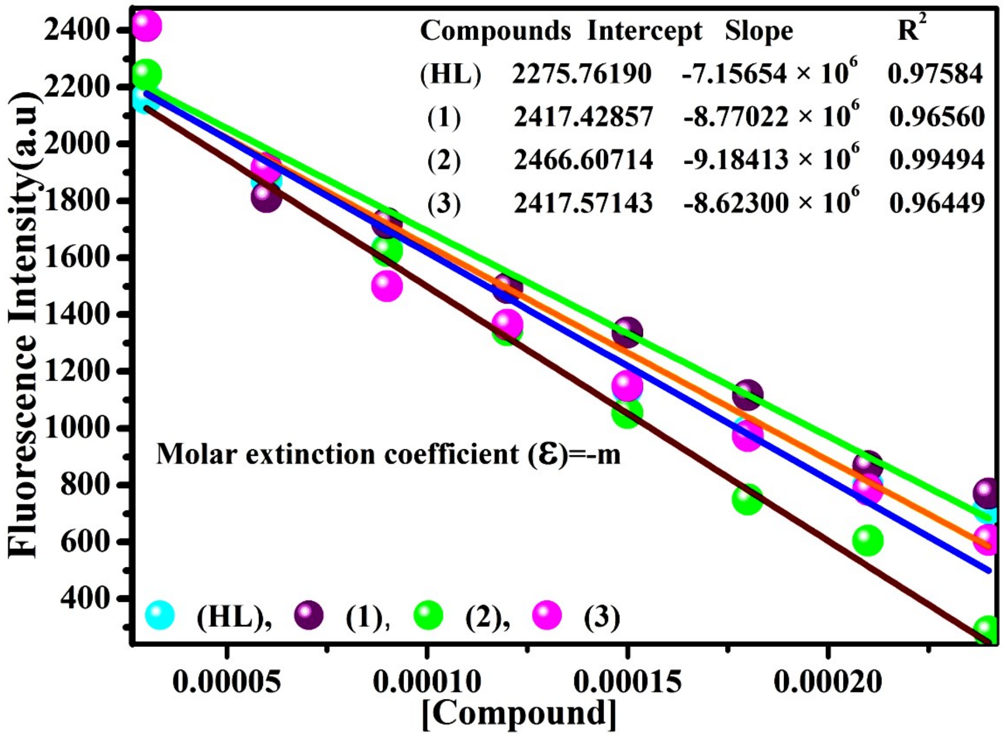

Fig.S25. Determination of apparent binding constant (K_{app}) from the linear plot of fluorescence intensity *vs* concentration of compounds. $K_{app} = K_{EB}\{\frac{[EB]}{[compound]}\}$; Where, $K_{EB} = 10^7$ M^{-1} at the concentration of 50 μM EB. IC_{50} value of complex concentration was measured at a 50 % reduction of the fluorescence intensity of EB.

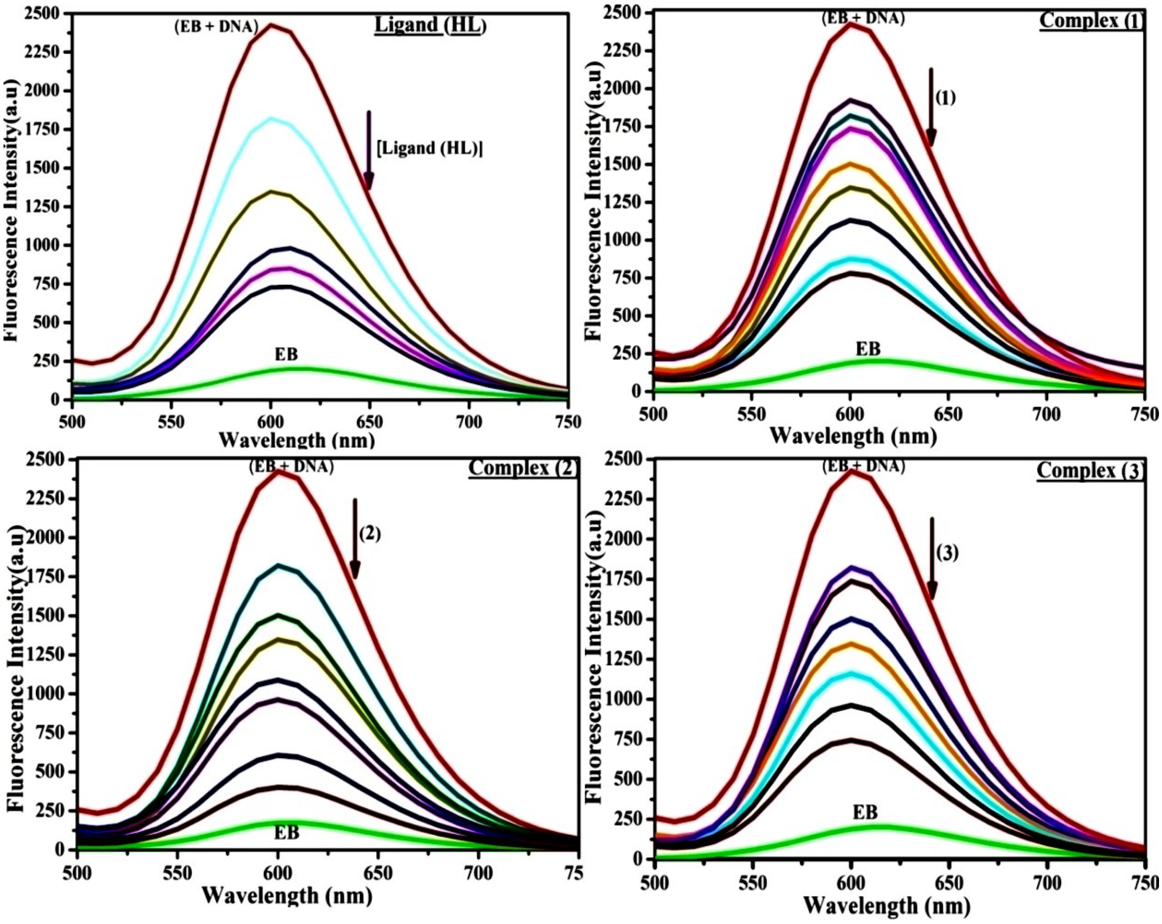

Fig. S26. Fluorescence quenching curve of **EB** bound DNA in the presence of ligand (**HL**) and complexes (**1**–**3**).

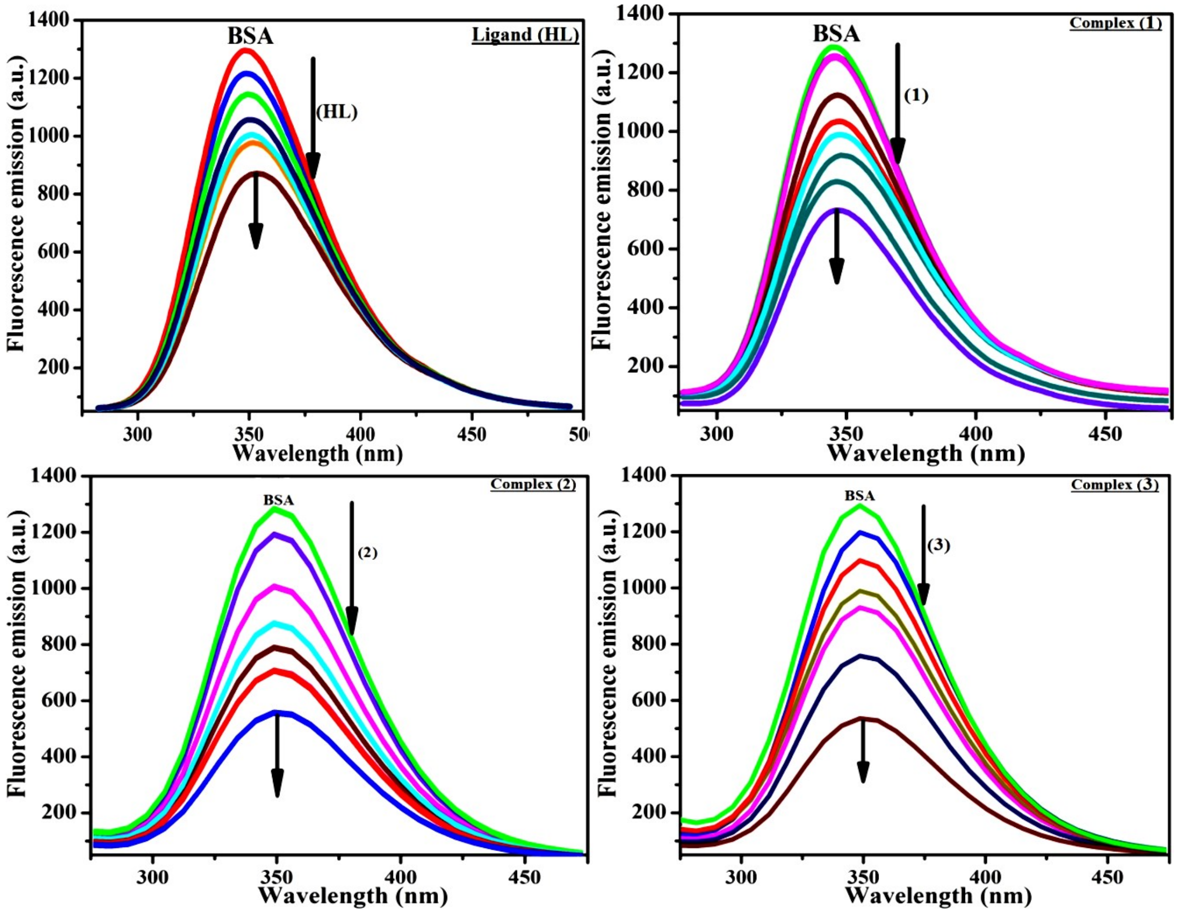

Fig. S27. Fluorescence quenching spectra of BSA in presence of various concentrations of ligand (**HL**) and complex (**1**–**3**).

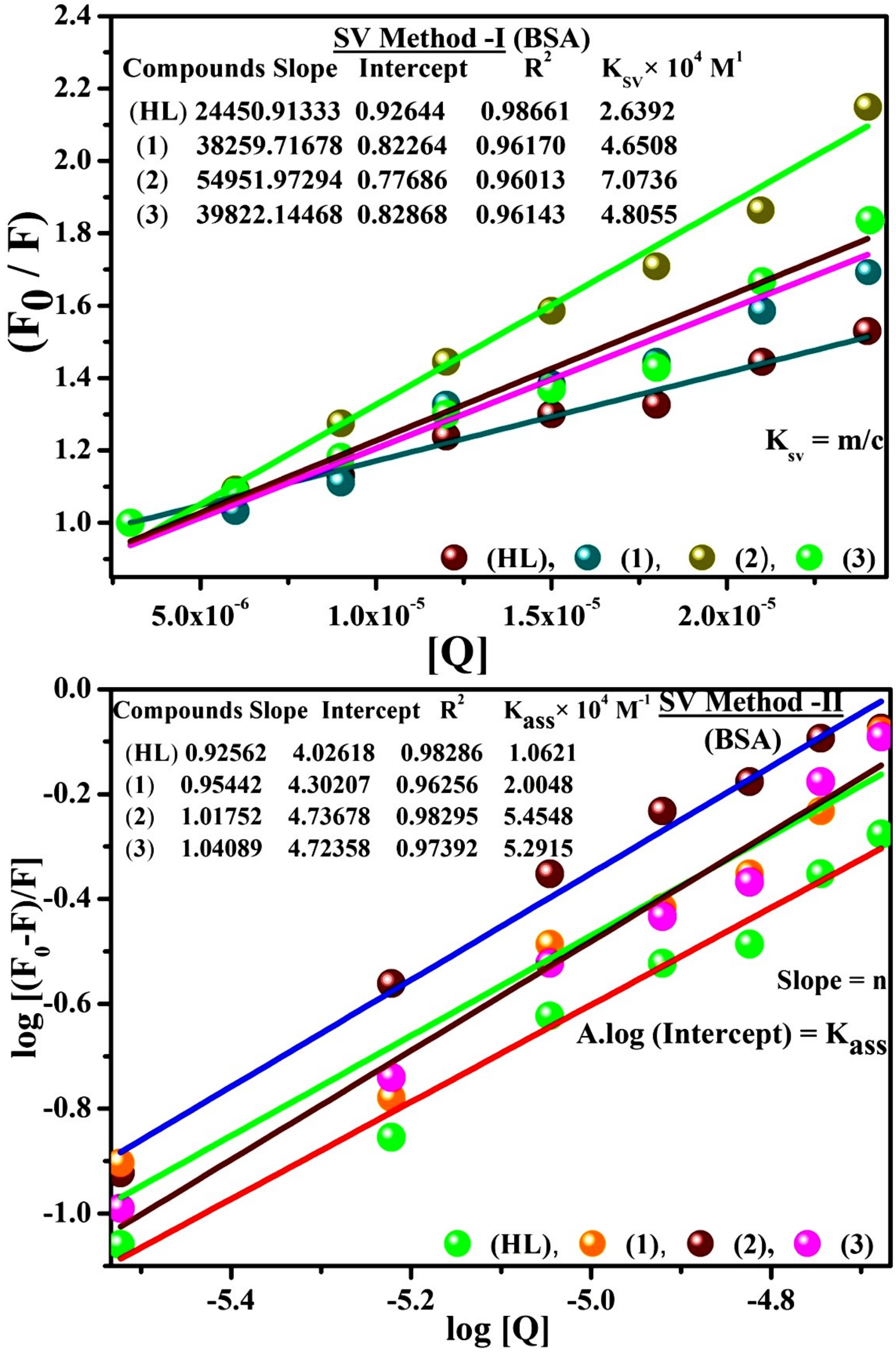

Fig.S28. Stern-Volmer linear plots of F_0 / F *vs* [Q] (Method I), and log [(F_0–F) / F] *vs* log [Q] (SV Method II) for the fluorescence quenching of **BSA** by free ligand (**HL**) and complexes (**1**–**3**).

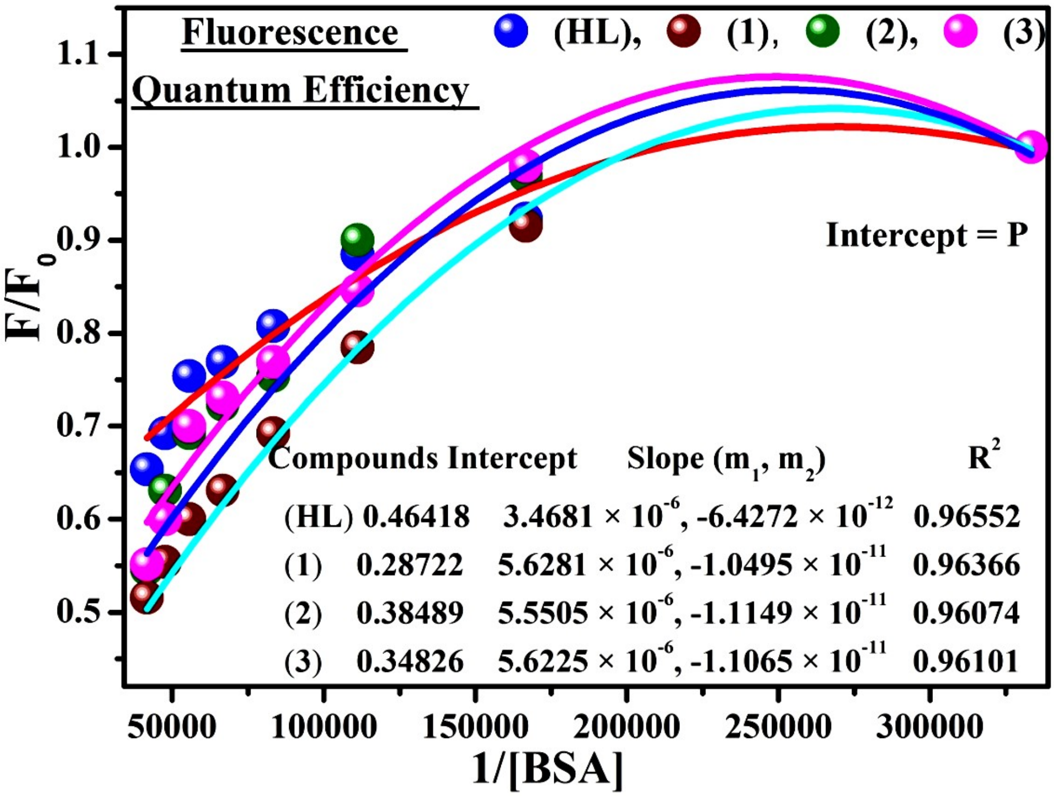

Fig.S29. Determination of ratio of the fluorescence quantum efficiency (P) of BSA bound and free complex (P = ϕ_b /ϕ_f) for ligand (**HL**) and mixed ligand complexes (**1**–**3**) from Stern-Volmer linear plots F/F0 *vs* 1/[BSA].

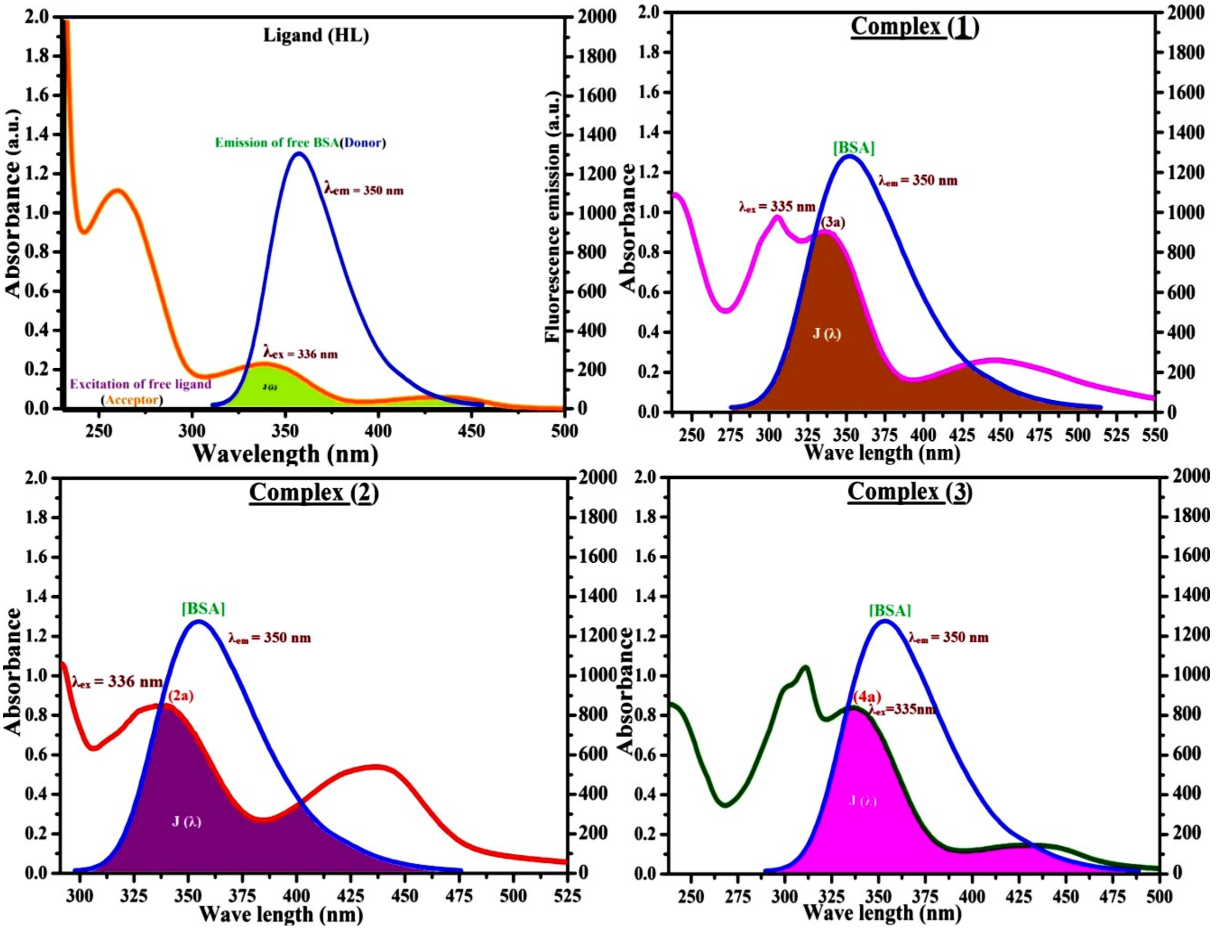

Fig.S30. The overlap of UV–Vis spectra of ligand (**HL**) & mixed ligand complexes (**1–3**) (**Acceptor**) at 335 – 336 nm with fluorescence emission spectrum of BSA (**Donor**) at 350 nm.

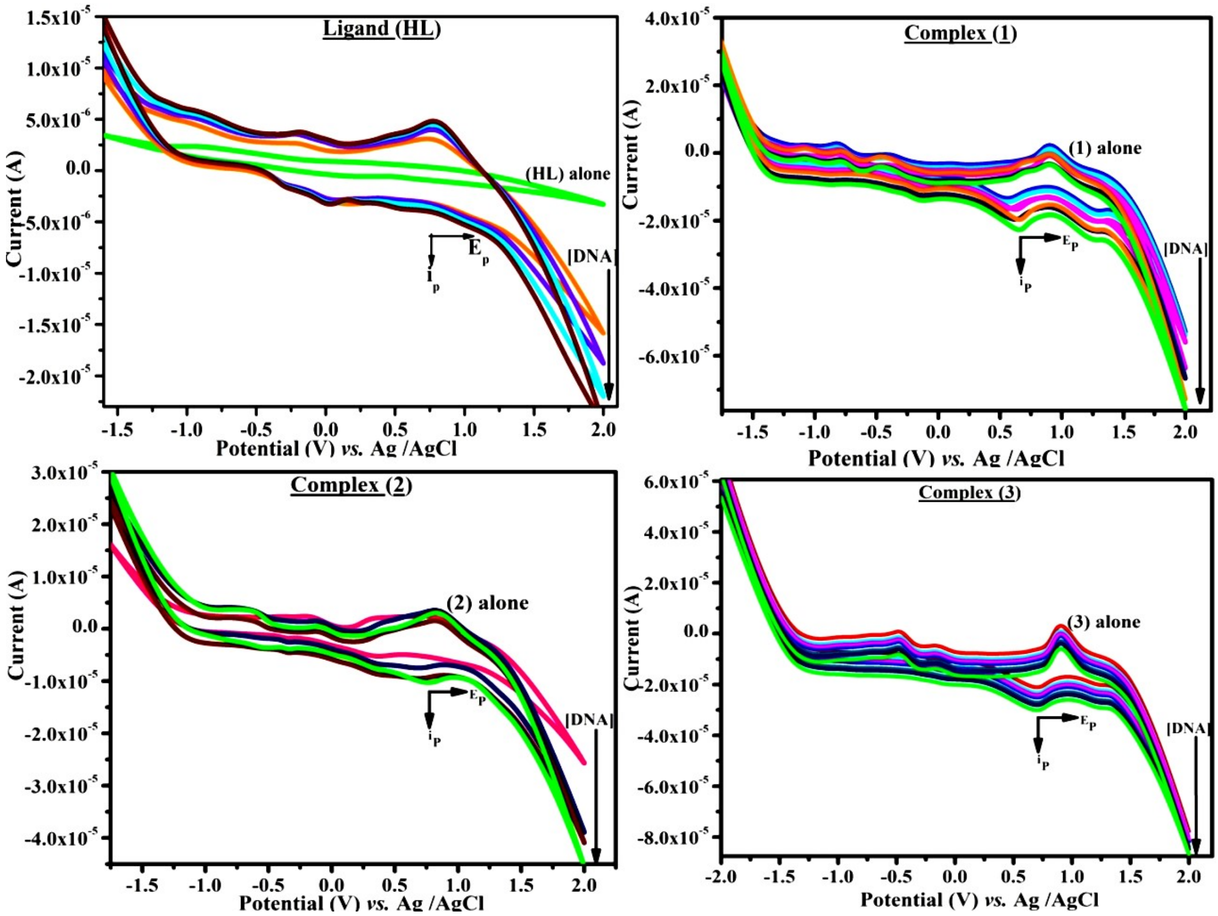

Fig.S31. Cyclic voltammograms of ligand (**HL**) and its ligand complexes (**1**–**3**) in 0.05 M Tris-HCl buffer (Supporting electrolyte) pH = 7.2 at 25 °C in presence of increasing concentration of DNA (10 – 50 μM) and arrow indicates the changes in peak current and potentials at scan rate 100 mvs^{-1}.

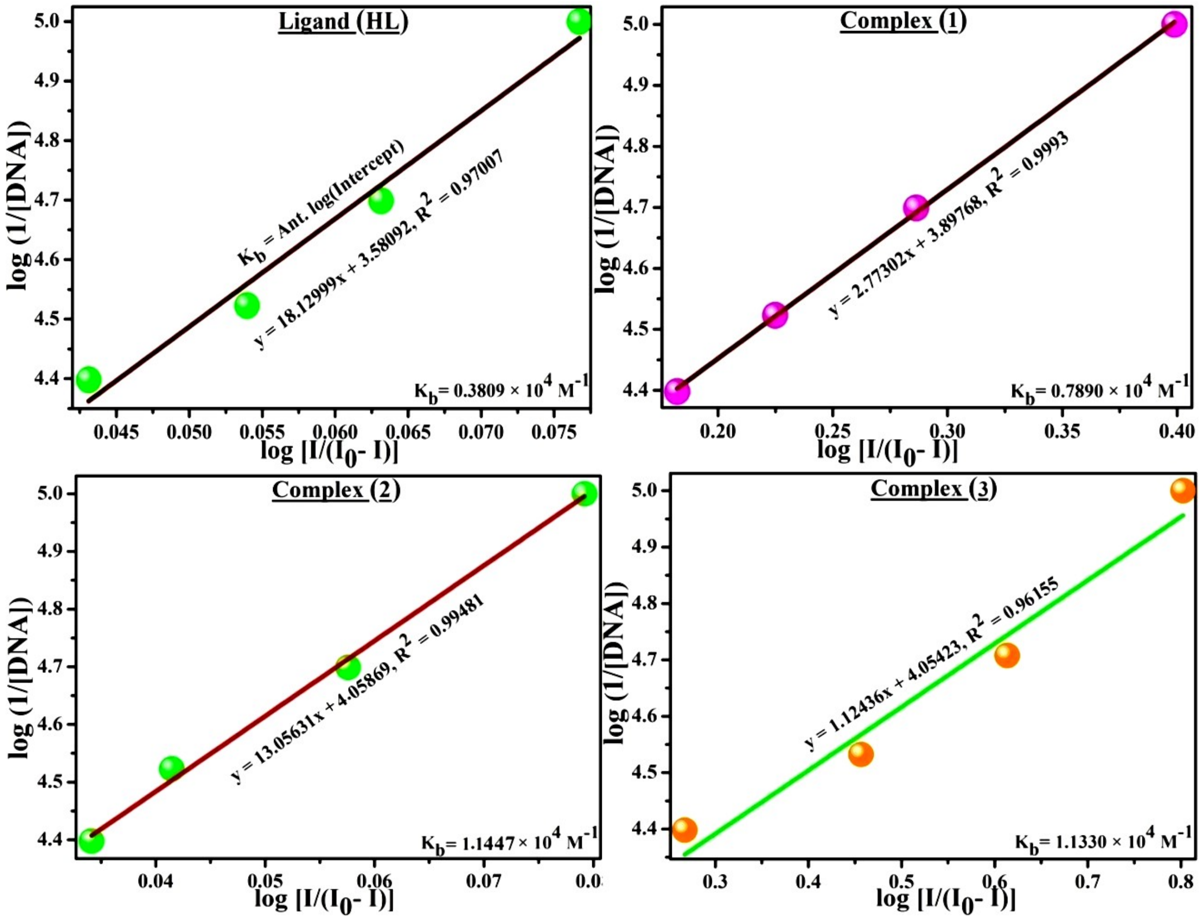

Fig.S32. Plot of log (1/[DNA]) *vs* log ($I/(I_0–I)$) to evaluate the binding constants for ligand (**HL**) and ligand complexes (**1**–**3**) with DNA by Method I.

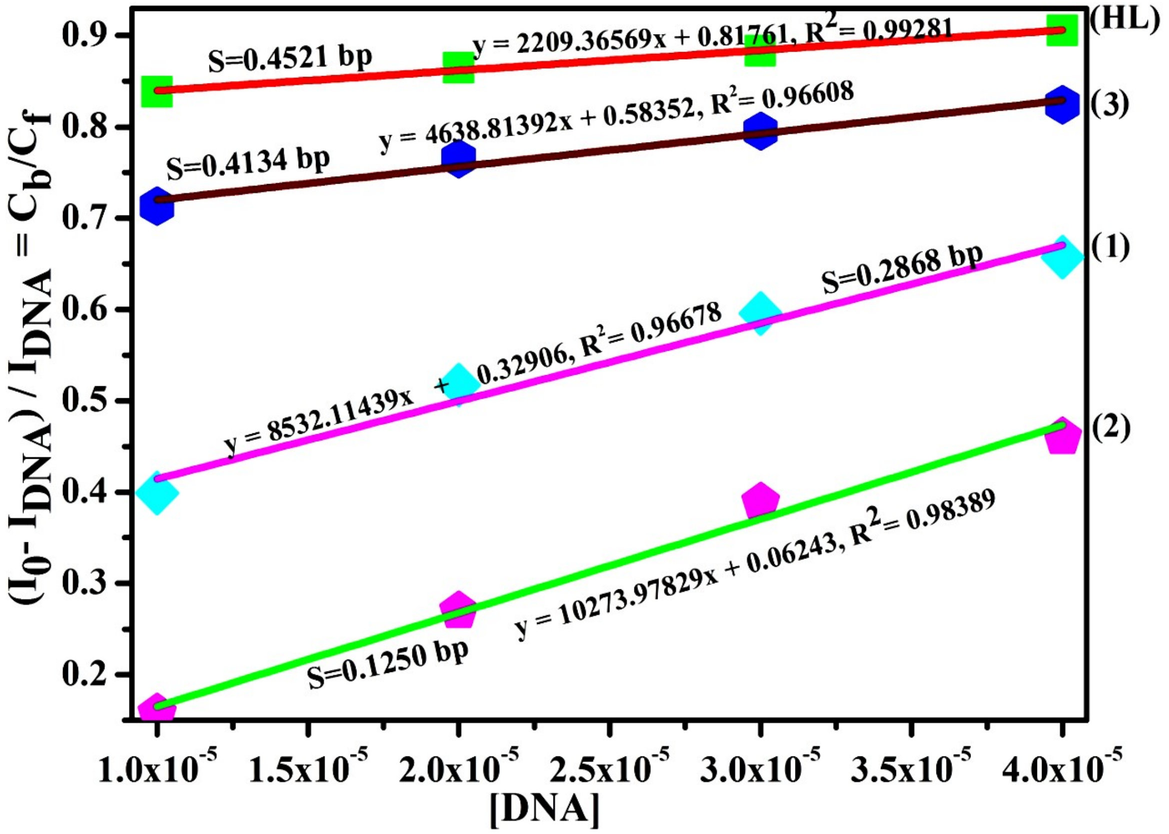

Fig.S33. Plot of C_b/C_f *vs* [DNA] for determination of binding site size (S) and binding constant for ligand (**HL**) and mixed ligand complexes (**1**–**3**) with DNA by Method II.

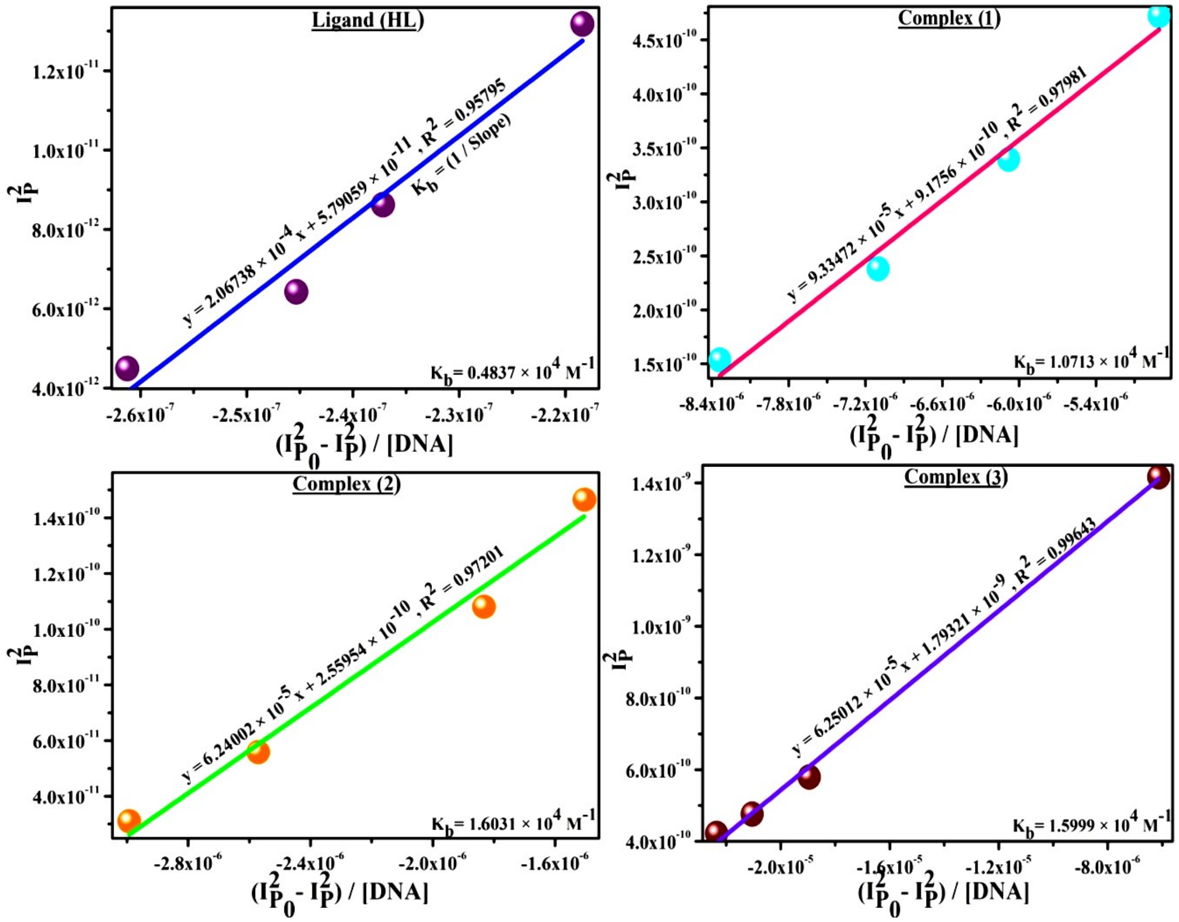

Fig.S34. Plot of Ip^2 *vs* $(I_{po}^2 - I_p^2)$ / [DNA] to evaluate the binding constants for ligand (**HL**) and complexes (**1**–**3**) with DNA by Method III.

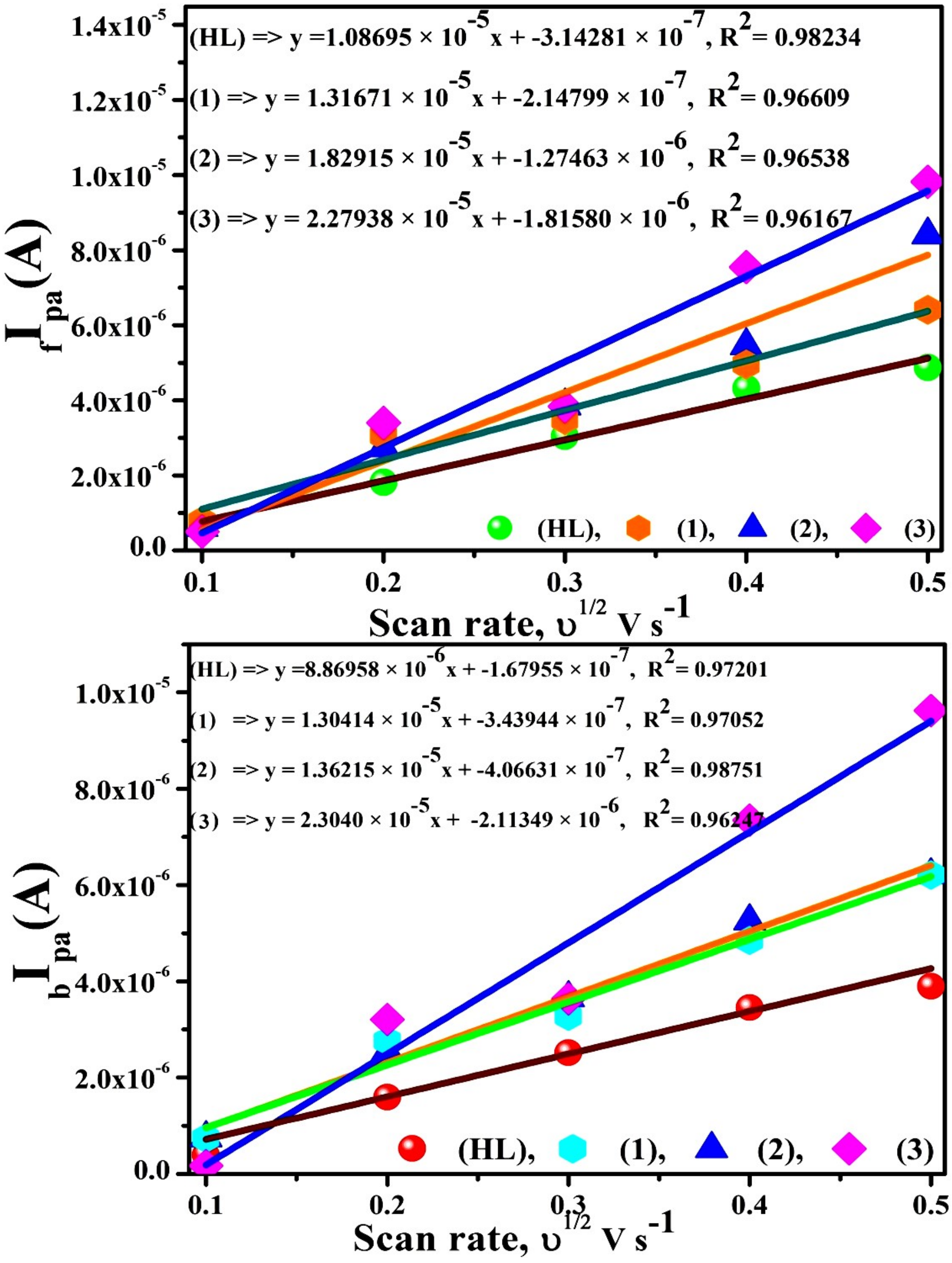

Fig.S35. Plots of $_f$Ipa *vs*. $v^{1/2}$ and $_b$Ipa *vs*. $v^{1/2}$ for the determination of diffusion coefficients (D_0) of the free compounds in the absence and presence of DNA at scan rates 0.01–0.3 V/s.

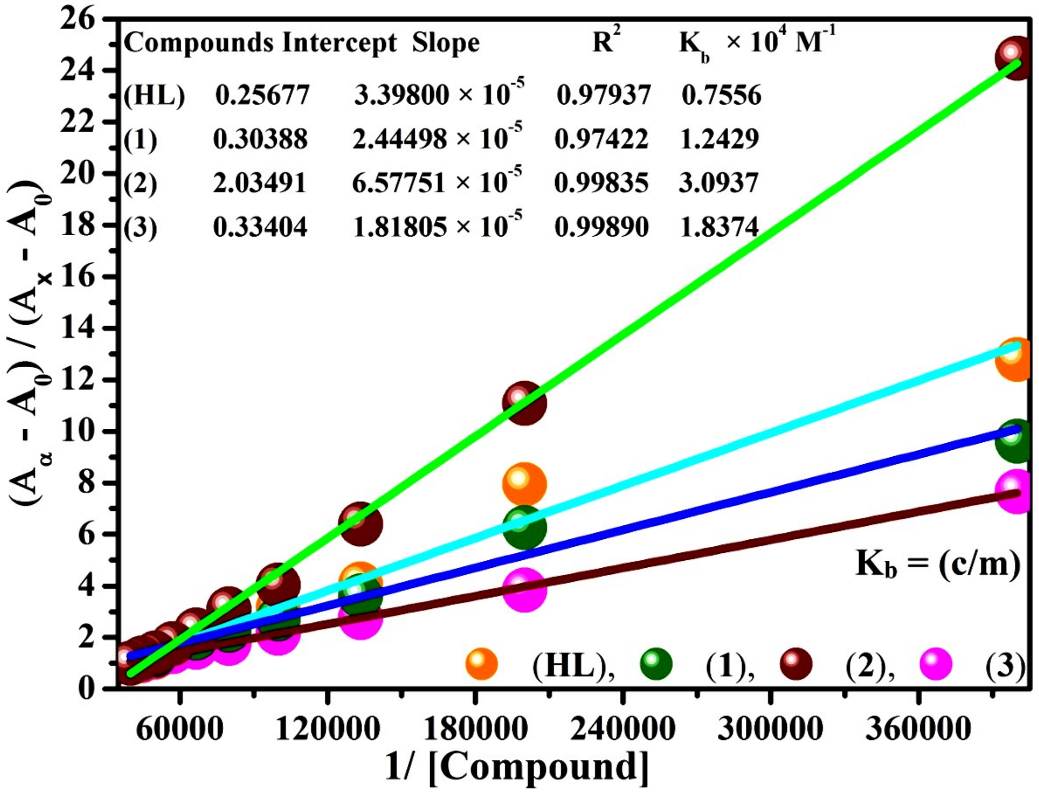

Fig.S36. Benesi-Hildebrand linear plot [(A_∞–A_0) / (A_x–A_0)] *vs* 1 / [compound] determining binding constant for ligand (**HL**) and mixed ligand complexes (**1**–**3**) – BSA at 298 K.

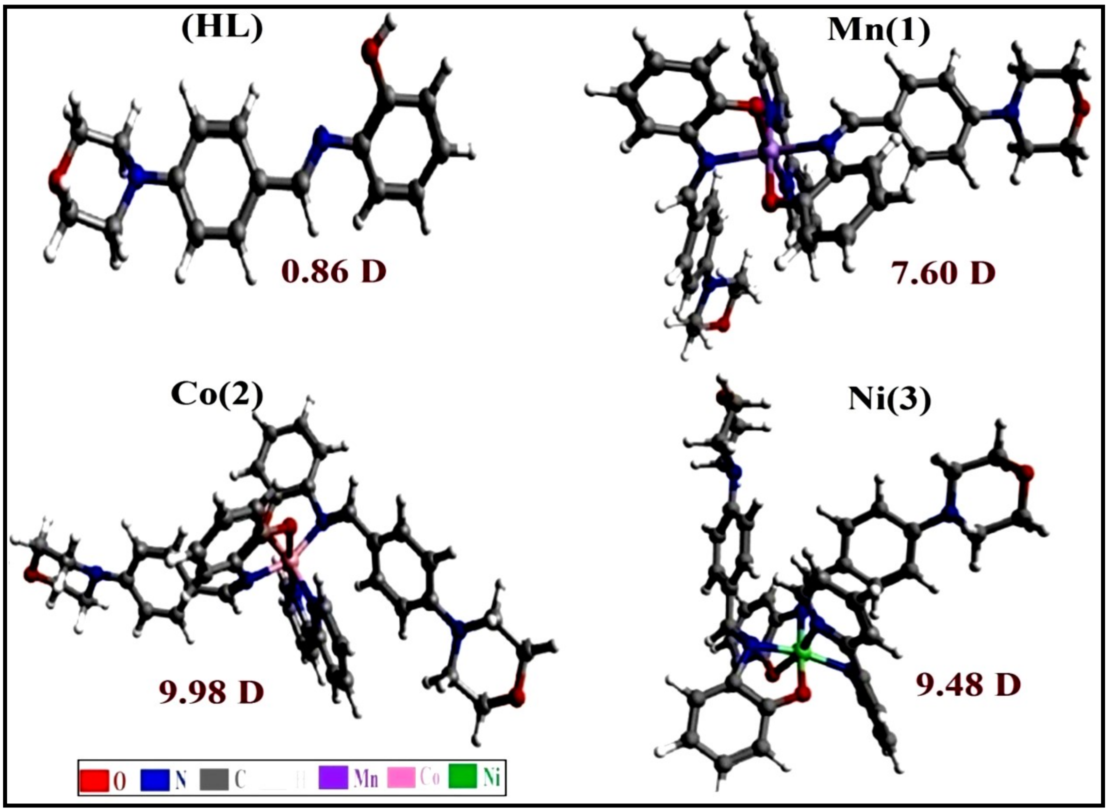

Fig.S37. The optimized geometries for the free ligand (**HL**) and its mixed ligand complexes (**1–3**).

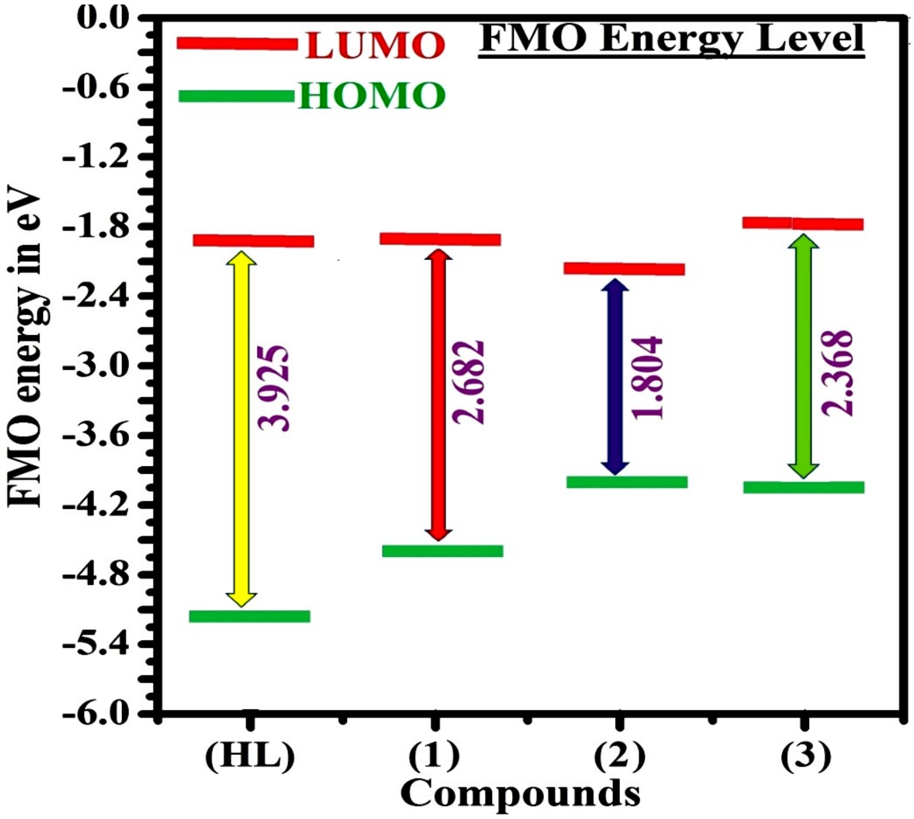

Fig.S38. FMO energy level diagram by DFT computation for all compounds.

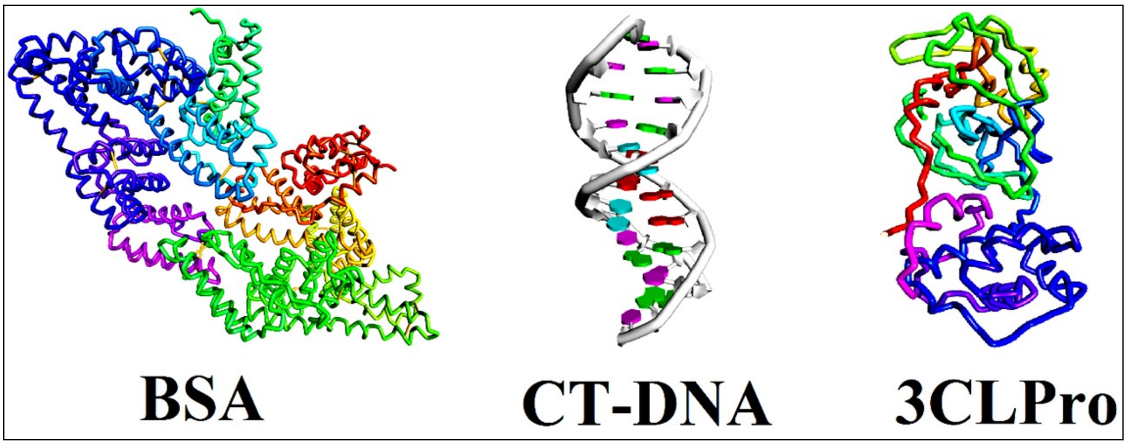

Fig.S39. 3D model of the host biomolecules used for docking calculations. From left to right: BSA, CT-DNA and 3CLPro.

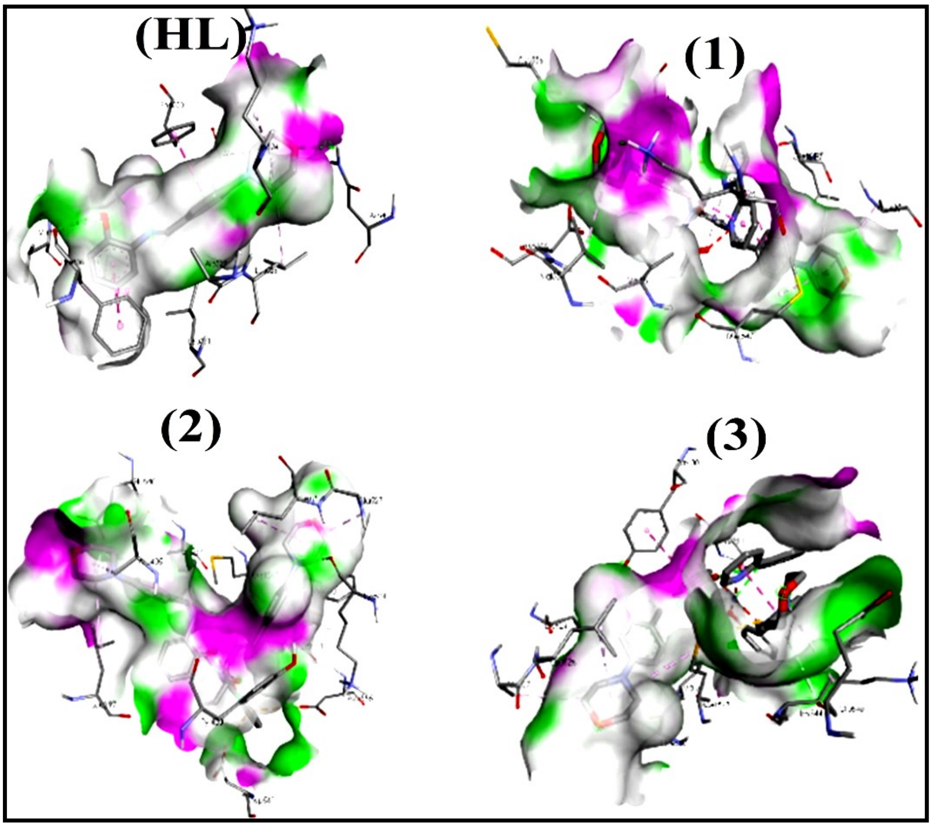

Fig.S40. Noncovalent interactions in the active site of the BSA protein. The pink and green colors denote H-bond acceptor and donor sites respectively.

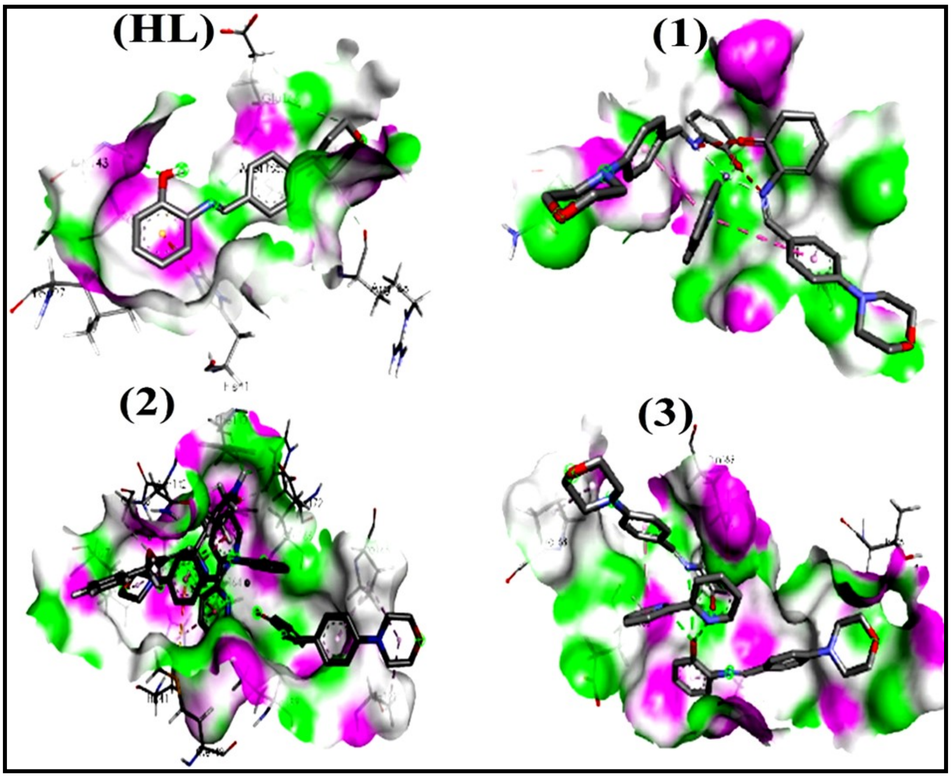

Fig.S41. Noncovalent interactions in the active site of the 3CLPro protein. The pink and green colors denote H-bond acceptor and donor sites respectively.

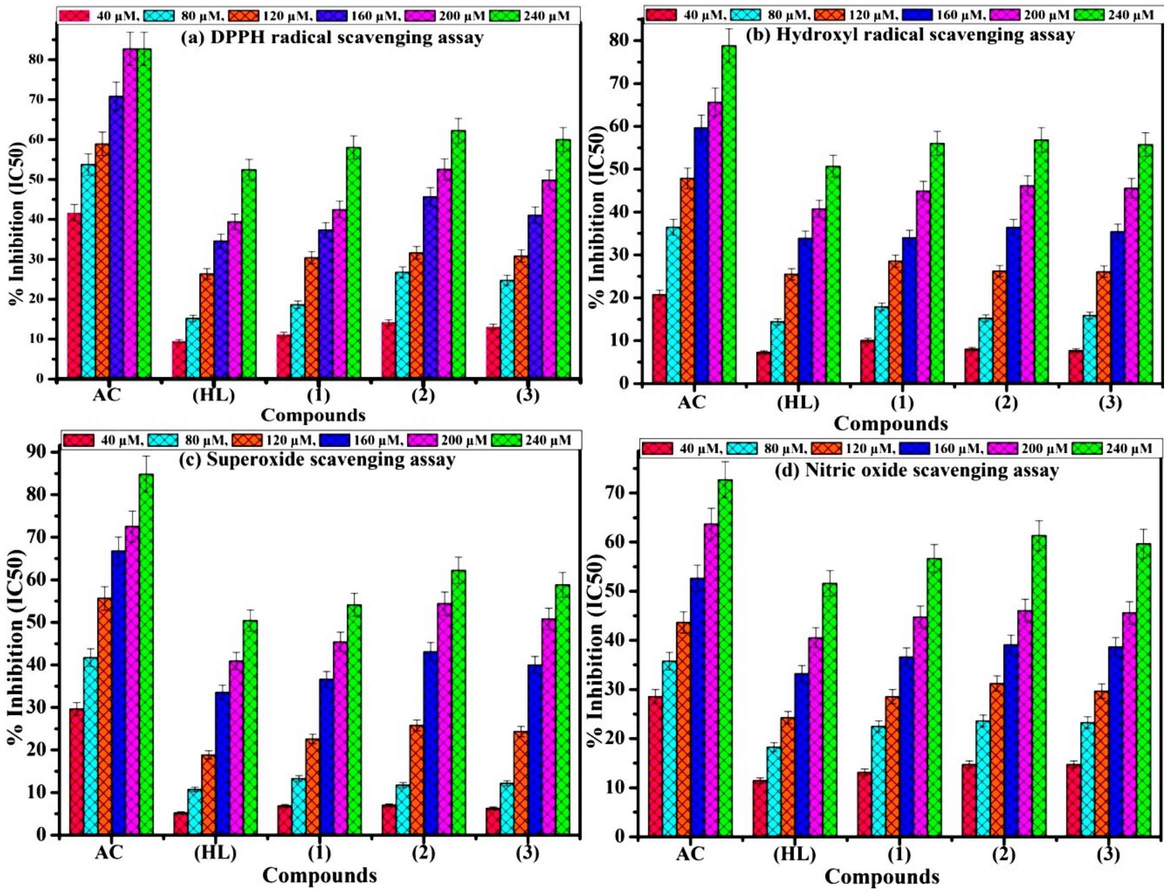

Fig.S42 (a–d). % inhibition-IC_{50} of of (**a**) DPPH, (**b**) Hydroxyl, (**c**) Superoxide and (**d**) Nitric oxide scavenging assay for ligand (**HL**), mixed ligand complexes (**1–3**) and standard ascorbic acid (AC-control at various concentration (40, 80,120,160, 200, 240 µM).

$$Scavanging\ (\%) = \left[\frac{(A_0 - A_S)}{A_0}\right] \times\ 100$$

(**43**); where A_0 and A_S are denoted as the absorbance intensity of free DPPH (control) and DPPH mixed substances in ethanol, respectively. Error limits ± 2.5 – 5.0 % ($P \preccurlyeq 0.05$).

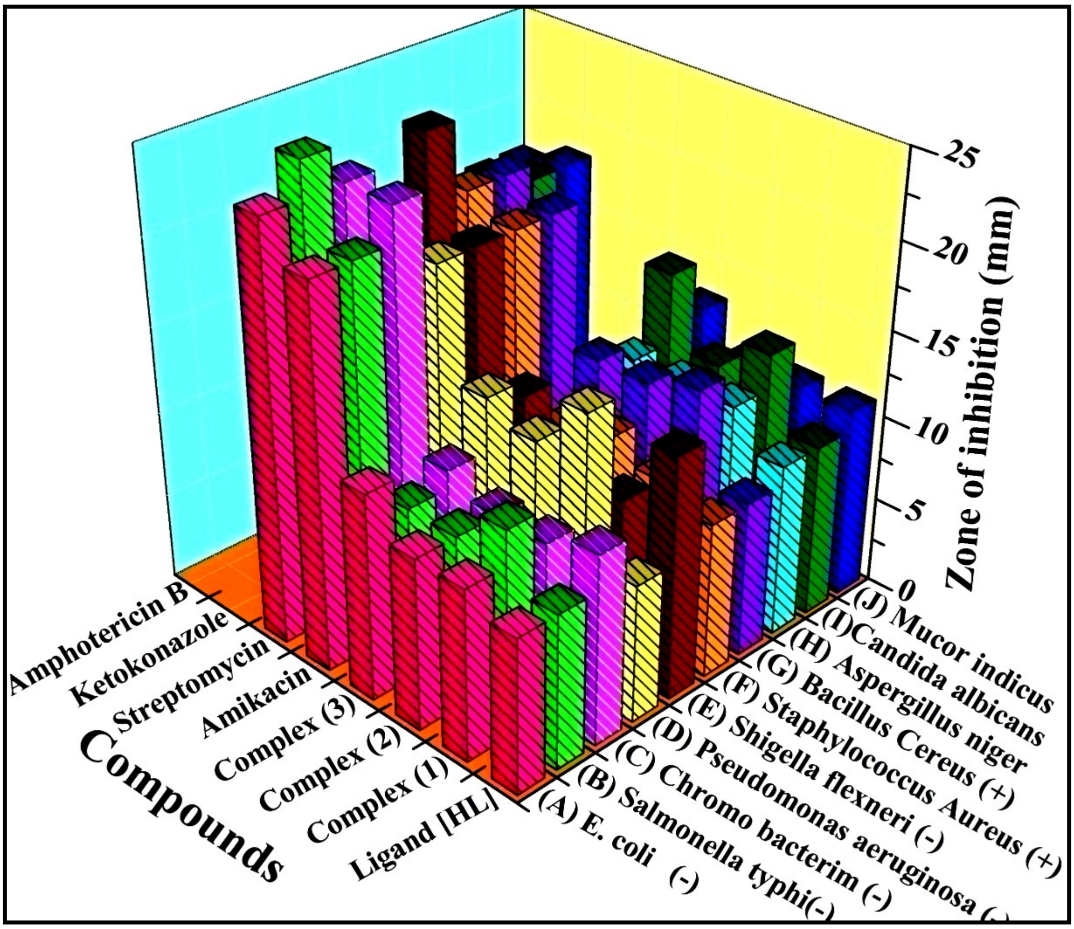

Fig.S43. Agar disc diffusion technique histogram comparing the antimicrobial effects for all substances. Error limits ± 2.5 – 5.0 % (P ≤ 0.05).

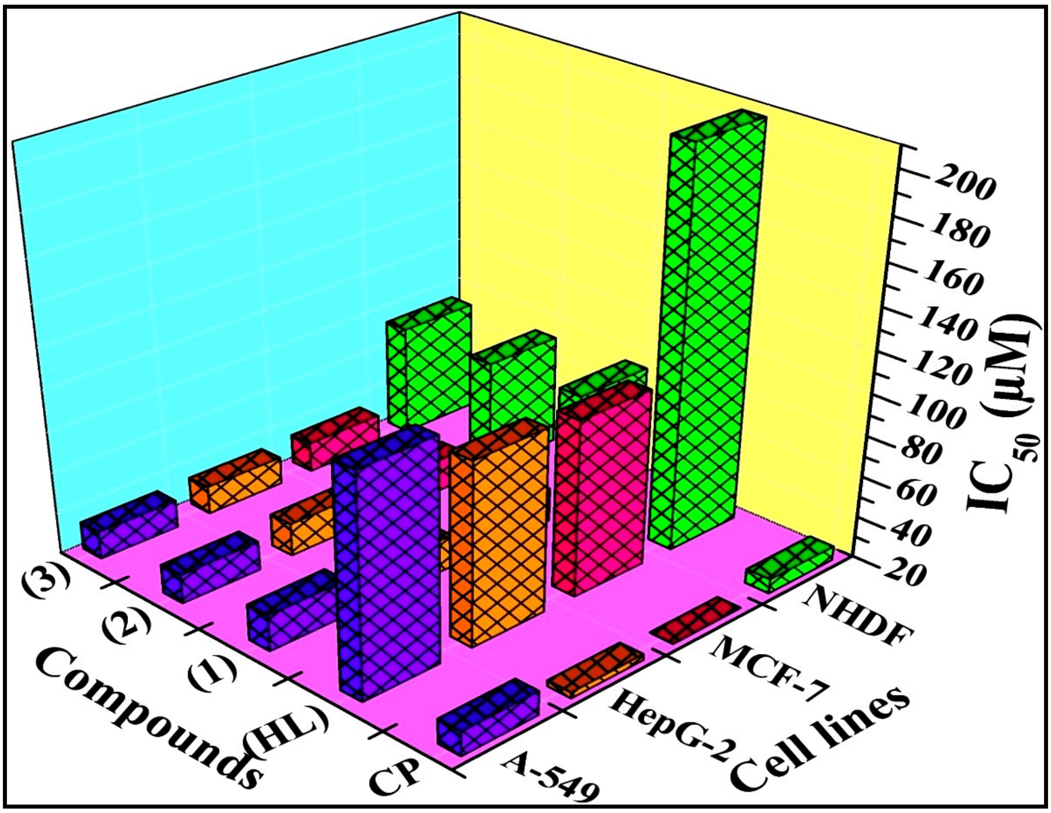

Fig.S44. The evaluation of the cytotoxic properties (IC_{50}, μM) of ligand (**HL**) and its complexes (**1–3**) against cancer and normal cell lines in comparison to the standard medication cisplatin (CP). Error limits ± 2.5 – 5.0 % ($P \preccurlyeq 0.05$).

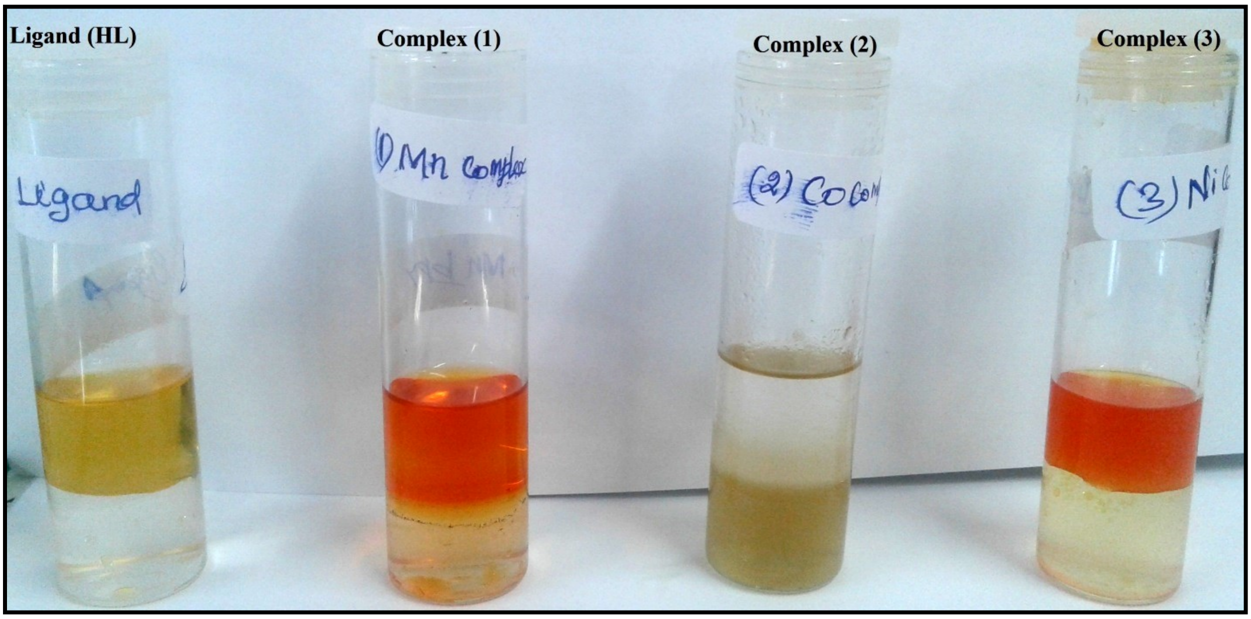

Fig.S45. n-octanol/deionized water phase of complexes (**1–3**) and free ligand (**HL**) for determination of partition coefficient (log $P_{o/w}$) by shake flask method.

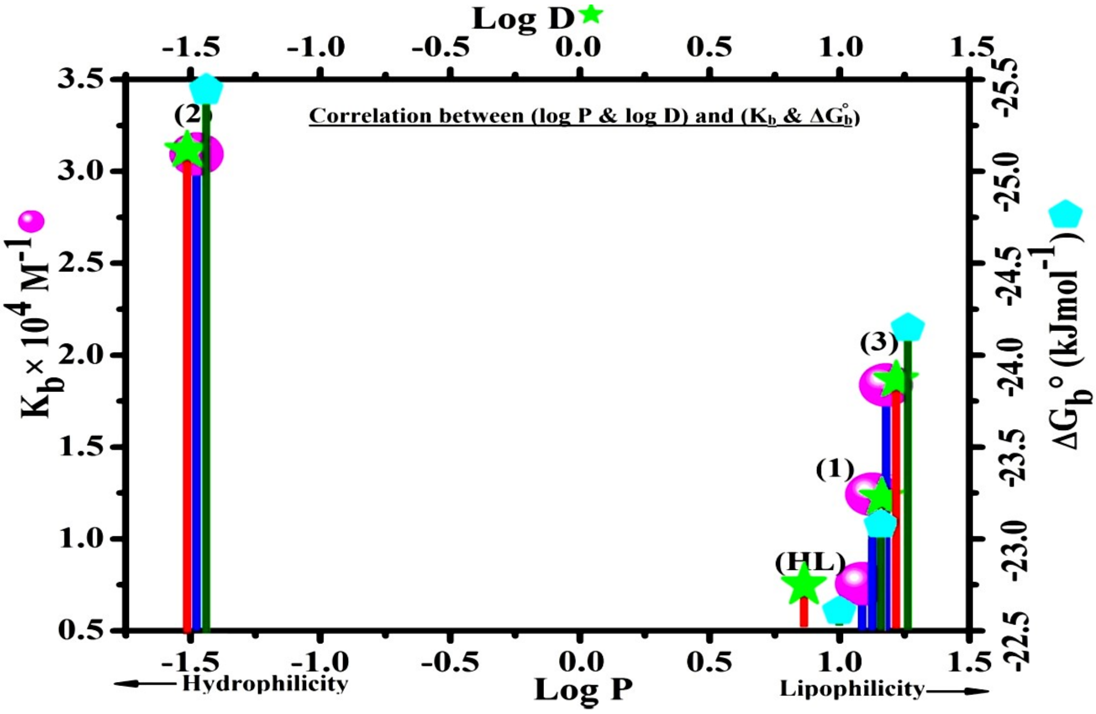

Fig.S46. The correlation plot between partition coefficient (log P) & distribution coefficient (log D) *vs* BSA binding constants (K_b) & Gibb's free energy Change (ΔG_b°) for complexes (**1–3**) and free ligand (**HL**).

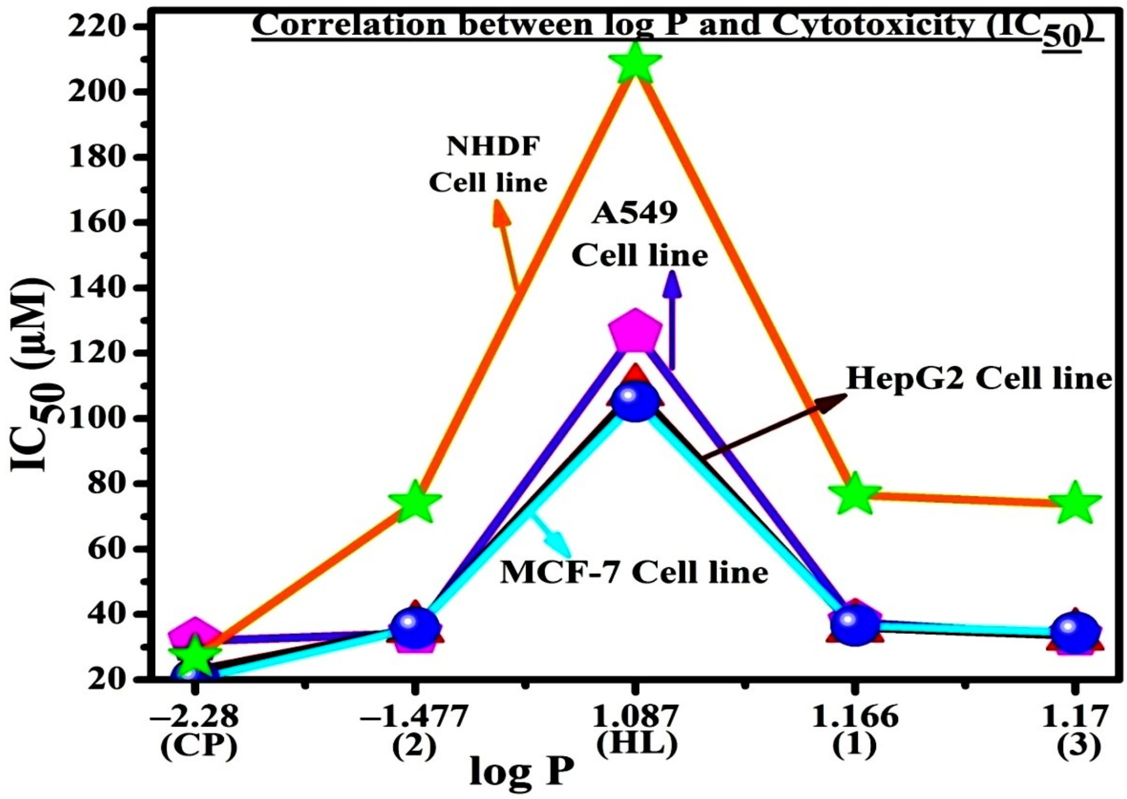

Fig.S47. Cytotoxicity (IC_{50}, μM) of ligand (**HL**) and its complexes (**1–3**) as a function of their partition coefficient (log P) comparison with standard anticancer drug cisplatin (**CP**).

Supplementary Tables

Table S12 IC_{50} values of DPPH radical scavenging assay at 517 nm.

Compounds	% Inhibition (IC_{50})					
	**40 µM**	**80 µM**	**120 µM**	**160 µM**	**200 µM**	**240 µM**
Ascorbic acid	41.63	53.74	58.95	70.86	82.75	85.65
(**HL**)	09.45	15.21	26.36	34.56	39.41	52.45
(**1**)	11.16	18.62	30.41	37.32	42.47	58.05
(**2**)	14.20	26.76	31.64	45.67	52.54	62.23
(**3**)	13.08	24.74	30.86	41.06	49.83	60.02

$Scavanging\ (\%) = \left[\frac{(A_0 - A_S)}{A_0}\right] \times 100$, Where, A_0 → absorbance of the control or (DPPH alone in ethanol) and A_S → absorbance of the sample or (Mixture of DPPH and compounds in ethanol). Error limits ± 2.5 - 5.0.

Table S13 IC_{50} values of Hydroxyl radical scavenging assay at 230 nm.

Compounds	% Inhibition (IC_{50})					
	**40 µM**	**80 µM**	**120 µM**	**160 µM**	**200 µM**	**240 µM**
Ascorbic acid	20.73	36.46	47.87	59.65	65.64	78.83
(**HL**)	07.26	14.43	25.52	33.86	40.73	50.68
(**1**)	10.08	17.89	28.56	34.04	44.92	56.03
(**2**)	08.02	15.24	26.23	36.41	46.17	56.85
(**3**)	07.67	15.86	26.12	35.43	45.56	55.75

Table S14 IC_{50} values of Superoxide scavenging assay at 590 nm.

Compounds	% Inhibition (IC_{50})					
	**40 µM**	**80 µM**	**120 µM**	**160 µM**	**200 µM**	**240 µM**
Ascorbic acid	29.64	41.72	55.63	66.75	72.55	84.85
(**HL**)	05.23	10.74	18.85	33.55	40.91	50.42
(**1**)	06.85	13.32	22.56	36.63	45.42	54.12
(**2**)	07.02	11.73	25.81	43.12	54.42	62.24
(**3**)	06.27	12.17	24.31	40.01	50.78	58.83

Table S15 IC_{50} values of Nitric oxide scavenging assay at 546 nm.

Compounds	% Inhibition (IC_{50})					
	**40 µM**	**80 µM**	**120 µM**	**160 µM**	**200 µM**	**240 µM**
Ascorbic acid	28.55	35.75	43.65	52.68	63.72	72.73
(**HL**)	11.42	18.26	24.28	33.21	40.53	51.62
(**1**)	13.14	22.48	28.54	36.64	44.75	56.69
(**2**)	14.72	23.62	31.21	39.12	46.06	61.34
(**3**)	14.72	23.28	29.66	38.63	45.60	59.65
